# Synthesis of Ester-Containing Chroman-4-Ones via Cascade Radical Annulation of 2-(Allyloxy)Arylaldehydes with Oxalates under Metal Free Conditions

**DOI:** 10.3390/ijms24055028

**Published:** 2023-03-06

**Authors:** Sha Peng, Jiao Liu, Ze-Qun Jiang, Lin Yuan, Xiao-Wen Liu, Long-Yong Xie

**Affiliations:** College of Chemistry and Bioengineering, Hunan University of Science and Engineering, Yongzhou 425100, China

**Keywords:** chroman-4-ones, alkoxycarbonylation, 2-(allyloxy)arylaldehydes, cascade annulation, radical reaction

## Abstract

A convenient and practical method for the synthesis of bioactive ester-containing chroman-4-ones through the cascade radical cyclization of 2-(allyloxy)arylaldehydes and oxalates is described. The preliminary studies suggest that an alkoxycarbonyl radical might be involved in the current transformation, which was generated via the decarboxylation of oxalates in the presence of (NH_4_)_2_S_2_O_8_.

## 1. Introduction

**Figure 1:**
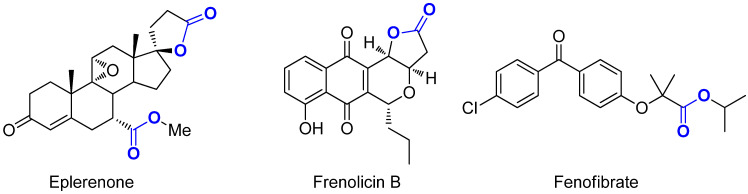
Selective pharmaceutical molecules containing ester motifs.

The construction of ester-containing compounds is among the most valuable transformations in modern organic synthesis, given the abundance of these motifs in many functional materials, natural products, and pharmaceuticals [1,2,3,4], to name a few (Figure 1). They are also important building blocks which can be converted into versatile functional groups such as carboxyl, hydroxymethyl, aldehyde, amide, etc. [5,6]. Consequently, developing novel and practical methods for the synthesis of organic molecules containing such structural moiety has received extensive attention [7,8,9,10]. Traditionally, the synthesis of esters relies on the esterification of carboxylic acids, acyl chlorides or anhydrides with various alcohols. These approaches need the pre-installation of a carboxyl group in the substrate. Doubtless, the direct introduction of an ester group to organic compounds represents a more effective strategy. In this context, transition-metal-catalyzed alkoxycarbonylation using carbon monoxide and alcohol as ester sources might be an attractive and alternative protocol [11,12,13]. However, this method often suffers from the use of toxic CO and high-pressure equipment. Thus, the development of alkoxycarbonylation reactions with readily available and easily handled esterification reagents other than carbon monoxide is still in high demand.

In recent years, cascade radical annulation has emerged as an efficient strategy for the synthesis of functionalized heterocycles. A variety of novel and practical radicals triggered cascade annulation reactions have been widely reported [14,15,16,17,18,19]. Among these, ester radical induced annulation reactions have also gained great attention [20,21]. For instance, in 2013, Du and Li reported an iron-catalyzed oxidative radical cascade annulation of *N*-aryl acrylamides with carbazates toward alkoxycarbonylated oxindoles [22]. In 2014, Zhu et al., reported an iron-catalyzed radical alkoxycarbonylation of 2-isocyanobiphenyl with carbazates leading to phenanthridine-6-carboxylates [23]. In 2017, Sun and coworkers reported a visible light induced cascade radical cyclization reaction toward ester-functionalized pyrido[4,3,2-gh]phenanthridind derivatives using carbazates as the ester sources [24]. Recently, Wu and Chen reported an Ir(ppy)_3_ catalyzed alkoxycarbonylation/cyclization reaction of *N*-acryloyl benzamides with alkyloxalyl chlorides under visible light irradiation [25]. Despite these achievements, developing more alkoxycarbonyl radical triggered cascade cyclization reactions is still highly desirable.

**Scheme 1:**
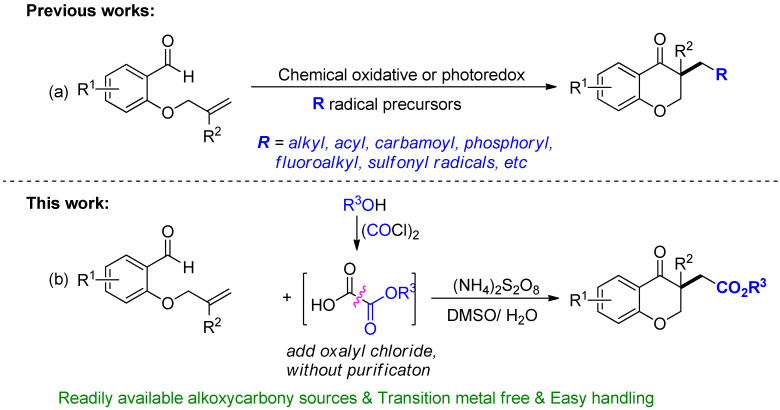
Radical triggered cascade cyclization of 2-(allyloxy)arylaldehydes (**a**) Diverse radical triggered cascade cyclization of 2-(allyloxy)arylaldehydes (**b**) Alkoxycarbonyl radical triggered cascade cyclization of 2-(allyloxy)arylaldehydes.

On the other hand, chroman-4-ones are among the ubiquitous structural motifs that occur in natural products, pharmaceuticals and biologically active compounds. They exhibit a variety of physiological and biological activities, such as antibacterial, antioxidant, anti-HIV, and SIRT2 inhibiting properties [26,27,28,29,30]. For example, 8-bromo-6-chloro-2-pentylchroman-4-one was selected as a potent inhibitor of SIRT2 with an IC_50_ of 1.5 μM in a reported work in the literature [30]. In the past few years, various radical cascade annulation reactions of 2-(allyloxy)arylaldehydes triggered by diverse radicals, such as alkyl, acyl, phosphoryl, trifluoromethyl and sulfonyl, for the synthesis of valuable chroman-4-one derivatives have been well established (Scheme 1a) [31,32,33,34,35,36,37,38,39,40,41,42]. Nevertheless, to the best of our knowledge, an alkoxycarbonyl radical triggered cascade radical cyclization to synthesize ester-functionalized chroman-4-ones has never been reported. Herein, we disclose a (NH_4_)_2_S_2_O_8_-mediated protocol for the selective intramolecular decarboxylative radical cyclization of 2-(allyloxy)arylaldehydes with oxalates for the rapid building of a variety of ester-containing alkyl-substituted chroman-4-ones (Scheme 1b). It is worth mentioning that oxalates can be easily obtained via the condensation of readily available alcohols and oxalyl chloride, followed by in situ hydrolysis without tedious column chromatography, making the present method much more practical and attractive to give various ester-containing chroman-4-ones.

## 2. Results and Discussion

Initially, a model reaction of 2-(allyloxy)benzaldehyde (**1a**) and 2-methoxy-2-oxoacetic acid (**2a**) was carried out to investigate the reaction conditions (Table 1). When the reaction was performed in DMSO at 80 °C under N_2_ atmosphere for 24 h employing (NH_4_)_2_S_2_O_8_ (3 equiv.) as the oxidant, the desired product, methyl 2-(4-oxochroman-3-yl)acetate (**3aa**), was obtained in 71% isolated yield (entry 1). Inspired by the above result, some other common solvents including DMF, CH_3_CN, DCE, THF and H_2_O were also screened. To our surprise, only DMSO was efficient for the present transformation and no desired **3aa** was observed with other examined solvents (entries 2–6). Furthermore, several mixed solvents consisting of different volume ratios of DMSO and H_2_O were tested (entries 7–10). The results revealed that a trace amount of H_2_O was more efficient for the current reaction (entry 7 vs. entry 1), and **3aa** was isolated with 76% yield in DMSO/H_2_O (500:1). Next, various oxidants including K_2_S_2_O_8_, Na_2_S_2_O_8_, TBHP, Selectfluor and PhI(OAc)_2_ were also investigated (entries 11–15). In sharp contrast to (NH_4_)_2_S_2_O_8_, no desired product **3aa** was detected with the other examined oxidants. Moreover, the influence of reaction temperature to the reaction was then tested. Elevating the temperature from 80 °C to 90 °C, the yield of **3aa** increased from 76% to 81% (entry 16 vs. entry 7); further raising the temperature to 100 °C gave a slightly reduced yield of **3aa** (entry 17). When the reaction temperature was reduced from 90 °C to 70 °C, **3aa** was obtained with an obviously decreasing yield (entry 18). In addition, reducing the amount of (NH_4_)_2_S_2_O_8_ or **2a** to 2 equiv. (based on **1a**), the desired **3aa** was obtained in 57 and 64% yields, respectively (entries 19–20). Moreover, in the absence of any oxidant, no reaction occurred, suggesting that oxidant was crucial for the current reaction (entry 21).

**Scheme 2:**
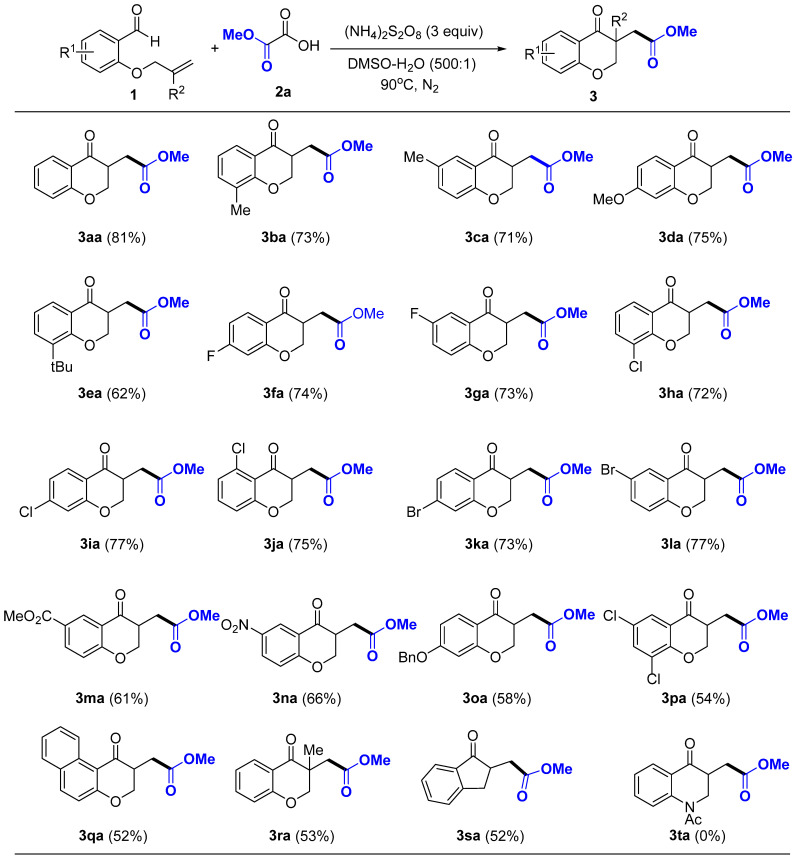
Preparation of **3aa**–**3ta**. Conditions: **1** (0.3 mmol), **2a** (0.9 mmol), (NH_4_)_2_S_2_O_8_ (0.9 mmol), DMSO-H_2_O (*v*/*v* 500/1, 1.8 mL), 90 °C, N_2_, 24 h. Isolated yields.

With the established optimal reaction conditions (Table 1, entry 16), we first investigated the generality of this cascade annulation reaction by employing various substituted 2-(allyloxy)arylaldehydes with 2-methoxy-2-oxoacetic acid (**2a**). As depicted in Scheme 2, the substrates bearing either electron-donating substitutions (Me, OMe, t-Bu and OBn) or electron-withdrawing substitutions (F, Cl, Br and CO_2_Me) at the different positions of the benzene ring all reacted smoothly to give the desired products in moderate to good yields (**3aa**–**3na**). Furthermore, the di-substituted and naphthalene derived substrates were also transformed into the desired products **3pa** and **3qa** in moderate yields. Moreover, substrate **1r**, bearing a methyl substitution close to C=C bond and 2-allylbenzaldehyde **1s**, also reacted well, giving the corresponding products **3ra** and **3sa** in 53 and 52% yields, respectively. However, *N*-allyl-*N*-(2-formylphenyl)acetamide **1t** as a substrate failed to give any of the desired product under the current condition (**3ta**).

**Scheme 3:**
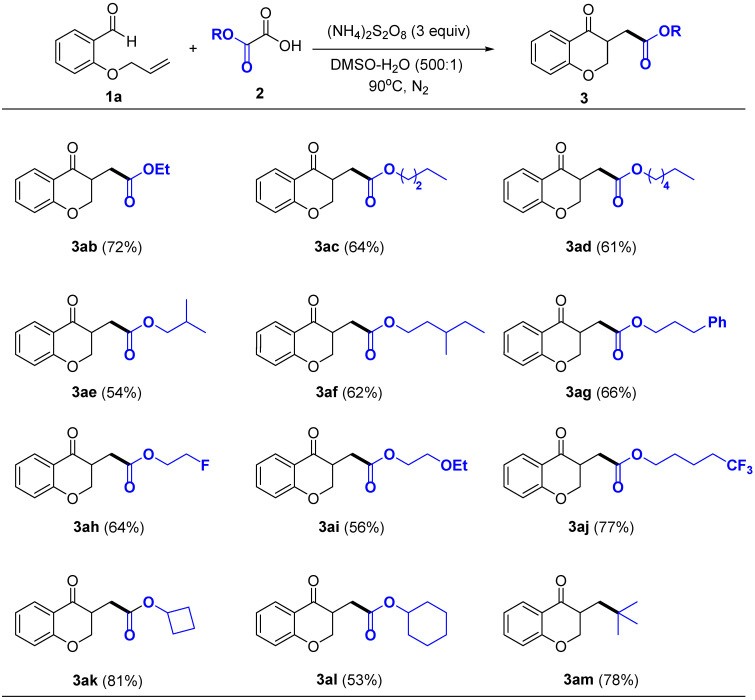
Preparation of **3ab**–**3am**. Conditions: **1a** (0.3 mmol), **2** (0.9 mmol), (NH_4_)_2_S_2_O_8_ (0.9 mmol), DMSO-H_2_O (*v*/*v* 500/1, 1.8 mL), 90 °C, N_2_, 24 h. Isolated yields.

Next, the reaction scope with respect to oxalates was evaluated. As shown in Scheme 3, various oxalates (**2b**–**2j**) with varied aliphatic chains containing diverse substitutions, including alkyl (**2b**–**2f**), phenyl (**2g**), fluorine atom (**2h**), alkoxyl (**2i**) and trifluoromethyl (**2j**), proceeded well to deliver the desired products **3ab**–**3aj** in good yields. In addition, cyclic substituted substrates were also compatible with the reaction, giving **3ak** and **3al** in 81 and 53% yields, respectively. Moreover, using 2-(tert-butoxy)-2-oxoacetic acid **2m** as a substrate, a more stable tert-butyl radical was generated via a double decarboxylation process, which further reacted with **1a** to access the corresponding product **3am** in 78% yield.

**Scheme 4:**
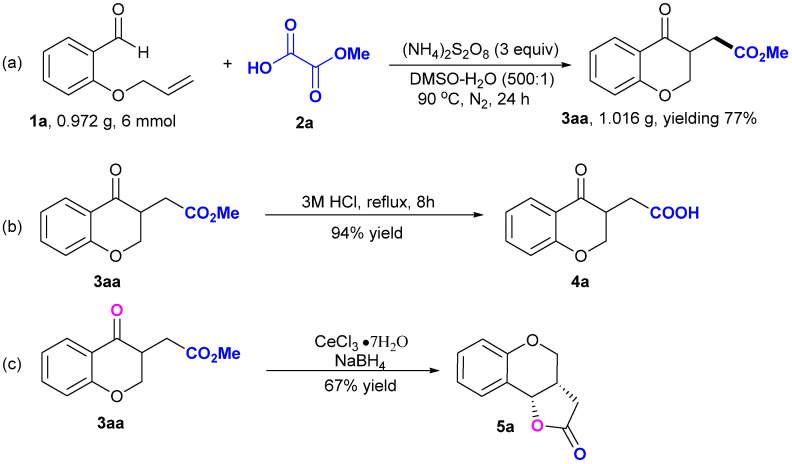
Gram-scale synthesis of **3aa** and its transformations (**a**) Gram-scale synthesis of 3aa; (**b**) Hydrolysis of **3aa** under acidic conditions**;** (**c**) One pot synthesis of cis-lactone 5a.

To illustrate the synthetic application of this method, a gram scale experiment of **1a** and **2a** was performed under standard conditions and the desired product **3aa** was obtained in 77% yield (Scheme 4a). Additionally, further valuable transformations of **3aa** were carried out. First, **3aa** could be smoothly hydrolyzed to carboxyl-containing compound **4a** under acidic conditions with an excellent yield (Scheme 4b). Moreover, the treatment of **3aa** with CeCl_3_ and NaBH_4_ gave the cis-lactone **5a** in 67% yield as the exclusive product [43,44] (Scheme 4c).

**Scheme 5:**
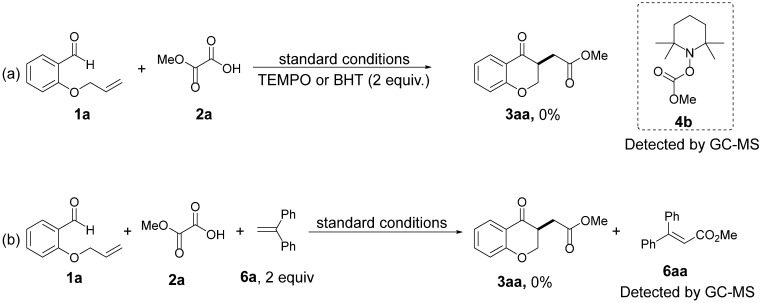
Control experiments (**a**) Radical inhibition experiment using TEMPO or BHT as radical inhibitor; (**b**) Trapping of the methyl ester radical.

To better understand this cascade cyclization process, some control experiments were performed as shown in Scheme 5. When the model reaction was conducted under the standard reaction conditions with the addition of 2 equiv. of BHT or TEMPO as the free radical inhibitor, no desired product **3aa** was observed and TEMPO-adduct **4b** was detected by GC-MS analysis (Scheme 5a), indicating that a free-radical pathway might be involved in the present transformation. Moreover, a reaction of **1a** and **2a** was performed in the presence of 1,1-diphenylethene under standard conditions, the radical adduct **6aa** resulting from the trapping of the initial methyl ester radical by **6a** was observed**,** which further confirmed that the current reaction was triggered by the alkoxycarbonyl radical generated through the decarboxylation of oxalates during the reaction (Scheme 5b).

**Scheme 6:**
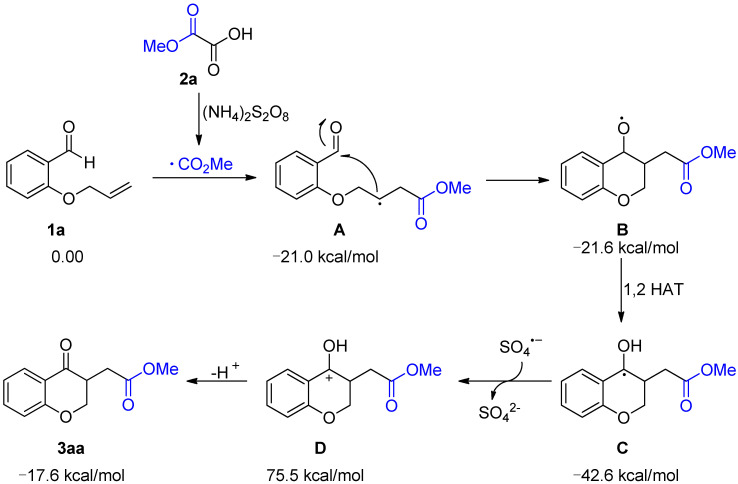
possible mechanism.

Based on the density functional theory (DFT) calculations (for details, see the Supporting Information), our preliminary control experiments and the relevant reports from the literature [25,45,46], we speculated a possible reaction pathway, as shown in Scheme 6. First, a radical anion SO_4_^−•^ generates via the decomposition of (NH_4_)_2_S_2_O_8_ in DMSO; meanwhile, SO_4_^−•^ captures a hydrogen atom from 2-methoxy-2-oxoacetic acid (**2a**) to form a methyl ester radical via a decarboxylative process. Then, the methyl ester radical attacks the C=C bond of **1a** to give radical intermediate **A** (−21.0 kcal/mol), which undergoes further cyclization to afford an oxygen radical **B** (−21.6 kcal/mol). The radical **B** then undergoes a 1,2-hydrogen atom transfer (HAT) process to afford radical **C** (−42.6 kcal/mol). Furthermore, in the presence of radical anion SO_4_^−•^, the intermediate **C** is transformed to a carbocation intermediate D (75.5 kcal/mol); the activation free energy of this process is 118.1 kcal/mol (C→D), which might be the rate-determining step. Finally, a favorable deprotonation process occurs to deliver the desired product **3aa** (−17.6 kcal/mol).

## 3. Materials and Methods

### 3.1. General Information

Unless otherwise specified, all chemicals were purchased from commercial suppliers and directly used as received without additional purification. Column chromatography was carried out with silica gel (200–300 mesh) to purify products, using proper solvents as the eluent system. NMR spectra were recorded at 400 MHz for ^1^H NMR spectra and 100 MHz for ^13^C NMR spectra by using a German Bruker Avance 400 spectrometer. Chemical shifts are quoted in parts per million referenced to the appropriate solvent peak (^1^H NMR: CDCl_3_ 7.26 ppm, ^13^C NMR: CDCl_3_ 77.0 ppm). The following abbreviations were used to describe peak splitting patterns when appropriate: s = singlet, d = doublet, t = triplet, q = quartet, m = multiplet. Mass spectra were performed on a spectrometer operating on ESI-TOF.

### 3.2. General Procedure for the Synthesis of Target Products **3**

An oven-dried 10 mL reaction tube was charged with 2-(allyloxy)arylaldehyde **1** (0.3 mmol, 1 eq), oxalates **2** (0.9 mmol, 3 eq) and (NH_4_)_2_S_2_O_8_ (0.9 mmol, 3 eq) in a DMSO aqueous solution (1.8 mL, V_DMSO_/V_H2O_ = 500/1) with a magnetic stirring bar. The mixture was then stirred at 90 °C under N_2_ atmosphere conditions for about 24 h. The reaction was monitored by TLC. After completion, water (10 mL) was added and the mixture was extracted with EtOAc (10 mL × 3); the solvent was then removed under vacuum. The residue was purified by flash column chromatography using a mixture of petroleum ether and ethyl acetate as eluent to give the desired products **3**.

### 3.3. Gram-Scale Synthesis of **3aa**

An oven-dried 100 mL round-bottom flask was charged with 2-(allyloxy)benzaldehyde **1a** (0.972 g, 6 mmol), 2-methoxy-2-oxoacetic acid **2a** (1.872 g, 18 mmol) and (NH_4_)_2_S_2_O_8_ (4.104 g, 18 mmol) in a DMSO aqueous solution (36 mL, V_DMSO_/V_H2O_ = 500/1) with a magnetic stirring bar. The mixture was then stirred at 90 °C under N_2_ atmosphere conditions for about 24 h. The reaction was monitored by TLC. After completion, water (30 mL) was added and the mixture was extracted with EtOAc (40 mL × 3); the solvent was then removed under vacuum. The residue was purified by flash column chromatography using a mixture of petroleum ether and ethyl acetate as eluent to give 1.016 g of **3aa**, yielding 77%.

### 3.4. Characterization Data of Products **3aa**–**3sa** and **3ab**–**3am**

methyl 2-(4-oxochroman-3-yl)acetate (**3aa**):

^1^H NMR (400 MHz, Chloroform-d) δ 7.88 (d, *J* = 7.8 Hz, 1 H), 7.55–7.41 (m, 1 H), 7.08–6.94 (m, 2 H), 4.60 (dd, *J* = 11.2, 5.3 Hz, 1 H), 4.29 (t, *J* = 11.6 Hz, 1 H), 3.73 (s, 3 H), 3.34 (td, *J* = 12.8, 5.1 Hz, 1 H), 2.94 (dd, *J* = 17.0, 4.9 Hz, 1 H), 2.43 (dd, *J* = 17.0, 8.1 Hz, 1 H); ^13^C NMR (100 MHz, Chloroform-d) δ 192.6, 171.8, 161.7, 136.0, 127.4, 121.5, 120.4, 117.8, 70.2, 52.0, 42.5, 30.1; HRMS (ESI): *m*/*z* [M + H]^+^ calcd for C_12_H_13_O_4_: 221.0808; found: 221.0811.

methyl 2-(8-methyl-4-oxochroman-3-yl)acetate (**3ba**):

^1^H NMR (400 MHz, Chloroform-d) δ 7.73 (d, *J* = 7.7 Hz, 1 H), 7.33 (d, *J* = 6.9 Hz, 1 H), 6.91 (t, *J* = 7.4 Hz, 1 H), 4.63 (dd, *J* = 11.0, 5.1 Hz, 1 H), 4.27 (t, *J* = 11.6 Hz, 1 H), 3.73 (s, 3 H), 3.31 (dt, *J* = 12.2, 6.2 Hz, 1 H), 2.94 (dd, J = 17.0, 4.7 Hz, 1 H), 2.42 (dd, *J* = 16.9, 8.0 Hz, 1 H), 2.23 (s, 3 H); ^13^C NMR (100 MHz, Chloroform-d) δ 192.9, 171.9, 159.9, 136.8, 127.1, 124.9, 120.9, 120.0, 70.0, 52.0, 42.3, 30.1, 15.5; HRMS (ESI): *m*/*z* [M + H]^+^ calcd for C_13_H_15_O_4_: 235.0965; found: 235.0967.

methyl 2-(6-methyl-4-oxochroman-3-yl)acetate (**3ca**):

^1^H NMR (400 MHz, Chloroform-d) δ 7.66 (s, 1 H), 7.29 (d, *J* = 8.4 Hz, 1 H), 6.87 (d, *J* = 8.4 Hz, 1 H), 4.56 (dd, *J* = 11.1, 5.2 Hz, 1 H), 4.25 (t, *J* = 11.5 Hz, 1 H), 3.72 (s, 3 H), 3.31 (dt, *J* = 12.4, 6.4 Hz, 1 H), 2.92 (dd, *J* = 17.0, 4.8 Hz, 1 H), 2.42 (dd, *J* = 17.0, 8.1 Hz, 1 H), 2.30 (s, 3 H); ^13^C NMR (100 MHz, Chloroform-d) δ 192.8, 171.9, 159.8, 137.1, 131.0, 126.9, 120.0, 117.6, 70.2, 52.0, 42.5, 30.1, 20.4; HRMS (ESI): *m*/*z* [M + H]^+^ calcd for C_13_H_15_O_4_: 235.0965; found: 235.0968.

methyl 2-(7-methoxy-4-oxochroman-3-yl)acetate (**3da**):

^1^H NMR (400 MHz, Chloroform-d) δ 7.81 (d, *J* = 8.8 Hz, 1 H), 6.58 (dd, *J* = 8.8, 2.3 Hz, 1 H), 6.40 (d, *J* = 2.2 Hz, 1 H), 4.57 (dd, *J* = 11.1, 5.2 Hz, 1 H), 4.27 (t, *J* = 11.4 Hz, 1 H), 3.83 (s, 3 H), 3.72 (s, 3 H), 3.37–3.20 (m, 1 H), 2.94 (dd, *J* = 17.0, 4.8 Hz, 1 H), 2.40 (dd, *J* = 17.0, 8.3 Hz, 1 H); ^13^C NMR (100 MHz, Chloroform-d) δ 191.2, 172.0, 166.0, 163.7, 129.1, 114.2, 110.1, 100.6, 70.5, 55.6, 52.0, 42.0, 30.1; HRMS (ESI): *m*/*z* [M + H]^+^ calcd for C_13_H_15_O_5_: 251.0914; found: 251.0917.

methyl 2-(8-(tert-butyl)-4-oxochroman-3-yl)acetate (**3ea**):

^1^H NMR (400 MHz, Chloroform-d) δ 7.80 (d, *J* = 7.8 Hz, 1 H), 7.48 (d, *J* = 7.6 Hz, 1 H), 6.96 (t, *J* = 7.7 Hz, 1 H), 4.66 (dd, *J* = 11.0, 5.2 Hz, 1 H), 4.27 (t, *J* = 11.6 Hz, 1 H), 3.74 (s, 3 H), 3.33 (td, *J* = 12.9, 5.1 Hz, 1 H), 2.96 (dd, *J* = 17.0, 4.8 Hz, 1 H), 2.43 (dd, *J* = 17.0, 8.2 Hz, 1 H), 1.38 (s, 9 H); ^13^C NMR (100 MHz, Chloroform-d) δ 193.2, 172.0, 160.9, 138.9, 133.0, 125.5, 121.2, 121.0, 69.7, 52.0, 42.4, 34.9, 30.2, 29.6; HRMS (ESI): *m*/*z* [M + H]^+^ calcd for C_16_H_21_O_4_: 277.1434; found: 277.1432.

methyl 2-(7-fluoro-4-oxochroman-3-yl)acetate (**3fa**):

^1^H NMR (400 MHz, Chloroform-d) δ 7.90 (dd, *J* = 8.8, 6.6 Hz, 1 H), 6.74 (td, *J* = 8.5, 2.3 Hz, 1 H), 6.66 (dd, *J* = 9.8, 2.3 Hz, 1 H), 4.61 (dd, *J* = 11.2, 5.3 Hz, 1 H), 4.31 (t, *J* = 11.7 Hz, 1 H), 3.73 (s, 3 H), 3.43–3.24 (m, 1 H), 2.93 (dd, *J* = 17.1, 4.9 Hz, 1 H), 2.43 (dd, *J* = 17.1, 8.0 Hz, 1 H); ^13^C NMR (100 MHz, Chloroform-d) δ 191.2, 171.7, 167.4 (d, *J*_C-F_ = 254.0 Hz), 163.3 (d, *J*_C-F_ = 14.0 Hz), 129.9 (d, *J*_C-F_ = 12.0 Hz), 117.4, 110.0 (d, *J*_C-F_ = 22.0 Hz), 104.6 (d, *J*_C-F_ = 24.0 Hz), 104.5, 70.6, 52.1, 42.1, 29.9; ^19^F NMR (376 MHz, Chloroform-d) δ −100.27; HRMS (ESI): *m*/*z* [M + H]^+^ calcd for C_12_H_12_FO_4_: 239.0714; found: 239.0710.

methyl 2-(6-fluoro-4-oxochroman-3-yl)acetate (**3ga**):

^1^H NMR (400 MHz, Chloroform-d) δ 7.52 (dd, *J* = 8.2, 3.1 Hz, 1 H), 7.20 (td, *J* = 8.4, 3.2 Hz, 1 H), 6.95 (dd, *J* = 9.1, 4.2 Hz, 1 H), 4.58 (dd, *J* = 11.2, 5.3 Hz, 1 H), 4.28 (t, *J* = 11.7 Hz, 1 H), 3.72 (s, 3 H), 3.31 (td, *J* = 12.7, 5.1 Hz, 1 H), 2.91 (dd, *J* = 17.1, 4.8 Hz, 1 H), 2.44 (dd, *J* = 17.1, 8.0 Hz, 1 H); ^13^C NMR (100 MHz, Chloroform-d) δ 191.9, 171.6, 158.0 (d, *J*_C–F_ = 1Hz), 157.2 (d, *J*_C–F_ = 241.0 Hz), 123.5 (d, *J*_C–F_ = 25.0 Hz), 120.8 (d, *J*_C–F_ = 6.0 Hz), 119.5 (d, *J*_C–F_ = 7.0 Hz), 112.2 (d, *J*_C–F_ =23.0 Hz), 70.3, 52.1, 42.3, 29.9; ^19^F NMR (376 MHz, Chloroform-d) δ −121.24; HRMS (ESI): *m*/*z* [M + H]^+^ calcd for C_12_H_12_FO_4_: 239.0714; found: 239.0715.

methyl 2-(8-chloro-4-oxochroman-3-yl)acetate (**3ha**):

^1^H NMR (400 MHz, Chloroform-d) δ 7.80 (dd, *J* = 7.9, 1.3 Hz, 1 H), 7.57 (dd, *J* = 7.8, 1.3 Hz, 1 H), 6.98 (t, *J* = 7.8 Hz, 1 H), 4.74 (dd, *J* = 11.2, 5.3 Hz, 1 H), 4.40 (t, *J* = 11.8 Hz, 1 H), 3.73 (s, 3 H), 3.44–3.31 (m, 1 H), 2.92 (dd, *J* = 17.1, 4.9 Hz, 1 H), 2.48 (dd, *J* = 17.1, 7.8 Hz, 1 H); ^13^C NMR (100 MHz, Chloroform-d) δ 191.8, 171.5, 157.1, 136.1, 126.0, 122.5, 121.7, 121.6, 70.7, 52.1, 42.1, 29.9; HRMS (ESI): *m*/*z* [M + H]^+^ calcd for C_12_H_12_ClO_4_: 255.0419; found: 255.0424.

methyl 2-(7-chloro-4-oxochroman-3-yl)acetate (**3ia**):

^1^H NMR (400 MHz, Chloroform-d) δ 7.81 (d, *J* = 8.9 Hz, 1 H), 7.00 (dd, *J* = 4.4, 2.6 Hz, 2 H), 4.61 (dd, *J* = 11.2, 5.3 Hz, 1 H), 4.31 (t, *J* = 11.7 Hz, 1 H), 3.73 (s, 3 H), 3.32 (td, *J* = 12.8, 5.1 Hz, 1 H), 2.92 (dd, *J* = 17.1, 4.8 Hz, 1 H), 2.44 (dd, *J* = 17.1, 8.0 Hz, 1 H); ^13^C NMR (100 MHz, Chloroform-d) δ 191.5, 171.6, 162.0, 141.9, 128.6, 122.4, 119.0, 118.0, 70.5, 52.1, 42.3, 29.9; HRMS (ESI): *m*/*z* [M + H]^+^ calcd for C_12_H_12_ClO_4_: 255.0419; found: 255.0422.

methyl 2-(5-chloro-4-oxochroman-3-yl)acetate (**3ja**):

^1^H NMR (400 MHz, Chloroform-d) δ 7.33 (t, *J* = 8.1 Hz, 1 H), 7.04 (d, *J* = 7.8 Hz, 1 H), 6.90 (d, *J* = 8.4 Hz, 1 H), 4.58 (dd, *J* = 11.2, 5.3 Hz, 1 H), 4.29 (t, *J* = 11.7 Hz, 1 H), 3.73 (s, 3 H), 3.36 (td, *J* = 12.8, 5.3 Hz, 1 H), 2.92 (dd, *J* = 17.0, 5.2 Hz, 1 H), 2.41 (dd, *J* = 17.0, 7.7 Hz, 1 H); ^13^C NMR (100 MHz, Chloroform-d) δ 190.7, 171.7, 163.0, 134.8, 134.5, 124.7, 117.7, 116.9, 69.7, 52.1, 43.0, 30.0; HRMS (ESI): *m*/*z* [M + H]^+^ calcd for C_12_H_12_ClO_4_: 255.0419; found: 255.0426.

methyl 2-(7-bromo-4-oxochroman-3-yl)acetate (**3ka**):

^1^H NMR (400 MHz, Chloroform-d) δ 7.67 (d, *J* = 8.3 Hz, 1 H), 7.10 (dd, *J* = 10.8, 2.4 Hz, 2 H), 4.54 (dd, *J* = 11.2, 5.3 Hz, 1 H), 4.24 (t, *J* = 11.7 Hz, 1 H), 3.66 (s, 3 H), 3.25 (td, *J* = 12.8, 5.1 Hz, 1 H), 2.86 (dd, *J* = 17.1, 4.8 Hz, 1 H), 2.37 (dd, *J* = 17.1, 8.0 Hz, 1 H); ^13^C NMR (100 MHz, Chloroform-d) δ 191.7, 171.6, 161.9, 130.5, 128.6, 125.2, 121.0, 119.3, 70.5, 52.1, 42.3, 29.9; HRMS (ESI): *m*/*z* [M + H]^+^ calcd for C_12_H_12_BrO_4_: 298.9913; found: 298.9909.

methyl 2-(6-bromo-4-oxochroman-3-yl)acetate (**3la**):

^1^H NMR (400 MHz, Chloroform-d) δ 7.97 (d, *J* = 2.4 Hz, 1 H), 7.54 (dd, *J* = 8.8, 2.4 Hz, 1 H), 6.88 (d, *J* = 8.8 Hz, 1 H), 4.60 (dd, *J* = 11.2, 5.3 Hz, 1 H), 4.29 (t, *J* = 11.7 Hz, 1 H), 3.72 (s, 3 H), 3.31 (td, *J* = 12.7, 5.1 Hz, 1 H), 2.92 (dd, *J* = 17.1, 4.8 Hz, 1 H), 2.44 (dd, *J* = 17.1, 8.0 Hz, 1 H); ^13^C NMR (100 MHz, Chloroform-d) δ 191.3, 171.6, 160.6, 138.6, 129.7, 121.7, 119.9, 114.2, 70.2, 52.1, 42.2, 29.9; HRMS (ESI): *m*/*z* [M + H]^+^ calcd for C_12_H_12_BrO_4_: 298.9913; found: 298.9915.

methyl 3-(2-methoxy-2-oxoethyl)-4-oxochromane-6-carboxylate (**3ma**):

^1^H NMR (400 MHz, Chloroform-d) δ 8.56 (d, *J* = 2.1 Hz, 1 H), 8.13 (dd, *J* = 8.7, 2.1 Hz, 1 H), 7.02 (d, *J* = 8.7 Hz, 1 H), 4.67 (dd, *J* = 11.3, 5.4 Hz, 1 H), 4.35 (t, J = 11.8 Hz, 1 H), 3.89 (s, 3 H), 3.73 (s, 3 H), 3.36 (dt, *J* = 12.4, 6.3 Hz, 1 H), 2.96 (dd, *J* = 17.1, 4.7 Hz, 1 H), 2.45 (dd, *J* = 17.1, 8.0 Hz, 1 H); ^13^C NMR (100 MHz, Chloroform-d) δ 191.5, 171.6, 165.9, 164.7, 136.7, 129.8, 123.8, 120.0, 118.2, 70.4, 52.1, 42.3, 29.9; HRMS (ESI): *m*/*z* [M + H]^+^ calcd for C_14_H_15_O_6_: 279.0863; found: 279.0869.

methyl 2-(6-nitro-4-oxochroman-3-yl)acetate (**3na**):

^1^H NMR (400 MHz, Chloroform-d) δ 8.75 (d, *J* = 2.6 Hz, 1 H), 8.32 (dd, *J* = 9.1, 2.6 Hz, 1 H), 7.11 (d, *J* = 9.1 Hz, 1 H), 4.75 (dd, *J* = 11.3, 5.5 Hz, 1 H), 4.43 (t, *J* = 12.0 Hz, 1 H), 3.73 (s, 3 H), 3.39 (td, *J* = 12.6, 5.2 Hz, 1 H), 3.05–2.90 (m, 1 H), 2.51 (dd, *J* = 17.3, 7.7 Hz, 1 H); ^13^C NMR (100 MHz, Chloroform-d) δ 190.5, 171.3, 165.6, 142.2, 130.3, 123.9, 120.0, 119.2, 70.6, 52.2, 42.1, 29.6; HRMS (ESI): *m*/*z* [M + H]^+^ calcd for C_12_H_12_NO_6_: 266.0659; found: 266.0664.

methyl 2-(7-(benzyloxy)-4-oxochroman-3-yl)acetate (**3oa**):

^1^H NMR (400 MHz, Chloroform-d) δ 7.83 (d, *J* = 8.8 Hz, 1 H), 7.38 (dd, *J* = 14.3, 5.8 Hz, 5 H), 6.66 (d, *J* = 8.8 Hz, 1 H), 6.48 (s, 1 H), 5.08 (s, 2 H), 4.57 (dd, *J* = 11.1, 5.2 Hz, 1 H), 4.26 (t, *J* = 11.5 Hz, 1 H), 3.72 (s, 3 H), 3.27 (dq, *J* = 12.4, 5.0 Hz, 1 H), 2.93 (dd, *J* = 17.0, 4.7 Hz, 1 H), 2.40 (dd, *J* = 17.0, 8.3 Hz, 1 H); ^13^C NMR (100 MHz, Chloroform-d) δ 191.1, 172.0, 165.1, 163.6, 135.8, 129.1, 128.7, 128.3, 127.5, 114.4, 110.6, 101.6, 70.5, 70.3, 52.0, 42.0, 30.1; HRMS (ESI): *m*/*z* [M + H]^+^ calcd for C_19_H_19_O_5_: 327.1227; found: 327.1236.

methyl 2-(6,8-dichloro-4-oxochroman-3-yl)acetate (**3pa**):

^1^H NMR (400 MHz, Chloroform-d) δ 7.76 (d, *J* = 2.4 Hz, 1 H), 7.55 (d, *J* = 2.4 Hz, 1 H), 4.74 (dd, *J* = 11.3, 5.4 Hz, 1 H), 4.39 (t, *J* = 11.8 Hz, 1 H), 3.73 (s, 3 H), 3.35 (td, *J* = 12.6, 5.1 Hz, 1 H), 2.91 (dd, *J* = 17.2, 4.7 Hz, 1 H), 2.50 (dd, *J* = 17.3, 7.7 Hz, 1 H); ^13^C NMR (100 MHz, Chloroform-d) δ 190.7, 171.3, 155.9, 135.6, 126.8, 125.4, 123.7, 122.0, 70.8, 52.2, 42.0, 29.8; HRMS (ESI): *m*/*z* [M + H]^+^ calcd for C_12_H_11_Cl_2_O_4_: 289.0029; found: 289.0031.

methyl 2-(4-oxo-3,4-dihydro-2H-benzo[h]chromen-3-yl)acetate (**3qa**):

^1^H NMR (400 MHz, Chloroform-d) δ 9.42 (d, *J* = 8.7 Hz, 1 H), 7.92 (d, *J* = 9.1 Hz, 1 H), 7.75 (d, *J* = 8.2 Hz, 1 H), 7.62 (t, *J* = 7.8 Hz, 1 H), 7.43 (t, *J* = 7.5 Hz, 1 H), 7.10 (d, *J* = 9.0 Hz, 1 H), 4.68 (dd, *J* = 11.1, 5.2 Hz, 1 H), 4.41 (t, *J* = 11.5 Hz, 1 H), 3.75 (s, 3 H), 3.43 (dq, *J* = 12.7, 5.2 Hz, 1 H), 2.99 (dd, *J* = 16.8, 5.1 Hz, 1 H), 2.48 (dd, *J* = 16.8, 8.0 Hz, 1 H); ^13^C NMR (100 MHz, Chloroform-d) δ 193.5, 172.1, 163.7, 137.6, 131.6, 129.7, 129.2, 128.4, 125.8, 124.9, 118.6, 112.0, 70.1, 52.0, 43.0, 30.5; HRMS (ESI): *m*/*z* [M + H]^+^ calcd for C_16_H_15_O_4_: 271.0965; found: 271.0959.

methyl 2-(3-methyl-4-oxochroman-3-yl)acetate (**3ra**):

^1^H NMR (400 MHz, Chloroform-d) δ 7.90 (d, *J* = 7.8 Hz, 1 H), 7.47 (t, *J* = 7.7 Hz, 1 H), 7.11–6.85 (m, 2 H), 4.63 (d, *J* = 11.4 Hz, 1 H), 4.23 (d, *J* = 11.4 Hz, 1 H), 3.66 (s, 3 H), 2.81 (d, *J* = 16.0 Hz, 1 H), 2.52 (d, *J* = 16.0 Hz, 1 H), 1.29 (s, 3 H); ^13^C NMR (100 MHz, Chloroform-d) δ 195.3, 171.1, 161.0, 135.8, 127.9, 121.7, 119.4, 117.7, 74.2, 51.7, 43.7, 37.6, 19.1; HRMS (ESI): *m*/*z* [M + H]^+^ calcd for C_13_H_15_O_4_: 235.0965; found: 235.0969.

methyl 2-(1-oxo-2,3-dihydro-1H-inden-2-yl)acetate (**3sa**):

^1^H NMR (400 MHz, Chloroform-d) δ 7.77 (d, *J* = 7.6 Hz, 1 H), 7.60 (t, *J* = 7.4 Hz, 1 H), 7.46 (d, *J* = 7.6 Hz, 1 H), 7.38 (t, *J* = 7.5 Hz, 1 H), 3.69 (s, 3 H), 3.47 (dd, *J* = 17.1, 8.0 Hz, 1 H), 3.06–2.96 (m, 2 H), 2.89 (dd, *J* = 17.1, 4.2 Hz, 1 H), 2.67–2.57 (m, 1 H); ^13^C NMR (100 MHz, Chloroform-d) δ 206.7, 172.5, 153.2, 136.3, 134.9, 127.5, 126.5, 124.0, 51.9, 43.6, 35.0, 33.0; HRMS (ESI): *m*/*z* [M + H]^+^ calcd for C_12_H_13_O_3_: 205.0859; found: 205.0862.

ethyl 2-(4-oxochroman-3-yl)acetate (**3ab**):

^1^H NMR (400 MHz, Chloroform-d) δ 7.88 (d, *J* = 6.9 Hz, 1 H), 7.47 (t, *J* = 7.8 Hz, 1 H), 7.06–6.88 (m, 2 H), 4.59 (dd, *J* = 11.2, 5.3 Hz, 1 H), 4.29 (t, *J* = 11.6 Hz, 1 H), 4.18 (q, *J* = 8.5, 6.9 Hz, 2 H), 3.33 (td, *J* = 12.9, 5.1 Hz, 1 H), 2.92 (dd, *J* = 17.0, 4.8 Hz, 1 H), 2.41 (dd, *J* = 17.0, 8.1 Hz, 1 H), 1.28 (t, *J* = 7.1 Hz, 3 H); ^13^C NMR (100 MHz, Chloroform-d) δ 192.6, 171.3, 161.7, 136.0, 127.3, 121.5, 120.4, 117.8, 70.2, 61.0, 42.5, 30.3, 14.1; HRMS (ESI): *m*/*z* [M + H]^+^ calcd for C_13_H_15_O_4_: 235.0965; found: 235.0967.

butyl 2-(4-oxochroman-3-yl)acetate (**3ac**):

^1^H NMR (400 MHz, Chloroform-d) δ 7.89 (d, *J* = 7.8 Hz, 1 H), 7.47 (t, *J* = 7.7 Hz, 1 H), 7.11–6.86 (m, 2 H), 4.60 (dd, *J* = 11.2, 5.2 Hz, 1 H), 4.29 (t, *J* = 11.6 Hz, 1 H), 4.13 (tq, *J* = 7.0, 4.1 Hz, 2 H), 3.32 (td, *J* = 12.9, 5.0 Hz, 1 H), 2.94 (dd, *J* = 16.9, 4.7 Hz, 1 H), 2.42 (dd, *J* = 16.9, 8.2 Hz, 1 H), 1.63 (t, *J* = 7.3 Hz, 2 H), 1.39 (dq, *J* = 14.7, 7.4 Hz, 2 H), 0.94 (t, *J* = 7.4 Hz, 3 H); ^13^C NMR (100 MHz, Chloroform-d) δ 192.6, 171.4, 161.7, 136.0, 127.4, 121.5, 120.5, 117.8, 70.2, 64.9, 42.5, 30.6, 30.4, 19.1, 13.7; HRMS (ESI): *m*/*z* [M + H]^+^ calcd for C_15_H_19_O_4_: 263.1278; found: 263.1273.

hexyl 2-(4-oxochroman-3-yl)acetate (**3ad**):

^1^H NMR (400 MHz, Chloroform-d) δ 7.88 (d, *J* = 7.8 Hz, 1 H), 7.48 (t, *J* = 8.2 Hz, 1 H), 7.13–6.83 (m, 2 H), 4.60 (dd, *J* = 11.2, 5.3 Hz, 1 H), 4.29 (t, *J* = 11.6 Hz, 1 H), 4.11 (t, *J* = 8.1 Hz, 2 H), 3.33 (td, *J* = 12.9, 5.0 Hz, 1 H), 2.94 (dd, *J* = 17.0, 4.7 Hz, 1 H), 2.42 (dd, *J* = 17.0, 8.2 Hz, 1 H), 1.62 (q, *J* = 7.0 Hz, 2 H), 1.32 (d, *J* = 16.3 Hz, 6 H), 0.88 (t, *J* = 6.6 Hz, 3 H); ^13^C NMR (100 MHz, Chloroform-d) δ 192.6, 171.4, 161.7, 136.0, 127.4, 121.5, 120.5, 117.8, 70.2, 65.2, 42.5, 31.4, 30.3, 28.5, 25.5, 22.5, 14.0; HRMS (ESI): *m*/*z* [M + H]^+^ calcd for C_17_H_23_O_4_: 291.1591; found: 291.1592.

isobutyl 2-(4-oxochroman-3-yl)acetate (**3ae**):

^1^H NMR (400 MHz, Chloroform-d) δ 7.88 (d, *J* = 7.8 Hz, 1 H), 7.47 (t, *J* = 7.7 Hz, 1 H), 7.09–6.90 (m, 2 H), 4.60 (dd, *J* = 11.2, 5.2 Hz, 1 H), 4.30 (t, *J* = 11.5 Hz, 1 H), 3.92 (dq, *J* = 9.1, 5.3, 3.7 Hz, 2 H), 3.33 (td, *J* = 12.7, 5.0 Hz, 1 H), 2.95 (dd, *J* = 16.9, 4.7 Hz, 1 H), 2.43 (dd, J = 16.9, 8.2 Hz, 1 H), 1.99–1.88 (m, 1 H), 0.94 (d, *J* = 6.7 Hz, 6 H); ^13^C NMR (100 MHz, Chloroform-d) δ 192.5, 171.4, 161.7, 136.0, 127.4, 121.5, 120.5, 117.8, 71.1, 70.2, 42.5, 30.3, 27.7, 19.0; HRMS (ESI): *m*/*z* [M + H]^+^ calcd for C_15_H_19_O_4_: 263.1278; found: 263.1274.

3-methylpentyl 2-(4-oxochroman-3-yl)acetate (**3af**):

^1^H NMR (400 MHz, Chloroform-d) δ 7.89 (dd, *J* = 7.9, 1.7 Hz, 1 H), 7.55–7.43 (m, 1 H), 7.09–6.92 (m, 2 H), 4.60 (dd, *J* = 11.2, 5.2 Hz, 1 H), 4.30 (t, *J* = 11.6 Hz, 1 H), 4.15 (td, J = 11.8, 10.7, 5.2 Hz, 2 H), 3.33 (td, *J* = 12.8, 5.0 Hz, 1 H), 2.94 (dd, *J* = 16.9, 4.7 Hz, 1 H), 2.42 (dd, *J* = 17.0, 8.2 Hz, 1 H), 1.68 (q, *J* = 7.8 Hz, 1 H), 1.49–1.33 (m, 3 H), 1.19 (dt, *J* = 13.8, 7.1 Hz, 1 H), 0.92–0.85 (m, 6 H); ^13^C NMR (1010 MHz, Chloroform-d) δ 192.6, 171.4, 161.7, 136.0, 127.4, 121.5, 120.5, 117.8, 70.2, 63.7, 42.5, 35.0, 31.4, 30.4, 29.4, 19.0, 11.2; HRMS (ESI): *m*/*z* [M + H]^+^ calcd for C_17_H_23_O_4_: 291.1591; found: 291.1596.

3-phenylpropyl 2-(4-oxochroman-3-yl)acetate (**3ag**):

^1^H NMR (400 MHz, Chloroform-d) δ 7.90 (d, *J* = 7.8 Hz, 1 H), 7.48 (t, *J* = 7.7 Hz, 1 H), 7.33–7.26 (m, 2 H), 7.20 (t, *J* = 6.6 Hz, 3 H), 7.03 (t, *J* = 7.5 Hz, 1 H), 6.98 (d, *J* = 8.4 Hz, 1 H), 4.60 (dd, *J* = 11.2, 5.3 Hz, 1 H), 4.30 (t, *J* = 11.6 Hz, 1 H), 4.15 (tt, *J* = 7.2, 3.7 Hz, 2 H), 3.39–3.26 (m, 1 H), 2.93 (dd, *J* = 16.9, 4.9 Hz, 1 H), 2.70 (t, *J* = 7.6 Hz, 2 H), 2.43 (dd, *J* = 16.9, 8.0 Hz, 1 H), 1.99 (dt, *J* = 13.6, 6.8 Hz, 2 H); ^13^C NMR (100 MHz, Chloroform-d) δ 192.5, 171.3, 161.7, 141.0, 136.0, 128.4, 128.4, 127.3, 126.0, 121.5, 120.5, 117.8, 70.2, 64.3, 42.5, 32.1, 30.3, 30.1; HRMS (ESI): *m*/*z* [M + H]^+^ calcd for C_20_H_21_O_4_: 325.1434; found: 325.1437.

2-fluoroethyl 2-(4-oxochroman-3-yl)acetate (**3ah**):

^1^H NMR (400 MHz, Chloroform-d) δ 7.88 (d, *J* = 7.8 Hz, 1 H), 7.48 (t, *J* = 7.7 Hz, 1 H), 7.11–6.84 (m, 2 H), 4.74–4.50 (m, 3 H), 4.47–4.21 (m, 3 H), 3.36 (dq, *J* = 12.3, 5.5 Hz, 1 H), 2.98 (dd, *J* = 17.0, 5.0 Hz, 1 H), 2.49 (dd, *J* = 17.0, 7.8 Hz, 1 H); ^13^C NMR (100 MHz, Chloroform-d) δ 192.4, 171.2, 161.7, 136.1, 127.4, 121.5, 120.4, 117.8, 81.2 (d, *J*_C-F_ = 170.0 Hz), 70.1, 63.7 (d, *J*_C-F_ = 20.0 Hz), 42.4, 30.2; ^19^F NMR (376 MHz, Chloroform-d) δ −216.5; HRMS (ESI): *m*/*z* [M + H]^+^ calcd for C_13_H_14_FO_4_: 253.0871; found: 253.0873.

2-ethoxyethyl 2-(4-oxochroman-3-yl)acetate (**3ai**)

^1^H NMR (400 MHz, Chloroform-d) δ 7.89 (d, *J* = 7.8 Hz, 1 H), 7.48 (t, *J* = 7.7 Hz, 1 H), 7.12–6.91 (m, 2 H), 4.60 (dd, *J* = 11.1, 5.2 Hz, 1 H), 4.43–4.14 (m, 3 H), 3.72–3.58 (m, 2 H), 3.53 (q, *J* = 6.9 Hz, 2 H), 3.34 (dq, *J* = 12.6, 5.3 Hz, 1 H), 2.99 (dd, *J* = 17.0, 4.6 Hz, 1 H), 2.47 (dd, *J* = 17.0, 8.2 Hz, 1 H), 1.21 (t, *J* = 6.9 Hz, 3 H); ^13^C NMR (100 MHz, Chloroform-d) δ 192.5, 171.4, 161.7, 136.0, 127.4, 121.5, 120.5, 117.8, 70.2, 68.2, 66.6, 64.1, 42.5, 30.3, 15.1; HRMS (ESI): *m*/*z* [M + H]^+^ calcd for C_15_H_19_O_5_: 279.1227; found: 279.1223.

4,4,4-trifluorobutyl 2-(4-oxochroman-3-yl)acetate (**3aj**):

^1^H NMR (400 MHz, Chloroform-d) δ 7.88 (dd, *J* = 7.9, 1.6 Hz, 1 H), 7.58–7.41 (m, 1 H), 7.11–6.90 (m, 2 H), 4.59 (dd, *J* = 11.1, 5.3 Hz, 1 H), 4.40–4.25 (m, 2 H), 4.24–4.12 (m, 2 H), 3.34 (dq, *J* = 12.3, 5.4 Hz, 1 H), 2.90 (dd, *J* = 17.0, 5.4 Hz, 1 H), 2.45 (dd, *J* = 16.9, 7.5 Hz, 1 H), 2.20 (ddd, *J* = 10.7, 7.7, 5.2 Hz, 2 H), 2.03 (dq, *J* = 13.1, 6.6 Hz, 1 H), 1.93 (dq, *J* = 13.1, 6.6 Hz, 2 H); ^13^C NMR (100 MHz, Chloroform-d) δ 192.5, 171.2, 161.7, 136.1, 127.3, 126.8 (q, *J*_C-F_ = 274.0 Hz), 121.6, 120.4, 117.8, 70.2, 63.1, 42.5, 30.6 (q, *J*_C-F_ = 29.0 Hz), 30.2, 21.5; ^19^F NMR (376 MHz, Chloroform-d) δ −66.4; HRMS (ESI): *m*/*z* [M + H]^+^ calcd for C_16_H_18_F_3_O_4_: 331.1152; found: 331.1146.

cyclobutyl 2-(4-oxochroman-3-yl)acetate (**3ak**):

^1^H NMR (400 MHz, Chloroform-d) δ 7.88 (d, *J* = 7.8 Hz, 1 H), 7.47 (t, *J* = 7.7 Hz, 1 H), 7.15–6.90 (m, 2 H), 5.06–4.97 (m, 1 H), 4.58 (dd, *J* = 11.1, 5.2 Hz, 1 H), 4.28 (t, *J* = 11.6 Hz, 1 H), 3.31 (td, *J* = 12.6, 5.1 Hz, 1 H), 2.90 (dd, *J* = 16.9, 4.7 Hz, 1 H), 2.38 (dd, *J* = 16.8, 8.1 Hz, 3 H), 2.15–2.00 (m, 2 H), 1.80 (q, *J* = 10.2 Hz, 1 H), 1.63 (dt, *J* = 18.7, 10.8 Hz, 1 H); ^13^C NMR (100 MHz, Chloroform-d) δ 192.5, 170.6, 161.7, 135.9, 127.3, 121.5, 120.4, 117.8, 70.2, 69.3, 42.5, 30.3, 30.2, 30.2, 13.5; HRMS (ESI): *m*/*z* [M + H]^+^ calcd for C_15_H_17_O_4_: 261.1121; found: 261.1125.

cyclohexyl 2-(4-oxochroman-3-yl)acetate (**3al**):

^1^H NMR (400 MHz, Chloroform-d) δ 7.89 (d, *J* = 7.8 Hz, 1 H), 7.48 (t, *J* = 7.8 Hz, 1 H), 7.12–6.88 (m, 2 H), 4.81 (td, *J* = 8.8, 4.1 Hz, 1 H), 4.60 (dd, *J* = 11.1, 5.2 Hz, 1 H), 4.30 (t, *J* = 11.5 Hz, 1 H), 3.32 (td, *J* = 12.7, 12.3, 5.1 Hz, 1 H), 2.92 (dd, *J* = 16.8, 4.7 Hz, 1 H), 2.40 (dd, *J* = 16.8, 8.3 Hz, 1 H), 1.98–1.68 (m, 4 H), 1.54–1.27 (m, 6 H); ^13^C NMR (100 MHz, Chloroform-d) δ 192.6, 170.7, 161.7, 136.0, 127.4, 121.5, 120.5, 117.8, 73.4, 70.3, 42.6, 31.6, 30.7, 25.3, 23.7; HRMS (ESI): *m*/*z* [M + H]^+^ calcd for C_17_H_21_O_4_: 289.1434; found: 289.1439.

3-neopentylchroman-4-one (**3am**):

^1^H NMR (400 MHz, Chloroform-d) δ 7.89 (d, *J* = 7.8 Hz, 1 H), 7.46 (t, *J* = 7.7 Hz, 1 H), 7.01 (t, *J* = 7.5 Hz, 1 H), 6.95 (d, *J* = 8.3 Hz, 1 H), 4.50 (dd, *J* = 11.3, 5.0 Hz, 1 H), 4.18 (t, *J* = 11.1 Hz, 1 H), 2.72 (dq, *J* = 10.3, 4.8 Hz, 1 H), 2.06 (dd, J = 14.3, 3.6 Hz, 1 H), 1.05 (dd, J = 14.3, 5.7 Hz, 1 H), 0.97 (s, 9 H); ^13^C NMR (100 MHz, Chloroform-d) δ 194.7, 161.4, 135.5, 127.5, 121.2, 120.8, 117.6, 71.9, 42.8, 38.3, 30.7, 29.4; HRMS (ESI): *m*/*z* [M + H]^+^ calcd for C_14_H_19_O_2_: 219.1380; found: 219.1381.

## 4. Conclusions

In conclusion, we have developed a (NH_4_)_2_S_2_O_8_ enabled alkoxycarbonyl triggered cascade radical annulation of 2-(allyloxy)arylaldehydes with oxalates under metal-free conditions. The present developed methodology has the advantages of operational simplicity, readily available substrates, broad substrate scope and good functional group tolerance, thus providing a convenient and practical approach for the synthesis of ester-containing chroman-4-ones.

## Figures and Tables

**Table 1:** Optimization of reaction conditions ^a^.

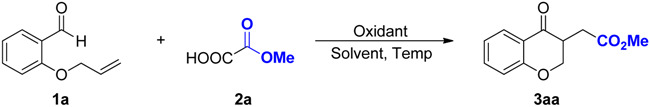				
Entry	Oxidant	Solvent	Temp (°C)	Yield of 3aa ^b^
1	(NH_4_)_2_S_2_O_8_	DMSO	80	71%
2	(NH_4_)_2_S_2_O_8_	DMF	80	0%
3	(NH_4_)_2_S_2_O_8_	CH_3_CN	80	0%
4	(NH_4_)_2_S_2_O_8_	DCE	80	0%
5	(NH_4_)_2_S_2_O_8_	THF	80	0%
6	(NH_4_)_2_S_2_O_8_	H_2_O	80	0%
7	(NH_4_)_2_S_2_O_8_	DMSO/H_2_O (500:1)	80	76%
8	(NH_4_)_2_S_2_O_8_	DMSO/H_2_O (100:1)	80	71%
9	(NH_4_)_2_S_2_O_8_	DMSO/H_2_O (10:1)	80	44%
10	(NH_4_)_2_S_2_O_8_	DMSO/H_2_O (5:1)	80	17%
11	K_2_S_2_O_8_	DMSO/H_2_O (500:1)	80	0%
12	Na_2_S_2_O_8_	DMSO/H_2_O (500:1)	80	0%
13	TBHP	DMSO/H_2_O (500:1)	80	0%
14	Selectfluor	DMSO/H_2_O (500:1)	80	0%
15	PhI(OAc)_2_	DMSO/H_2_O (500:1)	80	0%
16	(NH_4_)_2_S_2_O_8_	DMSO/H_2_O (500:1)	90	81%
17	(NH_4_)_2_S_2_O_8_	DMSO/H_2_O (500:1)	100	79%
18	(NH_4_)_2_S_2_O_8_	DMSO/H_2_O (500:1)	70	34%
19 ^c^	(NH_4_)_2_S_2_O_8_	DMSO/H_2_O (500:1)	90	57%
20 ^d^	(NH_4_)_2_S_2_O_8_	DMSO/H_2_O (500:1)	90	64%
21		DMSO/H_2_O (500:1)	90	0%

^a^ General conditions, unless otherwise noted: **1a** (0.1mmol, 1 equiv.), **2a** (0.3 mmol, 3 equiv.), oxidant (0.3mmol, 3 equiv.), solvent (0.6 mL), under N_2_ atmosphere for 24 h. ^b^ Isolated yields. ^c^ 2 equiv. of (NH_4_)_2_S_2_O_8_ was used. ^d^ 2 equiv. of **2a** was used.

## Data Availability

The data presented in this study are available on request from the corresponding author.

## Supplementary Materials

The following supporting information can be downloaded at: https://www.mdpi.com/article/10.3390/ijms24055028/s1. Refs. [47,48,49,50,51] are cited in the Supplementary Materials.
