# Supplementary material for: Synthesis of Ester-Containing Chroman-4-Ones via Cascade Radical Annulation of 2-(Allyloxy)Arylaldehydes with Oxalates under Metal Free Conditions
Source: Int J Mol Sci. 2023 Mar 6;24(5):5028. doi: 10.3390/ijms24055028 (PMC10003100; doi:10.3390/ijms24055028)
Supplement: Supplementary file 1 [file ijms-24-05028-s001.zip › ijms-2161025-supplementary.pdf]

Synthesis of Ester-containing Chroman-4-ones via Cascade Radical Annulation of 2-(allyloxy)arylaldehydes with Oxalates under Metal Free Conditions

Sha Peng, Jiao Liu, Ze-Qun Jiang, Lin Yuan, Xiao-Wen Liu * and Long-Yong Xie *

College of Chemistry and Bioengineering, Hunan University of Science and Engineering, Yongzhou 425100, China

E-mail: xielongyong@huse.edu.cn

Table of Content

1. General information **S2**

2. Experimental Section **S2**

3. Characterization data of products **S9**

4. ^{1}H and ^{13}C NMR spectra of products **S18**

1. General information

Unless otherwise specified, all chemicals were purchased from commercial suppliers and directly used as received without additional purification. Column chromatography was carried out with silica gel (200-300 mesh) to purify products using proper solvents as the eluent system. NMR spectra were recorded at 400 MHz for ^{1}H NMR spectra and 100 MHz for ^{13}C NMR spectra by using a German Bruker Avance 400 spectrometer. Chemical shifts are quoted in parts per million referenced to the appropriate solvent peak (^{1}H NMR: $CDCl_3$ 7.26 ppm, ^{13}C NMR: $CDCl_3$ 77.0 ppm). The following abbreviations were used to describe peak splitting patterns when appropriate: s = singlet, d = doublet, t = triplet, q = quartet, m = multiplet. Mass spectra were performed on a spectrometer operating on ESI-TOF.

2. Experimental Section

General procedure for the preparation of ester-containing chroman-4-ones 3

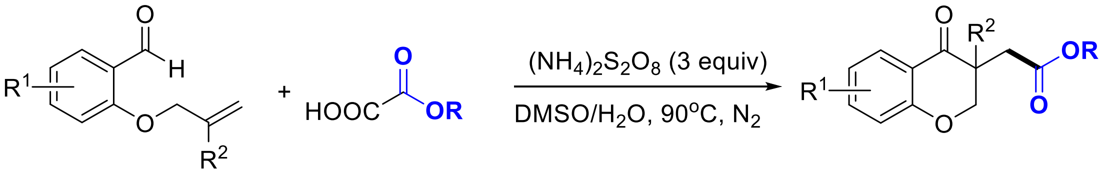

1 **2** **3**

An oven-dried 10mL reaction tube was charged with 2-(allyloxy)arylaldehyde **1** (0.3 mmol, 1eq), oxalates **2** (0.9 mmol, 3eq) and $(NH_4)_2S_2O_8$ (0.9 mmol, 3eq) in a DMSO aqueous solution (1.8 mL, V_{DMSO}/V_{H2O} = 500/1) with a magnetic stirring bar. The mixture was then stirred at 90°C under N_2 atmosphere conditions for about 24h. The reaction was monitored by TLC. After completion, water (10mL) was added and the mixture was extracted with EtOAc (10 mL× 3), the solvent was then removed under vacuum. The residue was purified by flash column chromatography using a mixture of petroleum ether and ethyl acetate as eluent to give the desired products **3**.

Gram-scale synthesis of 3aa

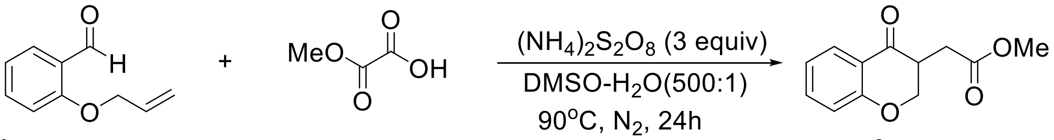

1a, 6 mmol, 0.972 g **3aa**, 1.016 g

An oven-dried 100 mL round-bottom flask was charged with 2-(allyloxy)benzaldehyde **1a** (0.972 g, 6 mmol), 2-methoxy-2-oxoacetic acid **2a** (1.872 g, 18 mmol) and $(NH_4)_2S_2O_8$ (4.104 g, 18 mmol) in a DMSO aqueous solution (36 mL, V_{DMSO}/V_{H2O} = 500/1) with a magnetic stirring bar. The mixture was then stirred at 90°C under N_2 atmosphere conditions for about 24h. The reaction was monitored by TLC. After completion, water (30 mL) was added and the mixture was extracted with EtOAc (40 mL× 3), the solvent was then removed under vacuum. The residue was purified by flash column chromatography using a mixture of petroleum ether and ethyl acetate as eluent to give 1.016 gram of **3aa**, yielding 77%.

Preparation of product 4a *via* the hydrolysis of 3aa

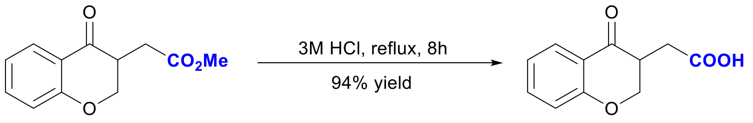

Methyl 2-(4-oxochroman-3-yl)acetate **3aa** (0.5 mmol) was added to a solution of 3M HCl solution (5mL). The reaction mixture was stirred at 100°C under N_2 atmosphere for about 6h. After completion, the reaction was allowed to cool to room temperature and extracted with CH_2Cl_2 (3 x 5 mL), the organic layer was dried over anhydrous $MgSO_4$ and then concentrated under vacuo to give analytically pure product **4a.** ^{1}H NMR (400 MHz, DMSO-d_6) δ 7.75 (d, *J* = 7.8 Hz, 1 H), 7.56 (t, *J* = 8.3 Hz, 1 H), 7.14 – 7.00 (m, 2 H), 4.59 (dd, *J* = 11.1, 5.5 Hz, 1 H), 4.43 – 4.30 (m, 1 H), 3.25 (dq, *J* = 12.2, 5.7 Hz, 1 H), 2.68 (dd, *J* = 17.1, 5.3 Hz, 1 H), 2.44 (dd, *J* = 17.1, 6.8 Hz, 1 H); ^{13}C NMR (100 MHz, DMSO-d_6) δ 193.0, 173.0, 161.8, 136.5, 127.1, 121.8, 120.7, 118.2, 70.2, 42.5, 30.2.

Synthesis of 5a via sequent reduction and esterification of 3aa

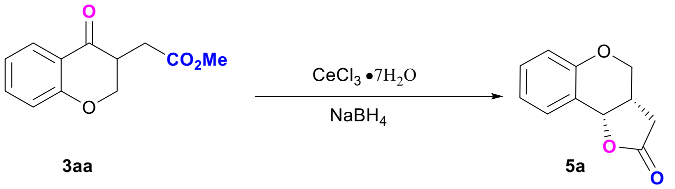

Add $CeCl_3.7H_2O$ (102 mg, 0.27 mmol) to a solution of methyl 2-(4-oxochroman-3-yl)acetate **3aa** (30 mg, 0.14 mmol) in CH_2Cl_2 (1.0 mL) and MeOH (0.7 mL). Cool the solution to -78 °C and add $NaBH_4$ (8 mg, 0.20 mmol) to the reaction mixture. Stir for 45 minutes and add 0.2 M HCl (5 mL) to the mixture. Then, extract mixture with $CHCl_3$ (3 x 5 mL) and the organic extracts were concentrated in vacuo. Dissolve residual crude alcohol in $CHCl_3$ (1 mL) and p-TsOH.H_2O (1 mg) is added. Stir the reaction mixture at room temperature for 90 minutes. Concentrate the solvent in vacuo. Purify by column chromatography (EtOAc/PE, 1:3) to obtain the desired product **5a**. ^{1}H NMR (400 MHz, Chloroform-*d*) δ 7.35 (d, *J* = 7.7 Hz, 1 H), 7.24 (t, *J* = 7.8 Hz, 1 H), 6.97 (t, *J* = 7.5 Hz, 1 H), 6.88 (d, *J* = 8.3 Hz, 1 H), 5.43 (dd, *J* = 6.4, 1.8 Hz, 1 H), 4.15 (dd, *J* = 11.5, 4.3 Hz,

1 H), 3.76 (t, *J* = 10.4 Hz, 1 H), 3.02 – 2.91 (m, 1 H), 2.81 (dd, *J* = 17.7, 8.3 Hz, 1 H), 2.39 (dd, *J* = 17.7, 3.6 Hz, 1 H); ^{13}C NMR (100 MHz, Chloroform-*d*) δ 175.3, 155.0, 131.2, 130.5, 121.7, 118.4, 117.2, 74.1, 64.7, 33.4, 31.0; HRMS (ESI): m/z $[M+H]^+$ calcd for $C_{11}H_{11}O_3$: 191.0703; found: 191.0705.

Density functional theory calculations

All DFT calculations were carried out with the Gaussian 16 software package[1] and were optimized at the B3LYP/6-31G(d) level of density functional theory (DFT).[2] Frequency calculations were carried out at the same level of theory to identify all of the stationary points as transition states (one imaginary frequency) or as minima (zero imaginary frequency). In order to consider the solvent effects, the solvation corrected single-point energy calculations (based on the gas-phase optimized geometries) were calculated by using the M062x/6-311++G(d, p) method in conjunction with the SMD solvation model[3] in solvent (Dimethyl sulfoxide). The single-point energy corrected relative free energies in kcal/mol are used for discussion throughout the text.

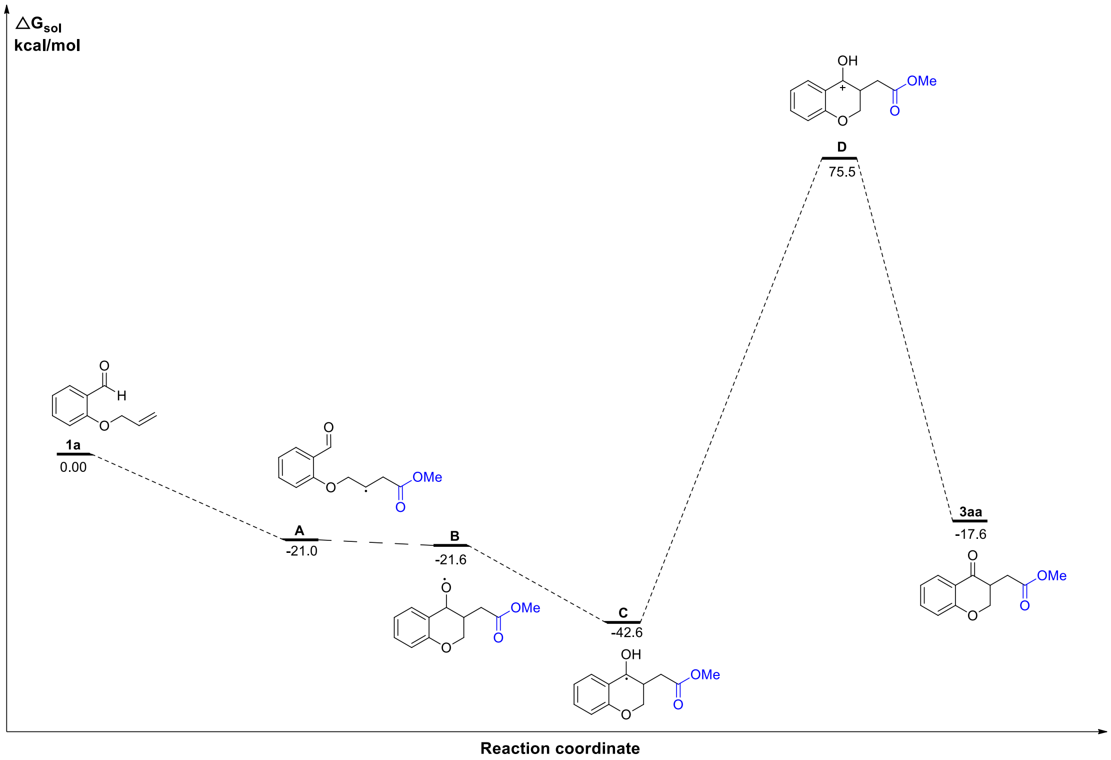

Figure S1. The free energy profiles for the cascade radical cyclization of **1a**. The free energies are reported in kcal/mol at the M062x/6-311++G(d, p) (DMSO) level of theory.

Cartesian Coordinates for Optimized Structures

1a

C	-2.82055000	1.34563800	-0.08024800
C	-1.72457300	2.21034000	-0.02611400
C	-0.41969500	1.71857800	0.03505900
C	-0.19835000	0.33482200	0.04407400
C	-1.30011900	-0.55138200	-0.01221900
C	-2.59784400	-0.02654000	-0.07346000

C	-1.11669600	-2.02029300	-0.00744600
O	-2.04131300	-2.81509400	-0.05050400
O	1.03581300	-0.23684100	0.09977800
C	2.19115600	0.60500800	0.20249200
C	3.39320600	-0.28040200	0.33070900
C	4.46978300	-0.18738900	-0.44784600
H	-3.83065400	1.74091000	-0.12789500
H	-1.88029300	3.28609300	-0.03247200
H	0.41030800	2.41404500	0.07207000
H	-3.41892800	-0.73593000	-0.11503600
H	-0.07063200	-2.37418600	0.03807400
H	2.27151700	1.25110300	-0.68187000
H	2.08734300	1.24821200	1.08961000
H	3.35166400	-1.01321500	1.13517100
H	5.33741000	-0.82395500	-0.29990200
H	4.52835900	0.53213000	-1.26193700

A

C	4.77655200	-1.10099000	-0.06582500
C	3.77480300	-2.07241100	-0.00144300
C	2.42583500	-1.71612600	0.04162700
C	2.06356800	-0.36244800	0.02141000
C	3.06911700	0.63092900	-0.04468900
C	4.41406900	0.24102000	-0.08741100
C	2.73701400	2.07404600	-0.06943300
O	3.57713600	2.95669900	-0.12636100
O	0.77743800	0.08107700	0.05736300
C	-0.28536300	-0.86812400	0.15829600
C	-1.56821000	-0.12273900	0.22466900
C	-2.85992700	-0.81950500	-0.02306600
C	-4.06118700	0.10980600	0.06618000
O	-4.03822200	1.26218300	0.43553400
O	-5.18374200	-0.53638500	-0.31413500
C	-6.39300500	0.24013300	-0.25095000
H	5.82227300	-1.39115300	-0.09908100
H	4.03999700	-3.12641900	0.01504200
H	1.67178500	-2.49265700	0.08853900
H	5.15831200	1.03012400	-0.13758600
H	1.66055500	2.32139900	-0.03294900
H	-0.26550500	-1.55316500	-0.70649400
H	-0.12735400	-1.49371600	1.05831500
H	-1.57296000	0.89062200	0.60914000
H	-2.88116500	-1.31553900	-1.00657300
H	-3.03801100	-1.64134100	0.69557300
H	-7.18629500	-0.42772900	-0.58741700

H	-6.58025800	0.57587700	0.77238000
H	-6.32184500	1.11355400	-0.90428600

B

C	-4.34915500	0.71100800	-0.24620200
C	-4.46317300	-0.68414800	-0.26760800
C	-3.33858200	-1.48435200	-0.10126300
C	-2.08381000	-0.89212100	0.09220400
C	-1.95438000	0.50683800	0.11404400
C	-3.09866300	1.29263000	-0.05950800
O	-1.02078400	-1.74309200	0.21433700
C	0.20733000	-1.18176500	0.67724500
C	0.55243600	0.14063800	-0.01187900
C	-0.58646400	1.14864900	0.29321800
O	-0.41707300	2.36012600	-0.30968400
C	1.91021800	0.69263100	0.42275700
C	3.08793000	-0.07136000	-0.15147400
O	3.01841500	-1.01002000	-0.91676400
O	4.25243300	0.43747700	0.30238300
C	5.44185200	-0.20418000	-0.19007600
H	-5.22714300	1.33571500	-0.38160800
H	-5.43396400	-1.15024300	-0.41351400
H	-3.40186600	-2.56787400	-0.11712900
H	-2.98292000	2.37262400	-0.06524500
H	0.96871900	-1.93467700	0.46808400
H	0.14569600	-1.03065600	1.76776600
H	0.56664300	-0.03394000	-1.09374900
H	-0.47454800	1.42336300	1.37365500
H	1.99062400	1.73920900	0.10577900
H	2.00894700	0.70423700	1.51731000
H	6.27301700	0.32683300	0.27436000
H	5.45335000	-1.26017200	0.09241800
H	5.49628800	-0.12713600	-1.27906100

C

C	3.64429400	-1.21893000	-0.73681200
C	4.08253000	0.10785500	-0.86431600
C	3.23760600	1.16126300	-0.49715500
C	1.97326000	0.89214900	0.00707100
C	1.50850000	-0.44906300	0.16859400
C	2.38287200	-1.49637000	-0.23334400
O	1.18740400	1.96173000	0.36774000
C	-0.21689700	1.71682500	0.38500100
C	-0.56127200	0.49611000	1.24870500
C	0.23186800	-0.67774700	0.72915400
O	-0.17202800	-1.95719100	0.98053200

C	-2.09179500	0.26236800	1.35304000
C	-2.71689100	-0.27685700	0.08517200
O	-2.60502300	-1.43436600	-0.28915200
O	-3.39852700	0.65093800	-0.59954500
C	-4.00011700	0.20652300	-1.83362000
H	4.29753000	-2.03600500	-1.03125600
H	5.07158400	0.32400700	-1.25785700
H	3.54467600	2.19757200	-0.60117200
H	2.03790900	-2.51850000	-0.12125000
H	-1.09703100	-2.05648000	0.66465800
H	-2.27623300	-0.48025100	2.13561300
H	-2.58338600	1.19473000	1.64468200
H	-4.49359400	1.08574600	-2.24675100
H	-3.23311300	-0.16603700	-2.51647400
H	-4.72382100	-0.58797200	-1.63704300
H	-0.58028000	1.57232700	-0.64336300
H	-0.66802300	2.62822500	0.78722000
H	-0.23458000	0.72898600	2.27746200

D

C	-4.35499800	0.76265000	-0.09857900
C	-4.48996800	-0.63484600	-0.07679500
C	-3.37394700	-1.45992400	-0.00669900
C	-2.09520300	-0.88963000	0.03950100
C	-1.94155000	0.51126300	-0.00393200
C	-3.08782500	1.32386700	-0.06476100
O	-1.03756200	-1.74083400	0.11407000
C	0.19069400	-1.17082100	0.58634400
C	0.56037500	0.11153800	-0.15957100
C	-0.59299400	1.11487600	-0.05967300
O	-0.40490400	2.32354400	-0.07252000
C	1.87680200	0.72080100	0.32316500
C	3.09508300	-0.05201700	-0.13957200
O	3.08481400	-1.00847600	-0.88711100
O	4.22182600	0.46979500	0.38753900
C	5.44553000	-0.17677700	-0.00310100
H	-5.23576200	1.39563200	-0.14841200
H	-5.47983000	-1.08269600	-0.10703400
H	-3.46405600	-2.54092900	0.02494400
H	-2.94024700	2.39890800	-0.09887800
H	0.94735300	-1.94154100	0.43511800
H	0.09055100	-0.96909700	1.66443500
H	0.66407100	-0.14779500	-1.22386000
H	1.94914000	1.75127600	-0.04233300
H	1.90543400	0.79965600	1.41770600

H	6.23880900	0.36514600	0.51224500
H	5.43797500	-1.22752400	0.29854900
H	5.58118100	-0.11915600	-1.08625400

3aa

C	-4.35499800	0.76265000	-0.09857900
C	-4.48996800	-0.63484600	-0.07679500
C	-3.37394700	-1.45992400	-0.00669900
C	-2.09520300	-0.88963000	0.03950100
C	-1.94155000	0.51126300	-0.00393200
C	-3.08782500	1.32386700	-0.06476100
O	-1.03756200	-1.74083400	0.11407000
C	0.19069400	-1.17082100	0.58634400
C	0.56037500	0.11153800	-0.15957100
C	-0.59299400	1.11487600	-0.05967300
O	-0.40490400	2.32354400	-0.07252000
C	1.87680200	0.72080100	0.32316500
C	3.09508300	-0.05201700	-0.13957200
O	3.08481400	-1.00847600	-0.88711100
O	4.22182600	0.46979500	0.38753900
C	5.44553000	-0.17677700	-0.00310100
H	-5.23576200	1.39563200	-0.14841200
H	-5.47983000	-1.08269600	-0.10703400
H	-3.46405600	-2.54092900	0.02494400
H	-2.94024700	2.39890800	-0.09887800
H	0.94735300	-1.94154100	0.43511800
H	0.09055100	-0.96909700	1.66443500
H	0.66407100	-0.14779500	-1.22386000
H	1.94914000	1.75127600	-0.04233300
H	1.90543400	0.79965600	1.41770600
H	6.23880900	0.36514600	0.51224500
H	5.43797500	-1.22752400	0.29854900
H	5.58118100	-0.11915600	-1.08625400

3. Characterization data of products

methyl 2-(4-oxochroman-3-yl)acetate **(3aa)**

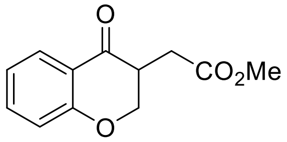

^{1}H NMR (400 MHz, Chloroform-*d*) δ 7.88 (d, *J* = 7.8 Hz, 1 H), 7.55 – 7.41 (m, 1 H), 7.08 – 6.94 (m, 2 H), 4.60 (dd, *J* = 11.2, 5.3 Hz, 1 H), 4.29 (t, *J* = 11.6 Hz, 1 H), 3.73 (s, 3 H), 3.34 (td, *J* = 12.8, 5.1 Hz, 1 H), 2.94 (dd, *J* = 17.0, 4.9 Hz, 1 H), 2.43 (dd, *J* = 17.0, 8.1 Hz, 1 H); ^{13}C NMR (100 MHz, Chloroform-*d*) δ 192.6, 171.8, 161.7, 136.0, 127.4, 121.5, 120.4, 117.8, 70.2, 52.0, 42.5, 30.1; HRMS (ESI): m/z $[M+H]^+$ calcd for $C_{12}H_{13}O_4$: 221.0808; found: 221.0811.

methyl 2-(8-methyl-4-oxochroman-3-yl)acetate **(3ba)**

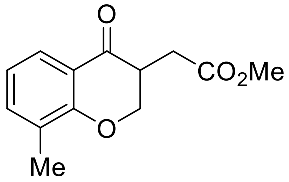

^{1}H NMR (400 MHz, Chloroform-*d*) δ 7.73 (d, *J* = 7.7 Hz, 1 H), 7.33 (d, *J* = 6.9 Hz, 1 H), 6.91 (t, *J* = 7.4 Hz, 1 H), 4.63 (dd, *J* = 11.0, 5.1 Hz, 1 H), 4.27 (t, *J* = 11.6 Hz, 1 H), 3.73 (s, 3 H), 3.31 (dt, *J* = 12.2, 6.2 Hz, 1 H), 2.94 (dd, *J* = 17.0, 4.7 Hz, 1 H), 2.42 (dd, *J* = 16.9, 8.0 Hz, 1 H), 2.23 (s, 3 H); ^{13}C NMR (100 MHz, Chloroform-*d*) δ 192.9, 171.9, 159.9, 136.8, 127.1, 124.9, 120.9, 120.0, 70.0, 52.0, 42.3, 30.1, 15.5; HRMS (ESI): m/z $[M+H]^+$ calcd for $C_{13}H_{15}O_4$: 235.0965; found: 235.0967.

methyl 2-(6-methyl-4-oxochroman-3-yl)acetate **(3ca)**

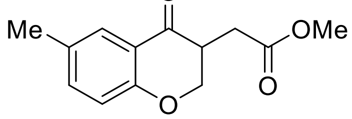

^{1}H NMR (400 MHz, Chloroform-*d*) δ 7.66 (s, 1 H), 7.29 (d, *J* = 8.4 Hz, 1 H), 6.87 (d, *J* = 8.4 Hz, 1 H), 4.56 (dd, *J* = 11.1, 5.2 Hz, 1 H), 4.25 (t, *J* = 11.5 Hz, 1 H), 3.72 (s, 3 H), 3.31 (dt, *J* = 12.4, 6.4 Hz, 1 H), 2.92 (dd, *J* = 17.0, 4.8 Hz, 1 H), 2.42 (dd, *J* = 17.0, 8.1 Hz, 1 H), 2.30 (s, 3 H); ^{13}C NMR (100 MHz, Chloroform-*d*) δ 192.8, 171.9, 159.8, 137.1, 131.0, 126.9, 120.0, 117.6, 70.2, 52.0, 42.5, 30.1, 20.4; HRMS (ESI): m/z $[M+H]^+$ calcd for $C_{13}H_{15}O_4$: 235.0965; found: 235.0968.

***methyl 2-(7-methoxy-4-oxochroman-3-yl)acetate* (3da)**

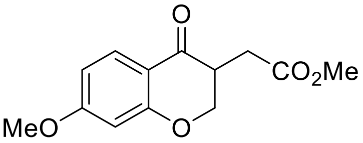

^{1}H NMR (400 MHz, Chloroform-*d*) δ 7.81 (d, *J* = 8.8 Hz, 1 H), 6.58 (dd, *J* = 8.8, 2.3 Hz, 1 H), 6.40 (d, *J* = 2.2 Hz, 1 H), 4.57 (dd, *J* = 11.1, 5.2 Hz, 1 H), 4.27 (t, *J* = 11.4 Hz, 1 H), 3.83 (s, 3 H), 3.72 (s, 3 H), 3.37 – 3.20 (m, 1 H), 2.94 (dd, *J* = 17.0, 4.8 Hz, 1 H), 2.40 (dd, *J* = 17.0, 8.3 Hz, 1 H); ^{13}C NMR (100 MHz, Chloroform-*d*) δ 191.2, 172.0, 166.0, 163.7, 129.1, 114.2, 110.1, 100.6, 70.5, 55.6, 52.0, 42.0, 30.1; HRMS (ESI): m/z $[M+H]^+$ calcd for $C_{13}H_{15}O_5$: 251.0914; found: 251.0917.

***methyl 2-(8-(tert-butyl)-4-oxochroman-3-yl)acetate* (3ea)**

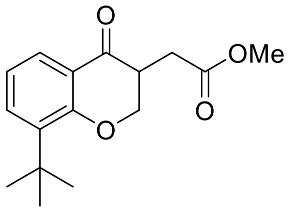

^{1}H NMR (400 MHz, Chloroform-*d*) δ 7.80 (d, *J* = 7.8 Hz, 1 H), 7.48 (d, *J* = 7.6 Hz, 1 H), 6.96 (t, *J* = 7.7 Hz, 1 H), 4.66 (dd, *J* = 11.0, 5.2 Hz, 1 H), 4.27 (t, *J* = 11.6 Hz, 1 H), 3.74 (s, 3 H), 3.33 (td, *J* = 12.9, 5.1 Hz, 1 H), 2.96 (dd, *J* = 17.0, 4.8 Hz, 1 H), 2.43 (dd, *J* = 17.0, 8.2 Hz, 1 H), 1.38 (s, 9 H); ^{13}C NMR (100 MHz, Chloroform-*d*) δ 193.2, 172.0, 160.9, 138.9, 133.0, 125.5, 121.2, 121.0, 69.7, 52.0, 42.4, 34.9, 30.2, 29.6; HRMS (ESI): m/z $[M+H]^+$ calcd for $C_{16}H_{21}O_4$: 277.1434; found: 277.1432.

***methyl 2-(7-fluoro-4-oxochroman-3-yl)acetate* (3fa)**

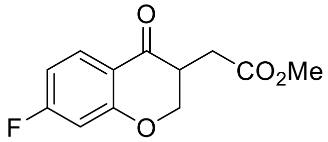

^{1}H NMR (400 MHz, Chloroform-*d*) δ 7.90 (dd, *J* = 8.8, 6.6 Hz, 1 H), 6.74 (td, *J* = 8.5, 2.3 Hz, 1 H), 6.66 (dd, *J* = 9.8, 2.3 Hz, 1 H), 4.61 (dd, *J* = 11.2, 5.3 Hz, 1 H), 4.31 (t, *J* = 11.7 Hz, 1 H), 3.73 (s, 3 H), 3.43 – 3.24 (m, 1 H), 2.93 (dd, *J* = 17.1, 4.9 Hz, 1 H), 2.43 (dd, *J* = 17.1, 8.0 Hz, 1 H); ^{13}C NMR (100 MHz, Chloroform-*d*) δ 191.2, 171.7, 167.4 (d, $J_{C\text{-}F}$ = 254.0 Hz), 163.3 (d, $J_{C\text{-}F}$ = 14.0 Hz), 129.9 (d, $J_{C\text{-}F}$ = 12.0 Hz), 117.4, 110.0 (d, $J_{C\text{-}F}$ = 22.0 Hz), 104.6 (d, $J_{C\text{-}F}$ = 24.0 Hz), 104.5 , 70.6 , 52.1 , 42.1 , 29.9; ^{19}F NMR (376 MHz, Chloroform-*d*) δ -100.27; HRMS (ESI): m/z $[M+H]^+$ calcd for $C_{12}H_{12}FO_4$: 239.0714; found: 239.0710.

***methyl 2-(6-fluoro-4-oxochroman-3-yl)acetate* (3ga)**

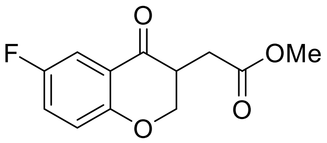

^{1}H NMR (400 MHz, Chloroform-*d*) δ 7.52 (dd, *J* = 8.2, 3.1 Hz, 1 H), 7.20 (td, *J* = 8.4, 3.2 Hz, 1 H), 6.95 (dd, *J* = 9.1, 4.2 Hz, 1 H), 4.58 (dd, *J* = 11.2, 5.3 Hz, 1 H), 4.28 (t, *J* = 11.7 Hz, 1 H), 3.72 (s, 3 H), 3.31 (td, *J* = 12.7, 5.1 Hz, 1 H), 2.91 (dd, *J* = 17.1, 4.8 Hz, 1 H), 2.44 (dd, *J* = 17.1, 8.0 Hz, 1 H); ^{13}C NMR (100 MHz, Chloroform-*d*) δ 191.9, 171.6, 158.0 (d, $J_{C\text{-}F}$ = 1Hz), 157.2 (d, $J_{C\text{-}F}$ = 241.0 Hz), 123.5 (d, $J_{C\text{-}F}$ = 25.0 Hz), 120.8 (d, $J_{C\text{-}F}$ = 6.0 Hz), 119.5 (d, $J_{C\text{-}F}$ = 7.0 Hz), 112.2 (d, $J_{C\text{-}F}$ =23.0 Hz), 70.3, 52.1, 42.3, 29.9; ^{19}F NMR (376 MHz, Chloroform-*d*) δ -121.24; HRMS (ESI): m/z $[M+H]^+$ calcd for $C_{12}H_{12}FO_4$: 239.0714; found: 239.0715.

methyl 2-(8-chloro-4-oxochroman-3-yl)acetate **(3ha)**

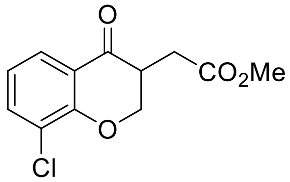

^{1}H NMR (400 MHz, Chloroform-*d*) δ 7.80 (dd, *J* = 7.9, 1.3 Hz, 1 H), 7.57 (dd, *J* = 7.8, 1.3 Hz, 1 H), 6.98 (t, *J* = 7.8 Hz, 1 H), 4.74 (dd, *J* = 11.2, 5.3 Hz, 1 H), 4.40 (t, *J* = 11.8 Hz, 1 H), 3.73 (s, 3 H), 3.44 – 3.31 (m, 1 H), 2.92 (dd, *J* = 17.1, 4.9 Hz, 1 H), 2.48 (dd, *J* = 17.1, 7.8 Hz, 1 H); ^{13}C NMR (100 MHz, Chloroform-*d*) δ 191.8, 171.5, 157.1, 136.1, 126.0, 122.5, 121.7, 121.6, 70.7, 52.1, 42.1, 29.9; HRMS (ESI): m/z $[M+H]^+$ calcd for $C_{12}H_{12}ClO_4$: 255.0419; found: 255.0424.

methyl 2-(7-chloro-4-oxochroman-3-yl)acetate **(3ia)**

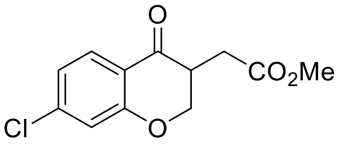

^{1}H NMR (400 MHz, Chloroform-*d*) δ 7.81 (d, *J* = 8.9 Hz, 1 H), 7.00 (dd, *J* = 4.4, 2.6 Hz, 2 H), 4.61 (dd, *J* = 11.2, 5.3 Hz, 1 H), 4.31 (t, *J* = 11.7 Hz, 1 H), 3.73 (s, 3 H), 3.32 (td, *J* = 12.8, 5.1 Hz, 1 H), 2.92 (dd, *J* = 17.1, 4.8 Hz, 1 H), 2.44 (dd, *J* = 17.1, 8.0 Hz, 1 H); ^{13}C NMR (100 MHz, Chloroform-*d*) δ 191.5, 171.6, 162.0, 141.9, 128.6, 122.4, 119.0, 118.0, 70.5, 52.1, 42.3, 29.9; HRMS (ESI): m/z $[M+H]^+$ calcd for $C_{12}H_{12}ClO_4$: 255.0419; found: 255.0422.

methyl 2-(5-chloro-4-oxochroman-3-yl)acetate **(3ja)**

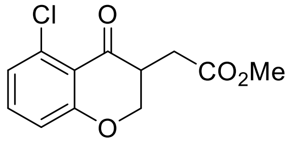

^{1}H NMR (400 MHz, Chloroform-*d*) δ 7.33 (t, *J* = 8.1 Hz, 1 H), 7.04 (d, *J* = 7.8 Hz, 1 H), 6.90 (d, *J* = 8.4 Hz, 1 H), 4.58 (dd, *J* = 11.2, 5.3 Hz, 1 H), 4.29 (t, *J* = 11.7 Hz, 1 H), 3.73 (s, 3 H), 3.36 (td, *J* = 12.8, 5.3 Hz, 1 H), 2.92 (dd, *J* = 17.0, 5.2 Hz, 1 H), 2.41 (dd, *J* = 17.0, 7.7 Hz, 1 H); ^{13}C NMR (100 MHz, Chloroform-*d*) δ 190.7, 171.7, 163.0, 134.8, 134.5, 124.7, 117.7, 116.9, 69.7, 52.1, 43.0, 30.0; HRMS (ESI): m/z $[M+H]^+$ calcd for $C_{12}H_{12}ClO_4$: 255.0419; found: 255.0426.

methyl 2-(7-bromo-4-oxochroman-3-yl)acetate **(3ka)**

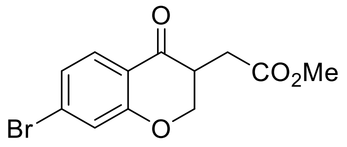

^{1}H NMR (400 MHz, Chloroform-*d*) δ 7.67 (d, *J* = 8.3 Hz, 1 H), 7.10 (dd, *J* = 10.8, 2.4 Hz, 2 H), 4.54 (dd, *J* = 11.2, 5.3 Hz, 1 H), 4.24 (t, *J* = 11.7 Hz, 1 H), 3.66 (s, 3 H), 3.25 (td, *J* = 12.8, 5.1 Hz, 1 H), 2.86 (dd, *J* = 17.1, 4.8 Hz, 1 H), 2.37 (dd, *J* = 17.1, 8.0 Hz, 1 H); ^{13}C NMR (100 MHz, Chloroform-*d*) δ 191.7, 171.6, 161.9, 130.5, 128.6, 125.2, 121.0, 119.3, 70.5, 52.1, 42.3, 29.9; HRMS (ESI): m/z [M+H]$^+$ calcd for $C_{12}H_{12}BrO_4$: 298.9913; found: 298.9909.

methyl 2-(6-bromo-4-oxochroman-3-yl)acetate **(3la)**

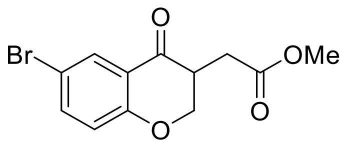

^{1}H NMR (400 MHz, Chloroform-*d*) δ 7.97 (d, *J* = 2.4 Hz, 1 H), 7.54 (dd, *J* = 8.8, 2.4 Hz, 1 H), 6.88 (d, *J* = 8.8 Hz, 1 H), 4.60 (dd, *J* = 11.2, 5.3 Hz, 1 H), 4.29 (t, *J* = 11.7 Hz, 1 H), 3.72 (s, 3 H), 3.31 (td, *J* = 12.7, 5.1 Hz, 1 H), 2.92 (dd, *J* = 17.1, 4.8 Hz, 1 H), 2.44 (dd, *J* = 17.1, 8.0 Hz, 1 H); ^{13}C NMR (100 MHz, Chloroform-*d*) δ 191.3, 171.6, 160.6, 138.6, 129.7, 121.7, 119.9, 114.2, 70.2, 52.1, 42.2, 29.9; HRMS (ESI): m/z [M+H]$^+$ calcd for $C_{12}H_{12}BrO_4$: 298.9913; found: 298.9915.

methyl 3-(2-methoxy-2-oxoethyl)-4-oxochromane-6-carboxylate **(3ma)**

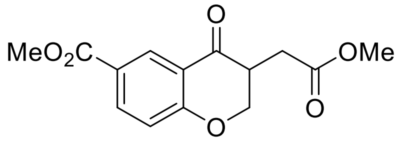

^{1}H NMR (400 MHz, Chloroform-*d*) δ 8.56 (d, *J* = 2.1 Hz, 1 H), 8.13 (dd, *J* = 8.7, 2.1 Hz, 1 H), 7.02 (d, *J* = 8.7 Hz, 1 H), 4.67 (dd, *J* = 11.3, 5.4 Hz, 1 H), 4.35 (t, *J* = 11.8 Hz, 1 H), 3.89 (s, 3 H), 3.73 (s, 3 H), 3.36 (dt, *J* = 12.4, 6.3 Hz, 1 H), 2.96 (dd, *J* = 17.1, 4.7 Hz, 1 H), 2.45 (dd, *J* = 17.1, 8.0 Hz, 1 H); ^{13}C NMR (100 MHz, Chloroform-*d*) δ 191.5, 171.6, 165.9, 164.7, 136.7, 129.8, 123.8, 120.0, 118.2, 70.4, 52.1, 42.3, 29.9; HRMS (ESI): m/z [M+H]$^+$ calcd for $C_{14}H_{15}O_6$: 279.0863; found: 279.0869.

methyl 2-(6-nitro-4-oxochroman-3-yl)acetate **(3na)**

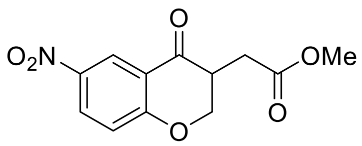

^{1}H NMR (400 MHz, Chloroform-*d*) δ 8.75 (d, *J* = 2.6 Hz, 1 H), 8.32 (dd, *J* = 9.1, 2.6 Hz, 1 H), 7.11 (d, *J* = 9.1 Hz, 1 H), 4.75 (dd, *J* = 11.3, 5.5 Hz, 1 H), 4.43 (t, *J* = 12.0 Hz, 1 H), 3.73 (s, 3 H), 3.39 (td, *J* = 12.6, 5.2 Hz, 1 H), 3.05 – 2.90 (m, 1 H), 2.51 (dd, *J* = 17.3, 7.7 Hz, 1 H); ^{13}C NMR (100 MHz, Chloroform-*d*) δ 190.5, 171.3, 165.6, 142.2, 130.3, 123.9, 120.0, 119.2, 70.6, 52.2, 42.1, 29.6; HRMS (ESI): m/z [M+H]$^+$ calcd for $C_{12}H_{12}NO_6$: 266.0659; found: 266.0664.

methyl 2-(7-(benzyloxy)-4-oxochroman-3-yl)acetate **(3oa)**

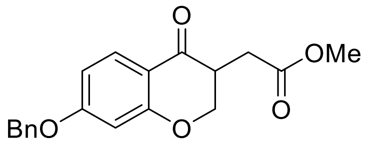

^{1}H NMR (400 MHz, Chloroform-*d*) δ 7.83 (d, *J* = 8.8 Hz, 1 H), 7.38 (dd, *J* = 14.3, 5.8 Hz, 5 H), 6.66 (d, *J* = 8.8 Hz, 1 H), 6.48 (s, 1 H), 5.08 (s, 2 H), 4.57 (dd, *J* = 11.1, 5.2 Hz, 1 H), 4.26 (t, *J* = 11.5 Hz, 1 H), 3.72 (s, 3 H), 3.27 (dq, *J* = 12.4, 5.0 Hz, 1 H), 2.93 (dd, *J* = 17.0, 4.7 Hz, 1 H), 2.40 (dd, *J* = 17.0, 8.3 Hz, 1 H); ^{13}C NMR (100 MHz, Chloroform-*d*) δ 191.1, 172.0, 165.1, 163.6, 135.8, 129.1, 128.7, 128.3, 127.5, 114.4, 110.6, 101.6, 70.5, 70.3, 52.0, 42.0, 30.1; HRMS (ESI): m/z [M+H]$^+$ calcd for $C_{19}H_{19}O_5$: 327.1227; found: 327.1236.

methyl 2-(6,8-dichloro-4-oxochroman-3-yl)acetate **(3pa)**

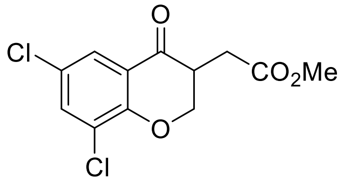

^{1}H NMR (400 MHz, Chloroform-*d*) δ 7.76 (d, *J* = 2.4 Hz, 1 H), 7.55 (d, *J* = 2.4 Hz, 1 H), 4.74 (dd, *J* = 11.3, 5.4 Hz, 1 H), 4.39 (t, *J* = 11.8 Hz, 1 H), 3.73 (s, 3 H), 3.35 (td, *J* = 12.6, 5.1 Hz, 1 H), 2.91 (dd, *J* = 17.2, 4.7 Hz, 1 H), 2.50 (dd, *J* = 17.3, 7.7 Hz, 1 H); ^{13}C NMR (100 MHz, Chloroform-*d*) δ 190.7, 171.3, 155.9, 135.6, 126.8, 125.4, 123.7, 122.0, 70.8, 52.2, 42.0, 29.8; HRMS (ESI): m/z [M+H]$^+$ calcd for $C_{12}H_{11}Cl_2O_4$: 289.0029; found: 289.0031.

methyl 2-(4-oxo-3,4-dihydro-2H-benzo[h]chromen-3-yl)acetate **(3qa)**

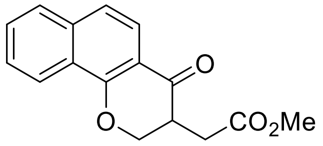

^{1}H NMR (400 MHz, Chloroform-*d*) δ 9.42 (d, *J* = 8.7 Hz, 1 H), 7.92 (d, *J* = 9.1 Hz, 1 H), 7.75 (d, *J* = 8.2 Hz, 1 H), 7.62 (t, *J* = 7.8 Hz, 1 H), 7.43 (t, *J* = 7.5 Hz, 1 H), 7.10 (d, *J* = 9.0 Hz, 1 H), 4.68 (dd, *J* = 11.1, 5.2 Hz, 1 H), 4.41 (t, *J* = 11.5 Hz, 1 H), 3.75 (s, 3 H), 3.43 (dq, *J* = 12.7, 5.2 Hz, 1 H), 2.99 (dd, *J* = 16.8, 5.1 Hz, 1 H), 2.48 (dd, *J* = 16.8, 8.0 Hz, 1 H); ^{13}C NMR (100 MHz, Chloroform-*d*) δ 193.5, 172.1, 163.7, 137.6, 131.6, 129.7, 129.2, 128.4, 125.8, 124.9, 118.6, 112.0, 70.1, 52.0, 43.0, 30.5; HRMS (ESI): m/z [M+H]$^+$ calcd for $C_{16}H_{15}O_4$: 271.0965; found: 271.0959.

methyl 2-(3-methyl-4-oxochroman-3-yl)acetate **(3ra)**

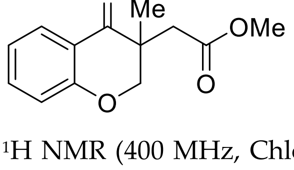

^{1}H NMR (400 MHz, Chloroform-*d*) δ 7.90 (d, *J* = 7.8 Hz, 1 H), 7.47 (t, *J* = 7.7 Hz, 1 H), 7.11 – 6.85 (m, 2 H), 4.63 (d, *J* = 11.4 Hz, 1 H), 4.23 (d, *J* = 11.4 Hz, 1 H), 3.66 (s, 3 H), 2.81 (d, *J* = 16.0 Hz, 1 H), 2.52 (d, *J* = 16.0 Hz, 1 H), 1.29 (s, 3 H); ^{13}C NMR (100 MHz, Chloroform-*d*) δ 195.3, 171.1, 161.0, 135.8, 127.9, 121.7, 119.4, 117.7, 74.2, 51.7, 43.7, 37.6, 19.1; HRMS (ESI): m/z [M+H]$^+$ calcd for $C_{13}H_{15}O_4$: 235.0965; found: 235.0969.

methyl 2-(1-oxo-2,3-dihydro-1H-inden-2-yl)acetate **(3sa)**

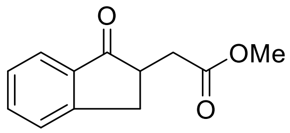

^{1}H NMR (400 MHz, Chloroform-*d*) δ 7.77 (d, *J* = 7.6 Hz, 1 H), 7.60 (t, *J* = 7.4 Hz, 1 H), 7.46 (d, *J* = 7.6 Hz, 1 H), 7.38 (t, *J* = 7.5 Hz, 1 H), 3.69 (s, 3 H), 3.47 (dd, *J* = 17.1, 8.0 Hz, 1 H), 3.06 – 2.96 (m, 2 H), 2.89 (dd, *J* = 17.1, 4.2 Hz, 1 H), 2.67 – 2.57 (m, 1 H); ^{13}C NMR (100 MHz, Chloroform-*d*) δ 206.7, 172.5, 153.2, 136.3, 134.9, 127.5, 126.5, 124.0, 51.9, 43.6, 35.0, 33.0; HRMS (ESI): m/z [M+H]$^+$ calcd for $C_{12}H_{13}O_3$: 205.0859; found: 205.0862.

***ethyl 2-(4-oxochroman-3-yl)acetate* (3ab)**

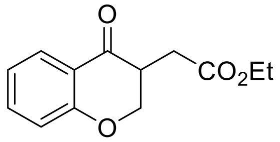

^{1}H NMR (400 MHz, Chloroform-*d*) δ 7.88 (d, *J* = 6.9 Hz, 1 H), 7.47 (t, *J* = 7.8 Hz, 1 H), 7.06 – 6.88 (m, 2 H), 4.59 (dd, *J* = 11.2, 5.3 Hz, 1 H), 4.29 (t, *J* = 11.6 Hz, 1 H), 4.18 (q, *J* = 8.5, 6.9 Hz, 2 H), 3.33 (td, *J* = 12.9, 5.1 Hz, 1 H), 2.92 (dd, *J* = 17.0, 4.8 Hz, 1 H), 2.41 (dd, *J* = 17.0, 8.1 Hz, 1 H), 1.28 (t, *J* = 7.1 Hz, 3 H); ^{13}C NMR (100 MHz, Chloroform-*d*) δ 192.6, 171.3, 161.7, 136.0, 127.3, 121.5, 120.4, 117.8, 70.2, 61.0, 42.5, 30.3, 14.1; HRMS (ESI): m/z [M+H]$^+$ calcd for $C_{13}H_{15}O_4$: 235.0965; found: 235.0967.

***butyl 2-(4-oxochroman-3-yl)acetate* (3ac)**

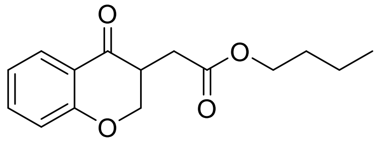

^{1}H NMR (400 MHz, Chloroform-*d*) δ 7.89 (d, *J* = 7.8 Hz, 1 H), 7.47 (t, *J* = 7.7 Hz, 1 H), 7.11 – 6.86 (m, 2 H), 4.60 (dd, *J* = 11.2, 5.2 Hz, 1 H), 4.29 (t, *J* = 11.6 Hz, 1 H), 4.13 (tq, *J* = 7.0, 4.1 Hz, 2 H), 3.32 (td, *J* = 12.9, 5.0 Hz, 1 H), 2.94 (dd, *J* = 16.9, 4.7 Hz, 1 H), 2.42 (dd, *J* = 16.9, 8.2 Hz, 1 H), 1.63 (t, *J* = 7.3 Hz, 2 H), 1.39 (dq, *J* = 14.7, 7.4 Hz, 2 H), 0.94 (t, *J* = 7.4 Hz, 3 H); ^{13}C NMR (100 MHz, Chloroform-*d*) δ 192.6, 171.4, 161.7, 136.0, 127.4, 121.5, 120.5, 117.8, 70.2, 64.9, 42.5, 30.6, 30.4, 19.1, 13.7; HRMS (ESI): m/z [M+H]$^+$ calcd for $C_{15}H_{19}O_4$: 263.1278; found: 263.1273.

***hexyl 2-(4-oxochroman-3-yl)acetate* (3ad)**

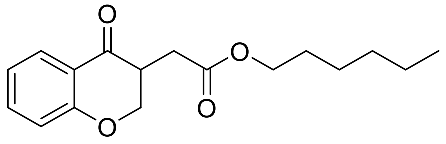

^{1}H NMR (400 MHz, Chloroform-*d*) δ 7.88 (d, *J* = 7.8 Hz, 1 H), 7.48 (t, *J* = 8.2 Hz, 1 H), 7.13 – 6.83 (m, 2 H), 4.60 (dd, *J* = 11.2, 5.3 Hz, 1 H), 4.29 (t, *J* = 11.6 Hz, 1 H), 4.11 (t, *J* = 8.1 Hz, 2 H), 3.33 (td, *J* = 12.9, 5.0 Hz, 1 H), 2.94 (dd, *J* = 17.0, 4.7 Hz, 1 H), 2.42 (dd, *J* = 17.0, 8.2 Hz, 1 H), 1.62 (q, *J* = 7.0 Hz, 2 H), 1.32 (d, *J* = 16.3 Hz, 6 H), 0.88 (t, *J* = 6.6 Hz, 3 H); ^{13}C NMR (100 MHz, Chloroform-*d*) δ 192.6, 171.4, 161.7, 136.0, 127.4, 121.5, 120.5, 117.8, 70.2, 65.2, 42.5, 31.4, 30.3, 28.5, 25.5, 22.5, 14.0; HRMS (ESI): m/z [M+H]$^+$ calcd for $C_{17}H_{23}O_4$: 291.1591; found: 291.1592.

***isobutyl 2-(4-oxochroman-3-yl)acetate* (3ae)**

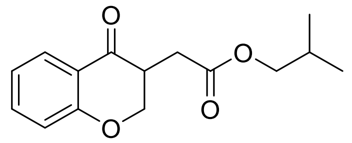

^{1}H NMR (400 MHz, Chloroform-*d*) δ 7.88 (d, *J* = 7.8 Hz, 1 H), 7.47 (t, *J* = 7.7 Hz, 1 H), 7.09 – 6.90 (m, 2 H), 4.60 (dd, *J* = 11.2, 5.2 Hz, 1 H), 4.30 (t, *J* = 11.5 Hz, 1 H), 3.92 (dq, *J* = 9.1, 5.3, 3.7 Hz, 2 H), 3.33 (td, *J* = 12.7, 5.0 Hz, 1 H), 2.95 (dd, *J* = 16.9, 4.7 Hz, 1 H), 2.43 (dd, *J* = 16.9, 8.2 Hz, 1 H), 1.99 – 1.88 (m, 1 H), 0.94 (d, *J* = 6.7 Hz, 6 H); ^{13}C NMR (100 MHz, Chloroform-*d*) δ 192.5, 171.4, 161.7, 136.0, 127.4, 121.5, 120.5, 117.8, 71.1, 70.2, 42.5, 30.3, 27.7, 19.0; HRMS (ESI): m/z [M+H]$^+$ calcd for $C_{15}H_{19}O_4$: 263.1278; found: 263.1274.

***3-methylpentyl 2-(4-oxochroman-3-yl)acetate* (3af)**

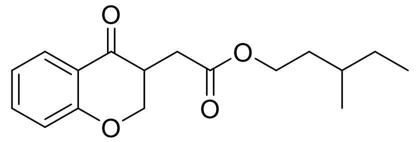

^{1}H NMR (400 MHz, Chloroform-*d*) δ 7.89 (dd, *J* = 7.9, 1.7 Hz, 1 H), 7.55 – 7.43 (m, 1 H), 7.09 – 6.92 (m, 2 H), 4.60 (dd, *J* = 11.2, 5.2 Hz, 1 H), 4.30 (t, *J* = 11.6 Hz, 1 H), 4.15 (td, *J* = 11.8, 10.7, 5.2 Hz, 2 H), 3.33 (td, *J* = 12.8, 5.0 Hz, 1 H), 2.94 (dd, *J* = 16.9, 4.7 Hz, 1 H), 2.42 (dd, *J* = 17.0, 8.2 Hz, 1 H), 1.68 (q, *J* = 7.8 Hz, 1 H), 1.49 – 1.33 (m, 3 H), 1.19 (dt, *J* = 13.8, 7.1 Hz, 1 H), 0.92 – 0.85 (m, 6 H); ^{13}C NMR (1010 MHz, Chloroform-*d*) δ 192.6, 171.4, 161.7, 136.0, 127.4, 121.5, 120.5, 117.8, 70.2, 63.7, 42.5, 35.0, 31.4, 30.4, 29.4, 19.0, 11.2; HRMS (ESI): m/z [M+H]$^+$ calcd for $C_{17}H_{23}O_4$: 291.1591; found: 291.1596.

***3-phenylpropyl 2-(4-oxochroman-3-yl)acetate* (3ag)**

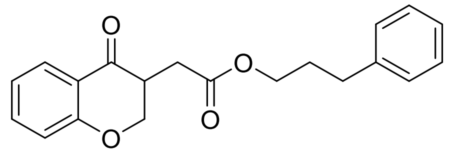

^{1}H NMR (400 MHz, Chloroform-*d*) δ 7.90 (d, *J* = 7.8 Hz, 1 H), 7.48 (t, *J* = 7.7 Hz, 1 H), 7.33 – 7.26 (m, 2 H), 7.20 (t, *J* = 6.6 Hz, 3 H), 7.03 (t, *J* = 7.5 Hz, 1 H), 6.98 (d, *J* = 8.4 Hz, 1 H), 4.60 (dd, *J* = 11.2, 5.3 Hz, 1 H), 4.30 (t, *J* = 11.6 Hz, 1 H), 4.15 (tt, *J* = 7.2, 3.7 Hz, 2 H), 3.39 – 3.26 (m, 1 H), 2.93 (dd, *J* = 16.9, 4.9 Hz, 1 H), 2.70 (t, *J* = 7.6 Hz, 2 H), 2.43 (dd, *J* = 16.9, 8.0 Hz, 1 H), 1.99 (dt, *J* = 13.6, 6.8 Hz, 2 H); ^{13}C NMR (100 MHz, Chloroform-*d*) δ 192.5, 171.3, 161.7, 141.0, 136.0, 128.4, 128.4, 127.3, 126.0, 121.5, 120.5, 117.8, 70.2, 64.3, 42.5, 32.1, 30.3, 30.1; HRMS (ESI): m/z [M+H]$^+$ calcd for $C_{20}H_{21}O_4$: 325.1434; found: 325.1437.

***2-fluoroethyl 2-(4-oxochroman-3-yl)acetate* (3ah)**

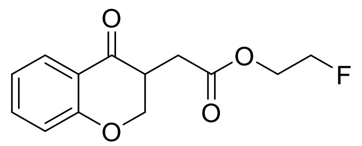

^{1}H NMR (400 MHz, Chloroform-*d*) δ 7.88 (d, *J* = 7.8 Hz, 1 H), 7.48 (t, *J* = 7.7 Hz, 1 H), 7.11 – 6.84 (m, 2 H), 4.74 – 4.50 (m, 3 H), 4.47 – 4.21 (m, 3 H), 3.36 (dq, *J* = 12.3, 5.5 Hz, 1 H), 2.98 (dd, *J* = 17.0, 5.0 Hz, 1 H), 2.49 (dd, *J* = 17.0, 7.8 Hz, 1 H); ^{13}C NMR (100 MHz, Chloroform-*d*) δ 192.4, 171.2, 161.7, 136.1, 127.4, 121.5, 120.4, 117.8, 81.2 (d, $J_{C\text{-}F}$ = 170.0 Hz), 70.1, 63.7 (d, $J_{C\text{-}F}$ = 20.0 Hz), 42.4, 30.2; ^{19}F NMR (376 MHz, Chloroform-*d*) δ -216.5; HRMS (ESI): m/z [M+H]$^+$ calcd for

$C_{13}H_{14}FO_4$: 253.0871; found: 253.0873.

2-ethoxyethyl 2-(4-oxochroman-3-yl)acetate **(3ai)**

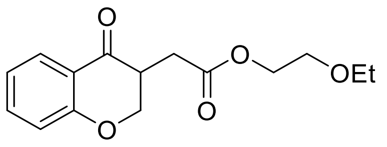

^{1}H NMR (400 MHz, Chloroform-*d*) δ 7.89 (d, *J* = 7.8 Hz, 1 H), 7.48 (t, *J* = 7.7 Hz, 1 H), 7.12 – 6.91 (m, 2 H), 4.60 (dd, *J* = 11.1, 5.2 Hz, 1 H), 4.43 – 4.14 (m, 3 H), 3.72 – 3.58 (m, 2 H), 3.53 (q, *J* = 6.9 Hz, 2 H), 3.34 (dq, *J* = 12.6, 5.3 Hz, 1 H), 2.99 (dd, *J* = 17.0, 4.6 Hz, 1 H), 2.47 (dd, *J* = 17.0, 8.2 Hz, 1 H), 1.21 (t, *J* = 6.9 Hz, 3 H); ^{13}C NMR (100 MHz, Chloroform-*d*) δ 192.5, 171.4, 161.7, 136.0, 127.4, 121.5, 120.5, 117.8, 70.2, 68.2, 66.6, 64.1, 42.5, 30.3, 15.1; HRMS (ESI): m/z $[M+H]^+$ calcd for $C_{15}H_{19}O_5$: 279.1227; found: 279.1223.

4,4,4-trifluorobutyl 2-(4-oxochroman-3-yl)acetate **(3aj)**

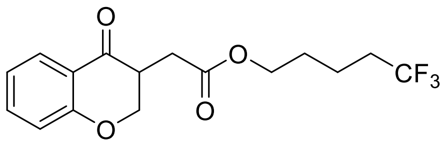

^{1}H NMR (400 MHz, Chloroform-*d*) δ 7.88 (dd, *J* = 7.9, 1.6 Hz, 1 H), 7.58 – 7.41 (m, 1 H), 7.11 – 6.90 (m, 2 H), 4.59 (dd, *J* = 11.1, 5.3 Hz, 1 H), 4.40 – 4.25 (m, 2 H), 4.24 – 4.12 (m, 2 H), 3.34 (dq, *J* = 12.3, 5.4 Hz, 1 H), 2.90 (dd, *J* = 17.0, 5.4 Hz, 1 H), 2.45 (dd, *J* = 16.9, 7.5 Hz, 1 H), 2.20 (ddd, *J* = 10.7, 7.7, 5.2 Hz, 2 H), 2.03 (dq, *J* = 13.1, 6.6 Hz, 1 H), 1.93 (dq, *J* = 13.1, 6.6 Hz, 2 H); ^{13}C NMR (100 MHz, Chloroform-*d*) δ 192.5, 171.2, 161.7, 136.1, 127.3, 126.8 (q, $J_{C\text{-}F}$ = 274.0 Hz), 121.6, 120.4, 117.8, 70.2, 63.1, 42.5, 30.6 (q, $J_{C\text{-}F}$ = 29.0 Hz), 30.2, 21.5; ^{19}F NMR (376 MHz, Chloroform-*d*) δ -66.4; HRMS (ESI): m/z $[M+H]^+$ calcd for $C_{16}H_{18}F_3O_4$: 331.1152; found: 331.1146.

cyclobutyl 2-(4-oxochroman-3-yl)acetate **(3ak)**

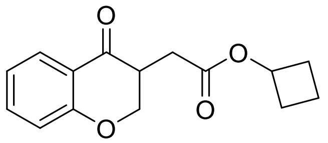

^{1}H NMR (400 MHz, Chloroform-*d*) δ 7.88 (d, *J* = 7.8 Hz, 1 H), 7.47 (t, *J* = 7.7 Hz, 1 H), 7.15 – 6.90 (m, 2 H), 5.06 – 4.97 (m, 1 H), 4.58 (dd, *J* = 11.1, 5.2 Hz, 1 H), 4.28 (t, *J* = 11.6 Hz, 1 H), 3.31 (td, *J* = 12.6, 5.1 Hz, 1 H), 2.90 (dd, *J* = 16.9, 4.7 Hz, 1 H), 2.38 (dd, *J* = 16.8, 8.1 Hz, 3 H), 2.15 – 2.00 (m, 2 H), 1.80 (q, *J* = 10.2 Hz, 1 H), 1.63 (dt, *J* = 18.7, 10.8 Hz, 1 H); ^{13}C NMR (100 MHz, Chloroform-*d*) δ 192.5, 170.6, 161.7, 135.9, 127.3, 121.5, 120.4, 117.8, 70.2, 69.3, 42.5, 30.3, 30.2, 30.2, 13.5; HRMS (ESI): m/z $[M+H]^+$ calcd for $C_{15}H_{17}O_4$: 261.1121; found: 261.1125.

cyclohexyl 2-(4-oxochroman-3-yl)acetate **(3al)**

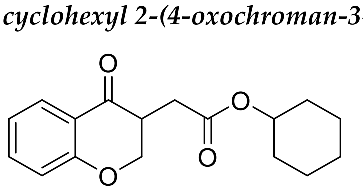

^{1}H NMR (400 MHz, Chloroform-*d*) δ 7.89 (d, *J* = 7.8 Hz, 1 H), 7.48 (t, *J* = 7.8 Hz, 1 H), 7.12 – 6.88 (m, 2 H), 4.81 (td, *J* = 8.8, 4.1 Hz, 1 H), 4.60 (dd, *J* = 11.1, 5.2 Hz, 1 H), 4.30 (t, *J* = 11.5 Hz, 1 H), 3.32 (td, *J* = 12.7, 12.3, 5.1 Hz, 1 H), 2.92 (dd, *J* = 16.8, 4.7 Hz, 1 H), 2.40 (dd, *J* = 16.8, 8.3 Hz,

1 H), 1.98 – 1.68 (m, 4 H), 1.54 – 1.27 (m, 6 H); ^{13}C NMR (100 MHz, Chloroform-*d*) δ 192.6, 170.7, 161.7, 136.0, 127.4, 121.5, 120.5, 117.8, 73.4, 70.3, 42.6, 31.6, 30.7, 25.3, 23.7; HRMS (ESI): m/z [M+H]$^+$ calcd for $C_{17}H_{21}O_4$: 289.1434; found: 289.1439.

3-neopentylchroman-4-one **(3am)**

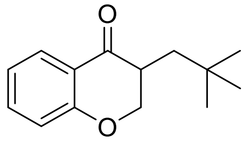

^{1}H NMR (400 MHz, Chloroform-*d*) δ 7.89 (d, *J* = 7.8 Hz, 1 H), 7.46 (t, *J* = 7.7 Hz, 1 H), 7.01 (t, *J* = 7.5 Hz, 1 H), 6.95 (d, *J* = 8.3 Hz, 1 H), 4.50 (dd, *J* = 11.3, 5.0 Hz, 1 H), 4.18 (t, *J* = 11.1 Hz, 1 H), 2.72 (dq, *J* = 10.3, 4.8 Hz, 1 H), 2.06 (dd, *J* = 14.3, 3.6 Hz, 1 H), 1.05 (dd, *J* = 14.3, 5.7 Hz, 1 H), 0.97 (s, 9 H); ^{13}C NMR (100 MHz, Chloroform-*d*) δ 194.7, 161.4, 135.5, 127.5, 121.2, 120.8, 117.6, 71.9, 42.8, 38.3, 30.7, 29.4; HRMS (ESI): m/z [M+H]$^+$ calcd for $C_{14}H_{19}O_2$: 219.1380; found: 219.1381.

4. ^{1}H and ^{13}C NMR spectra of products

methyl 2-(4-oxochroman-3-yl)acetate **(3aa)**

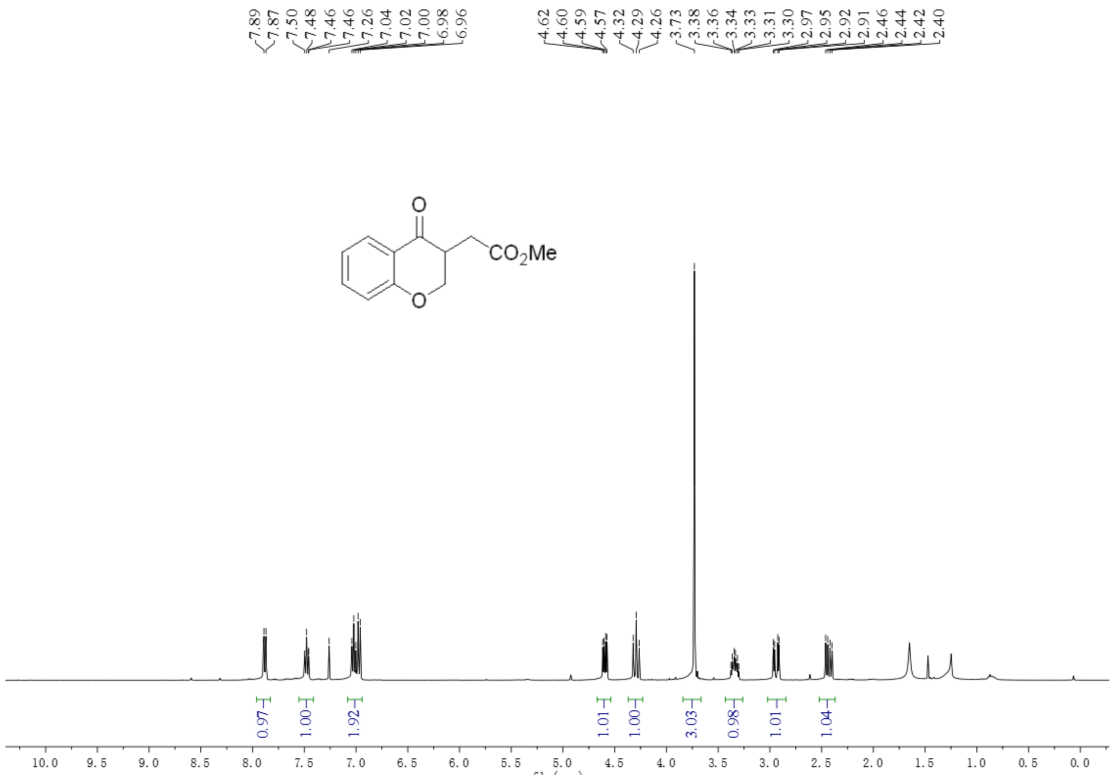

^{1}H spectra of **3aa**

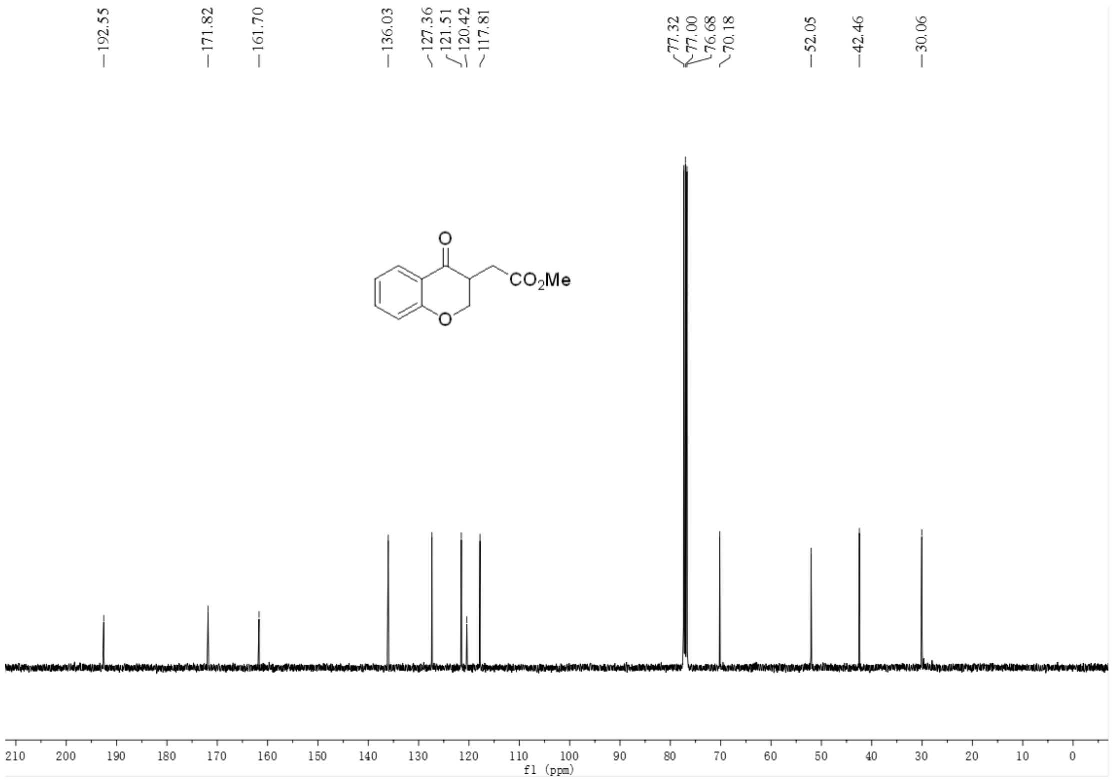

^{13}C spectra of **3aa**

methyl 2-(8-methyl-4-oxochroman-3-yl)acetate **(3ba)**

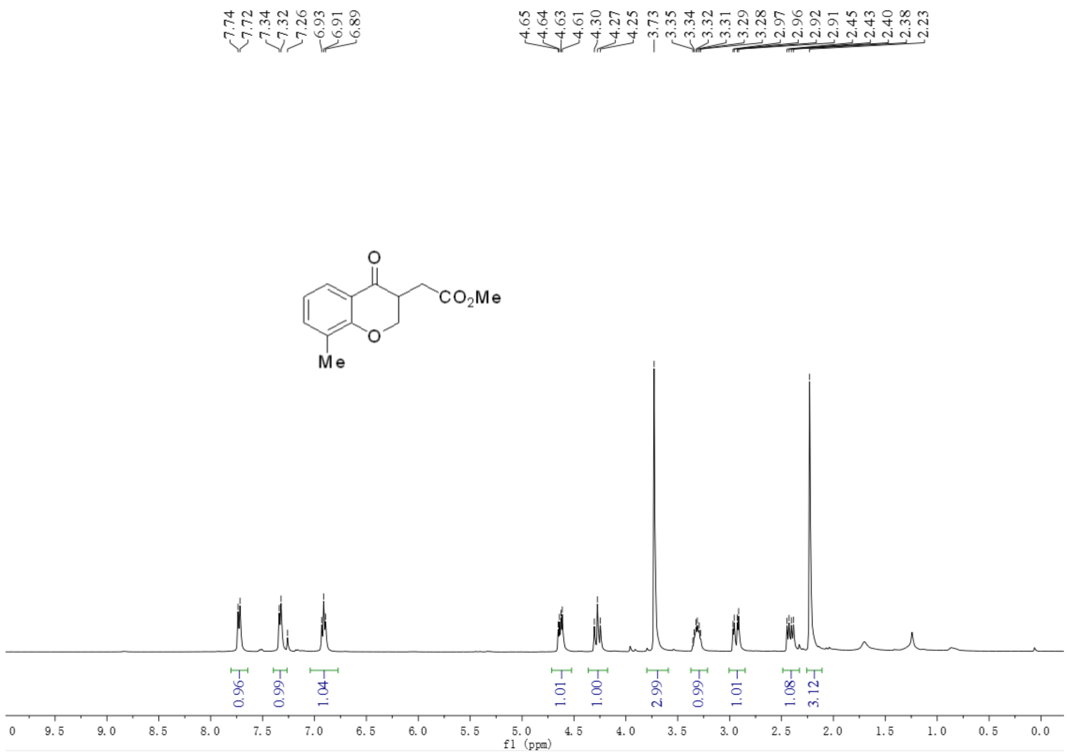

^{1}H spectra of **3ba**

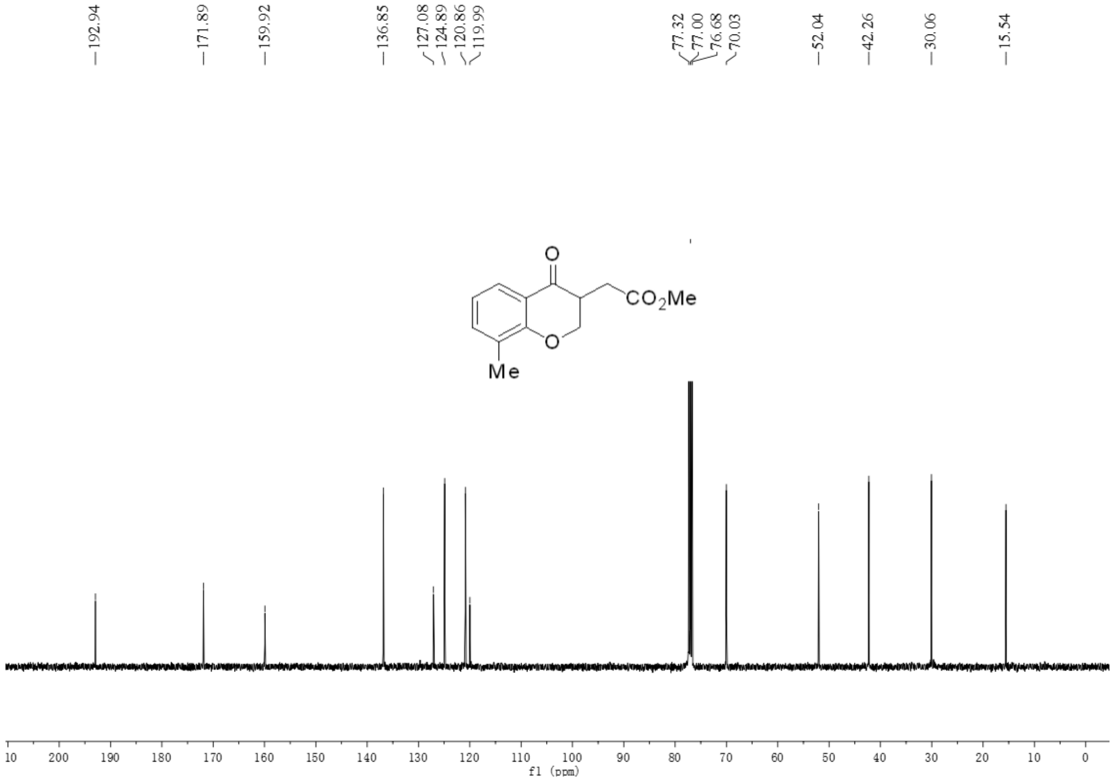

^{13}C spectra of **3ba**

methyl 2-(6-methyl-4-oxochroman-3-yl)acetate **(3ca)**

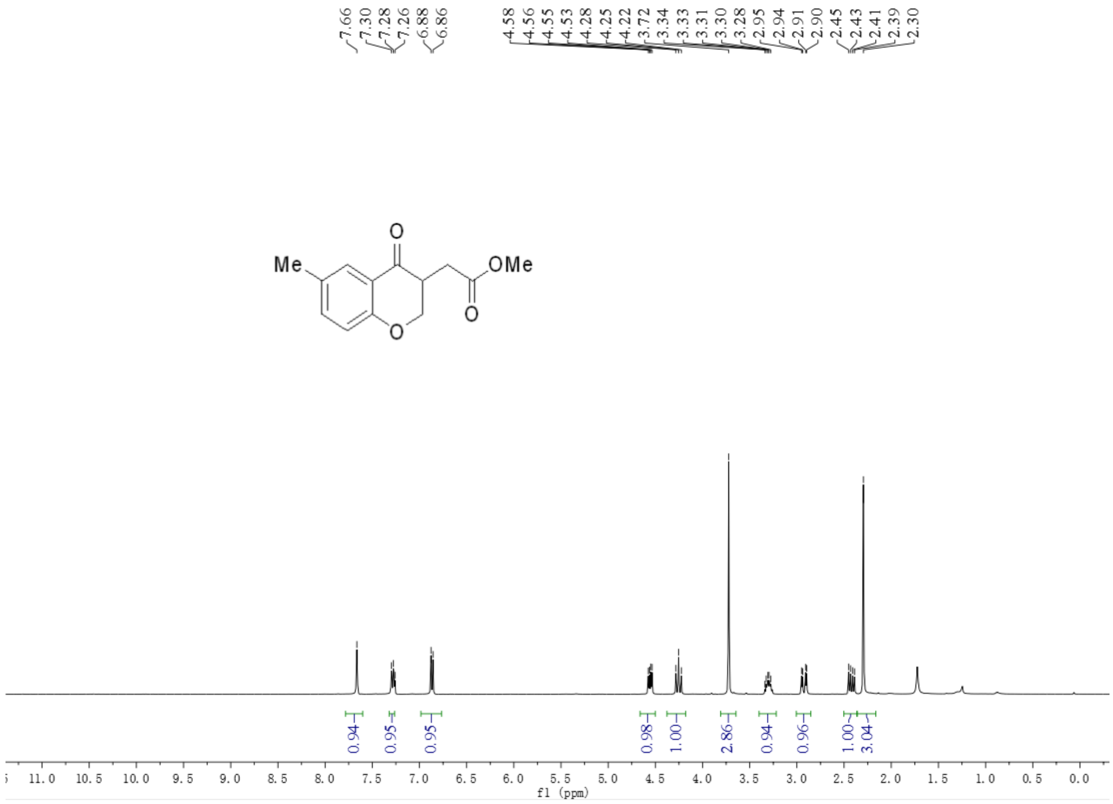

^{1}H spectra of **3ca**

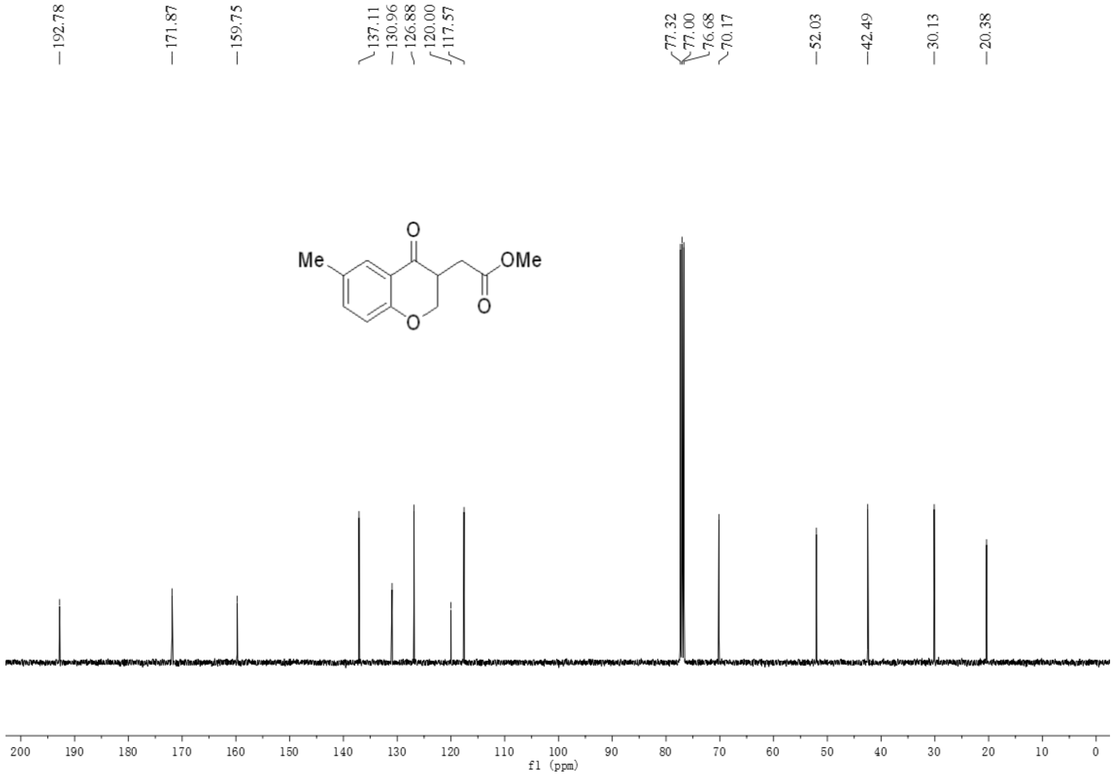

^{13}C spectra of **3ca**

methyl 2-(7-methoxy-4-oxochroman-3-yl)acetate **(3da)**

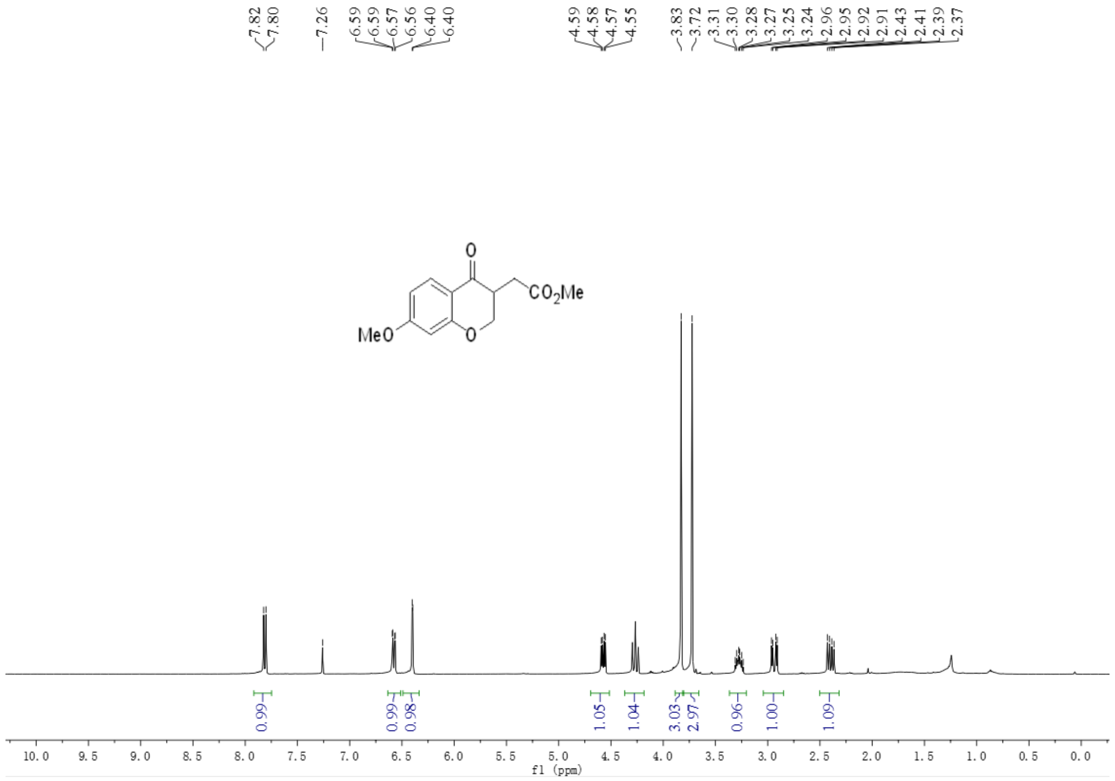

^{1}H spectra of **3da**

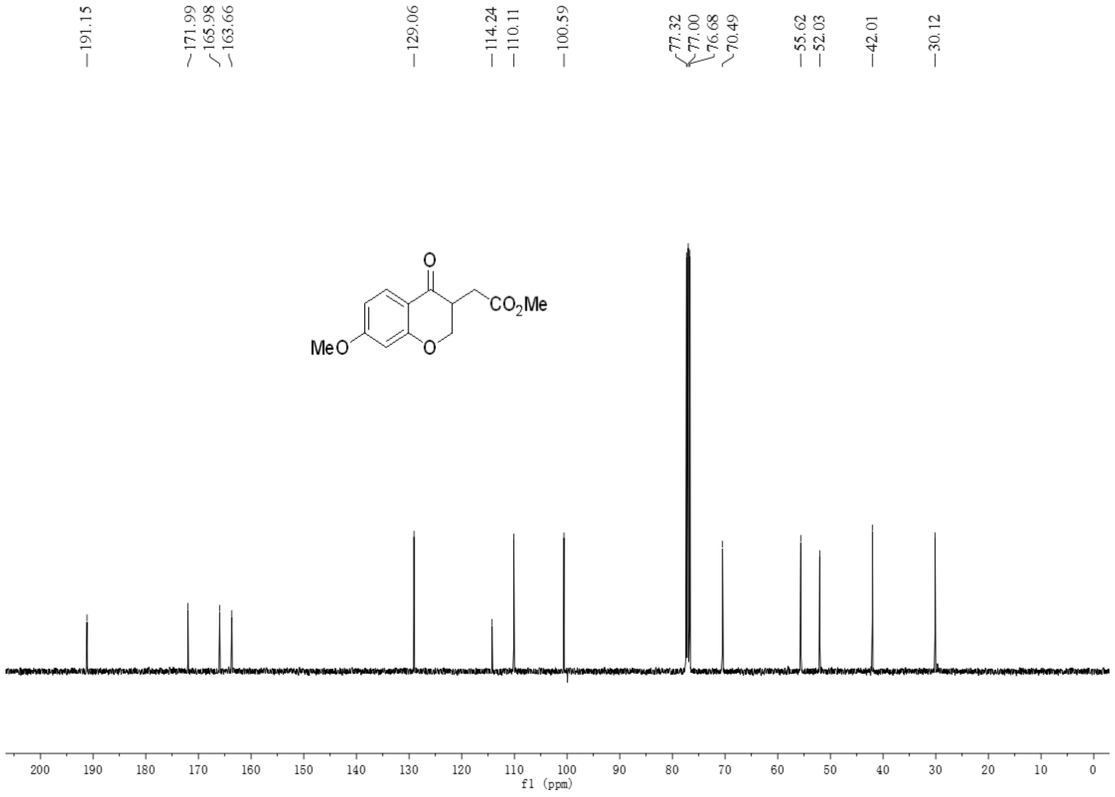

^{13}C spectra of **3da**

methyl 2-(8-(tert-butyl)-4-oxochroman-3-yl)acetate **(3ea)**

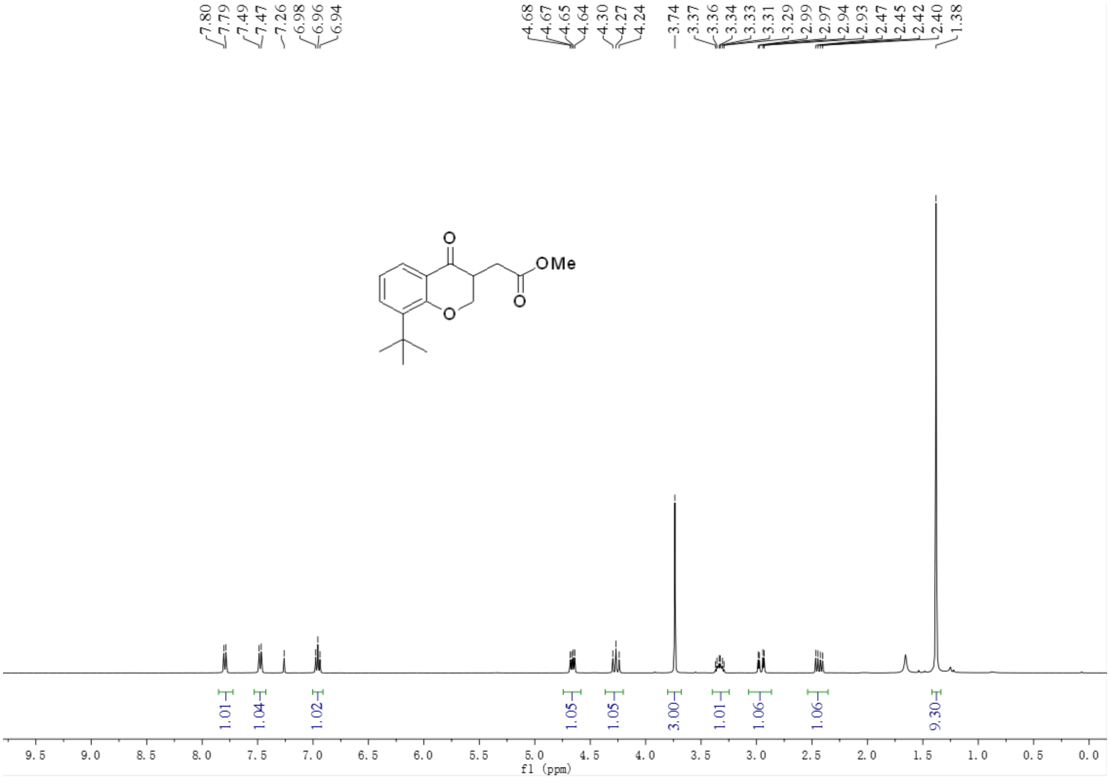

^{1}H spectra of **3ea**

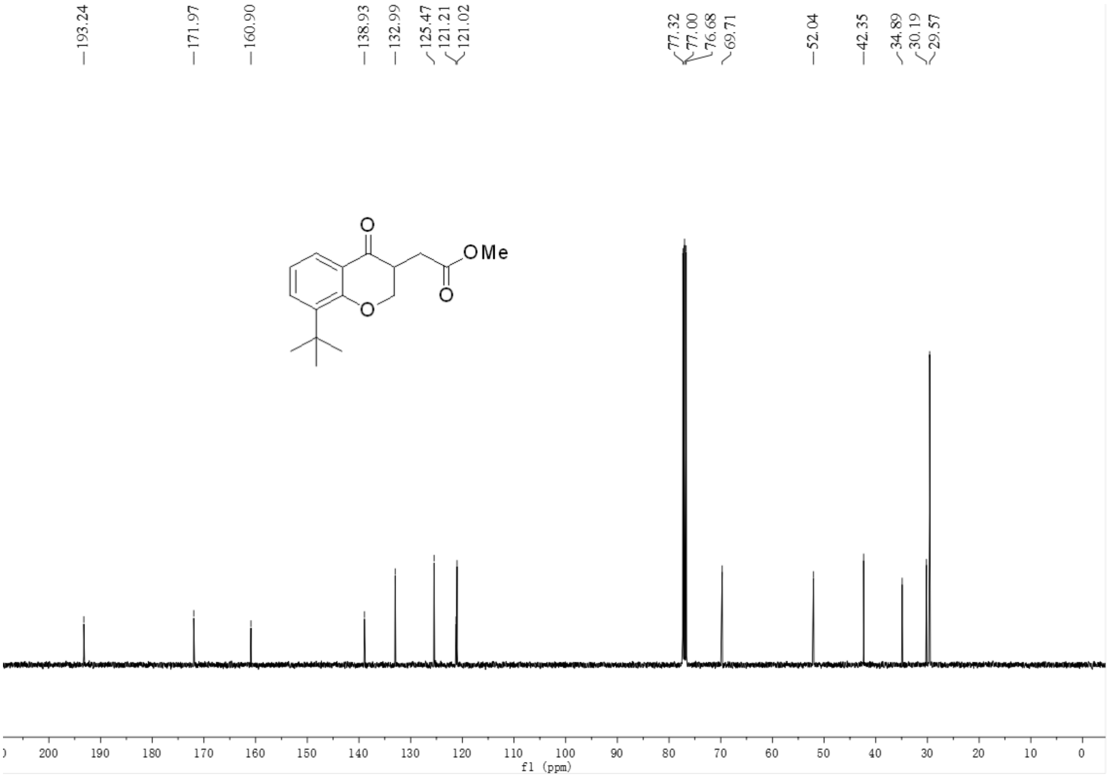

^{13}C spectra of **3ea**

methyl 2-(7-fluoro-4-oxochroman-3-yl)acetate (**3fa**)

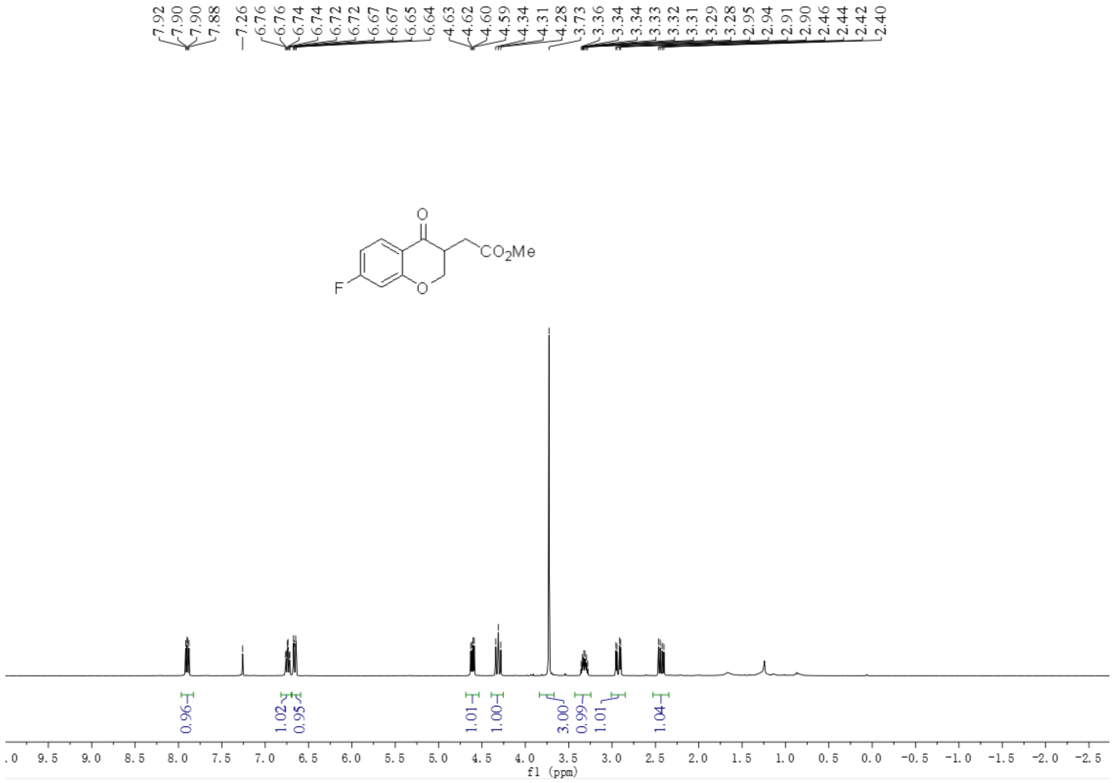

^{1}H spectra of **3fa**

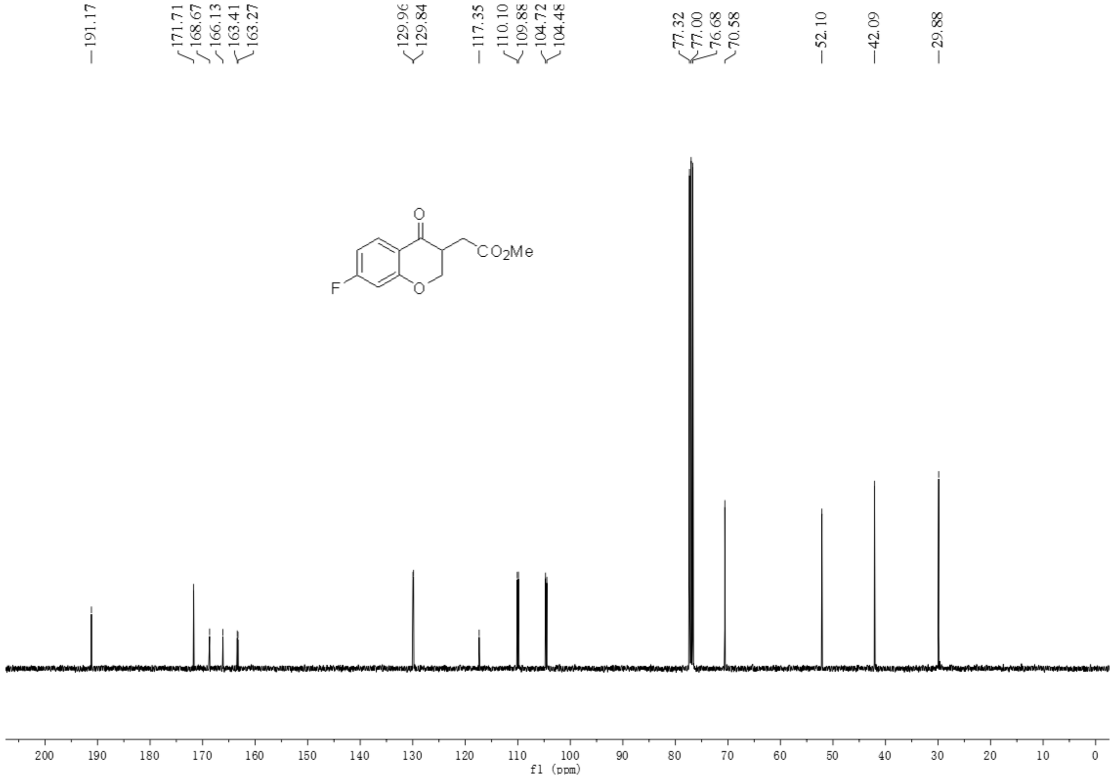

^{13}C spectra of **3fa**

methyl 2-(6-fluoro-4-oxochroman-3-yl)acetate **(3ga)**

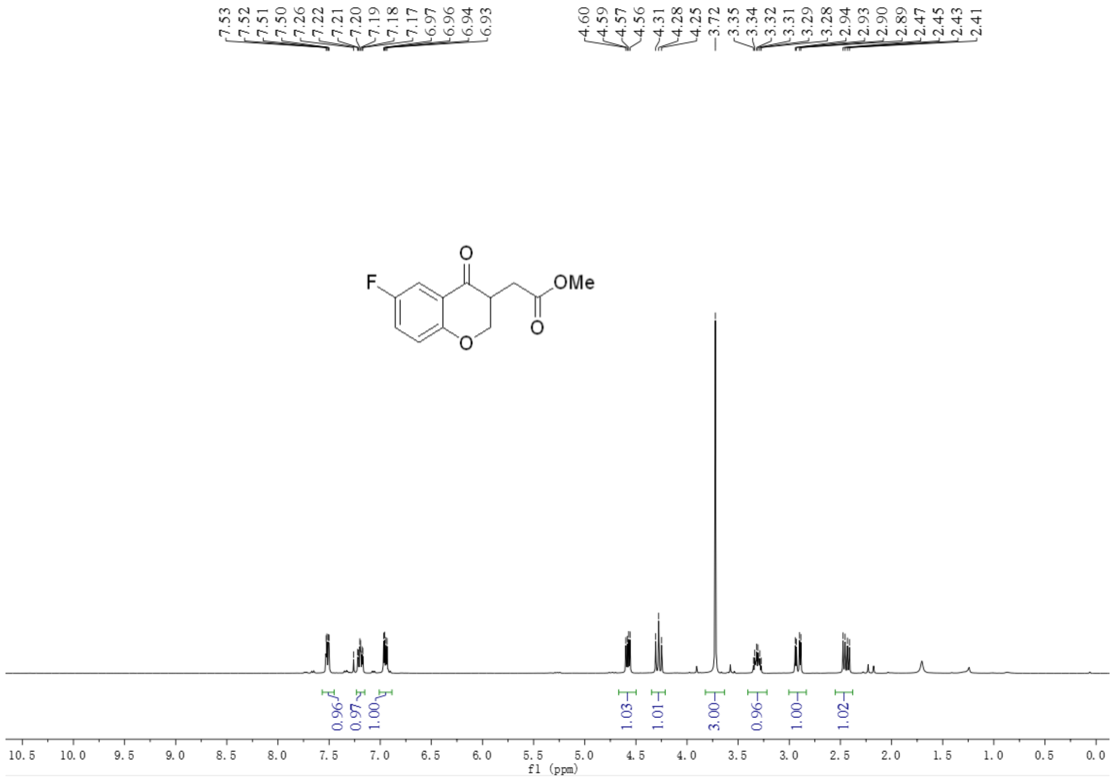

^{1}H spectra of **3ga**

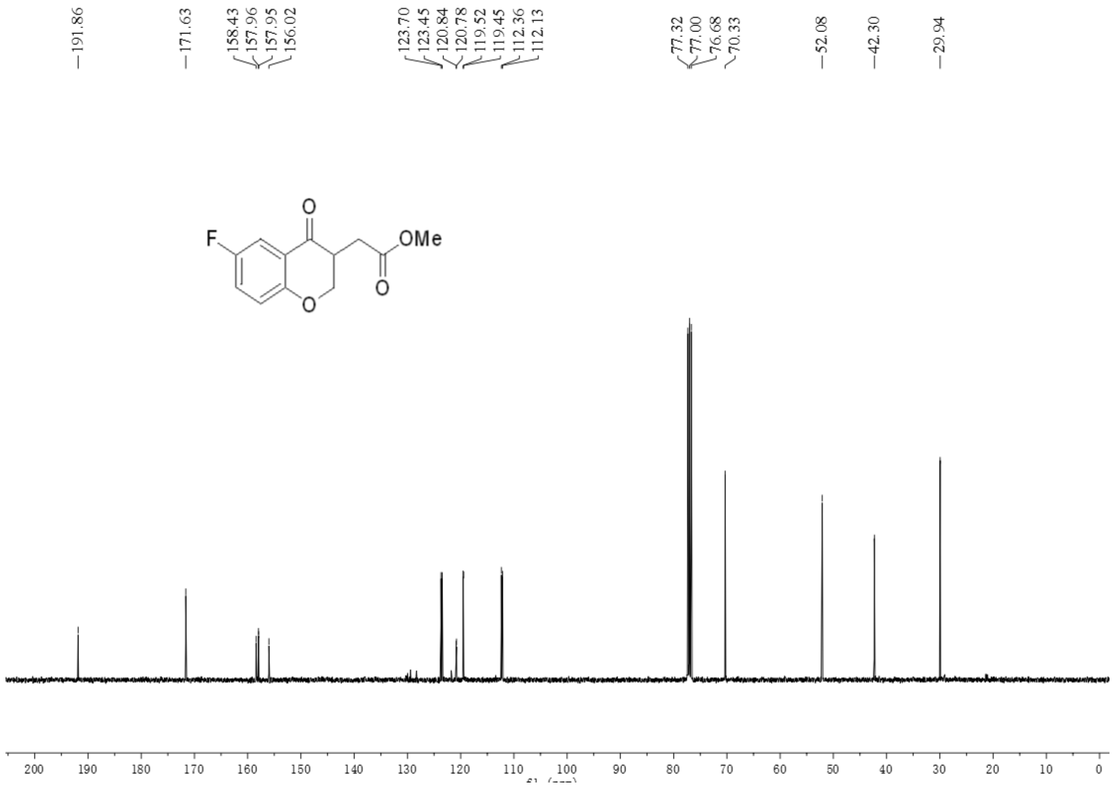

^{13}C spectra of **3ga**

methyl 2-(8-chloro-4-oxochroman-3-yl)acetate **(3ha)**

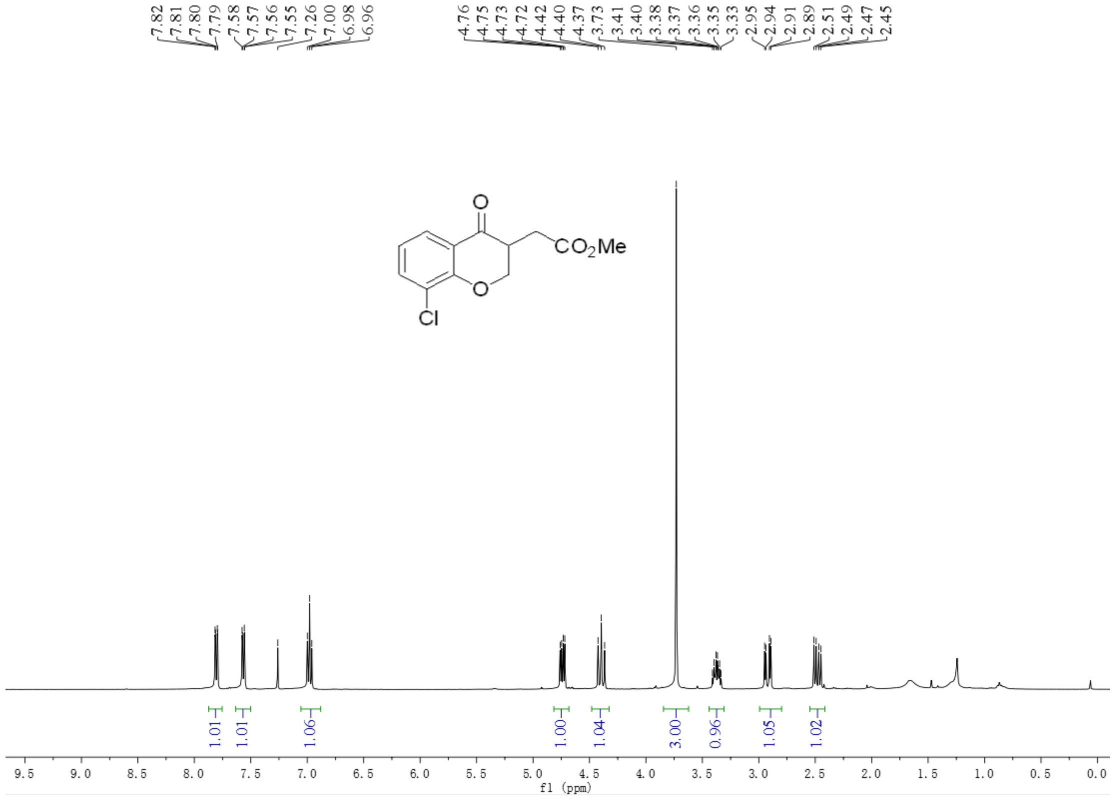

^{1}H spectra of **3ha**

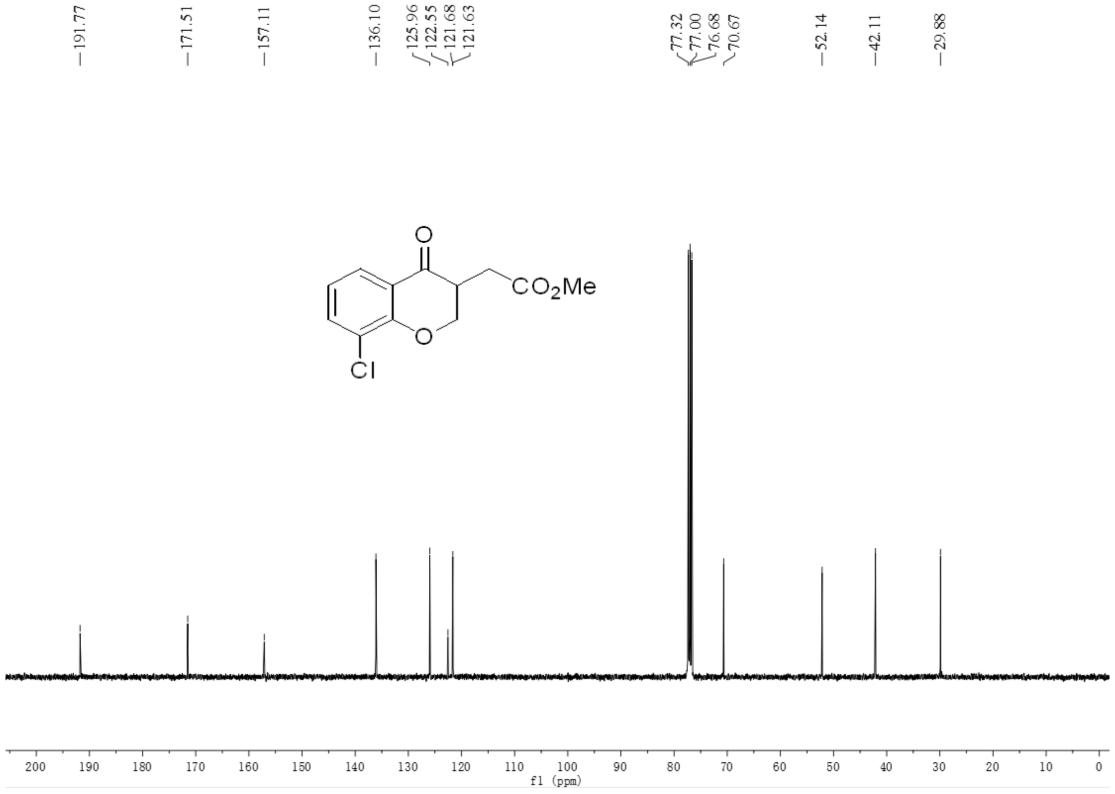

^{13}C spectra of **3ha**

methyl 2-(7-chloro-4-oxochroman-3-yl)acetate **(3ia)**

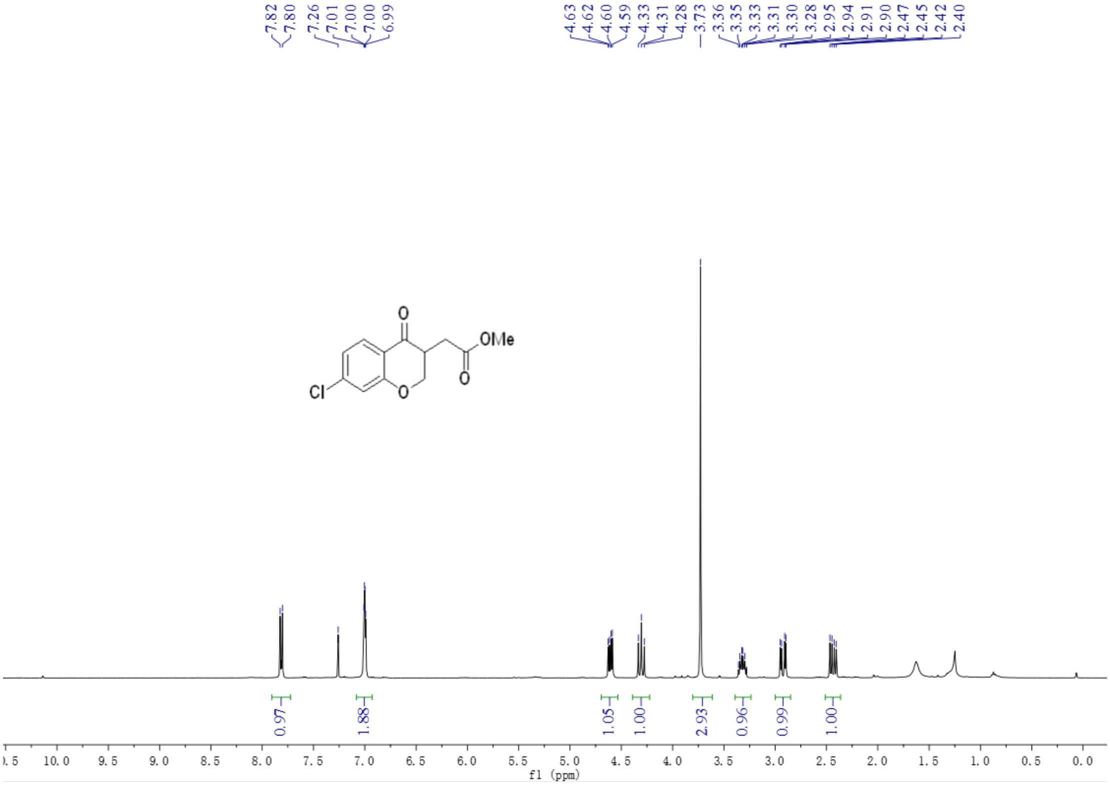

^{1}H spectra of **3ia**

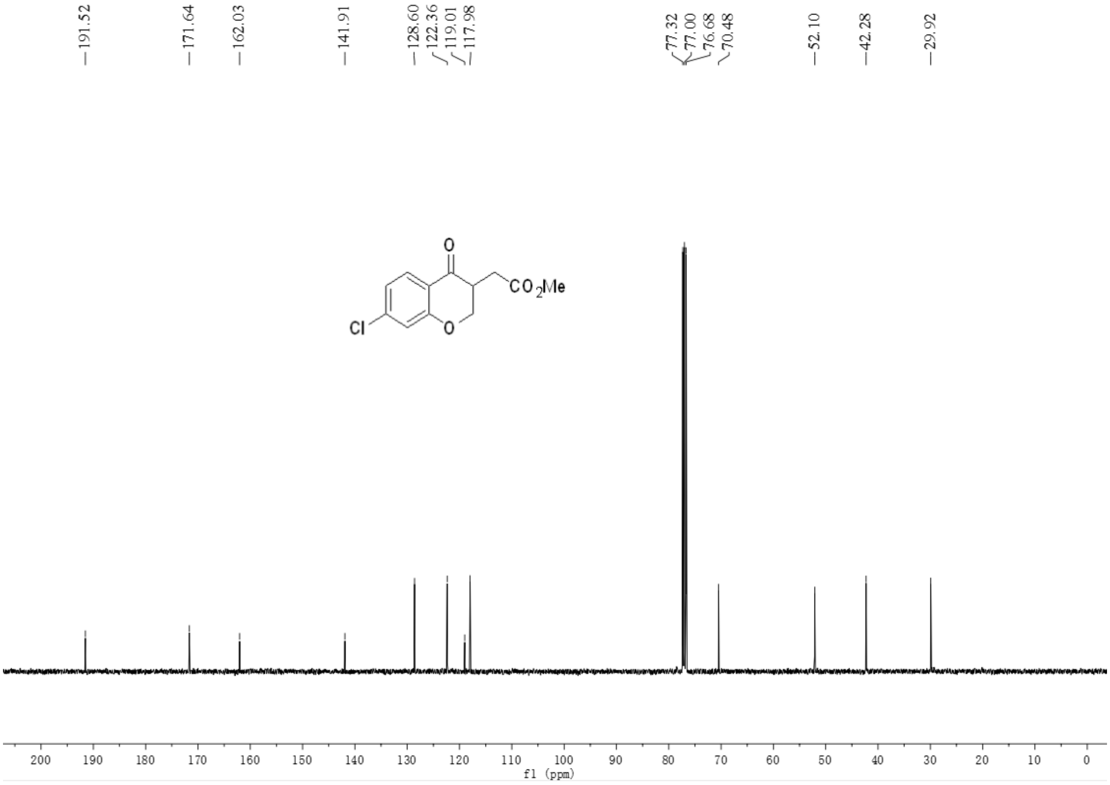

^{13}C spectra of **3ia**

methyl 2-(5-chloro-4-oxochroman-3-yl)acetate (**3ja**)

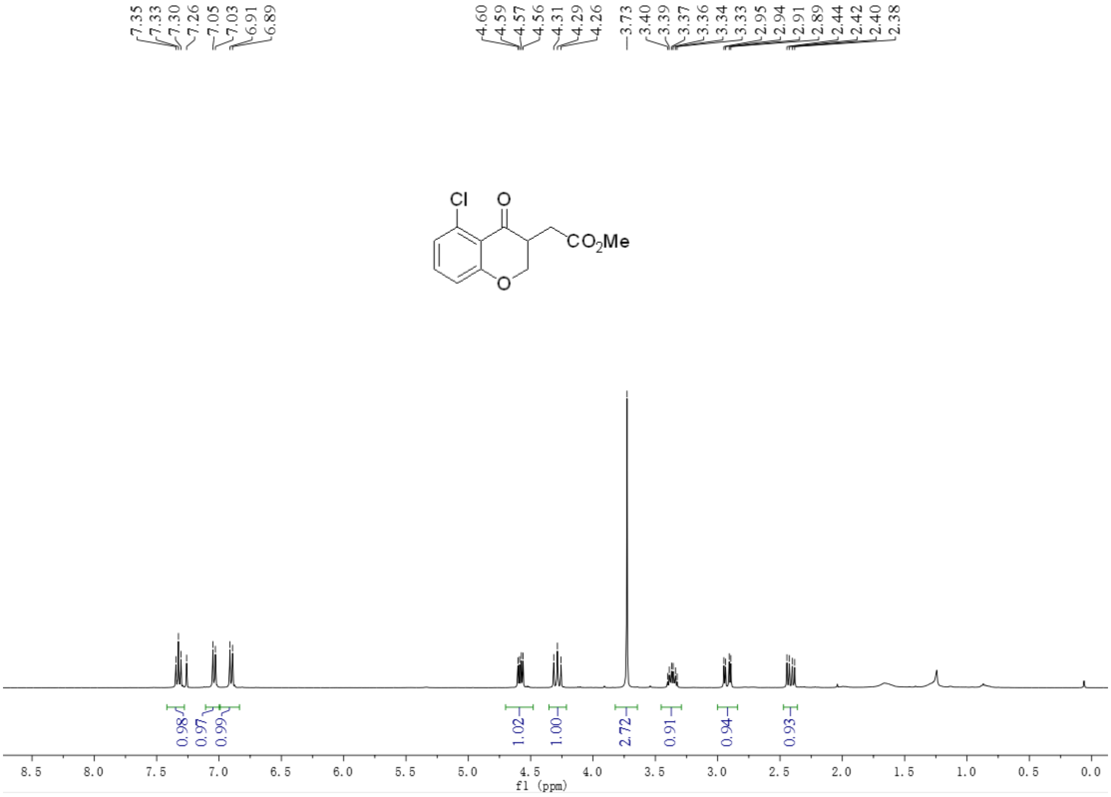

^{1}H spectra of **3ja**

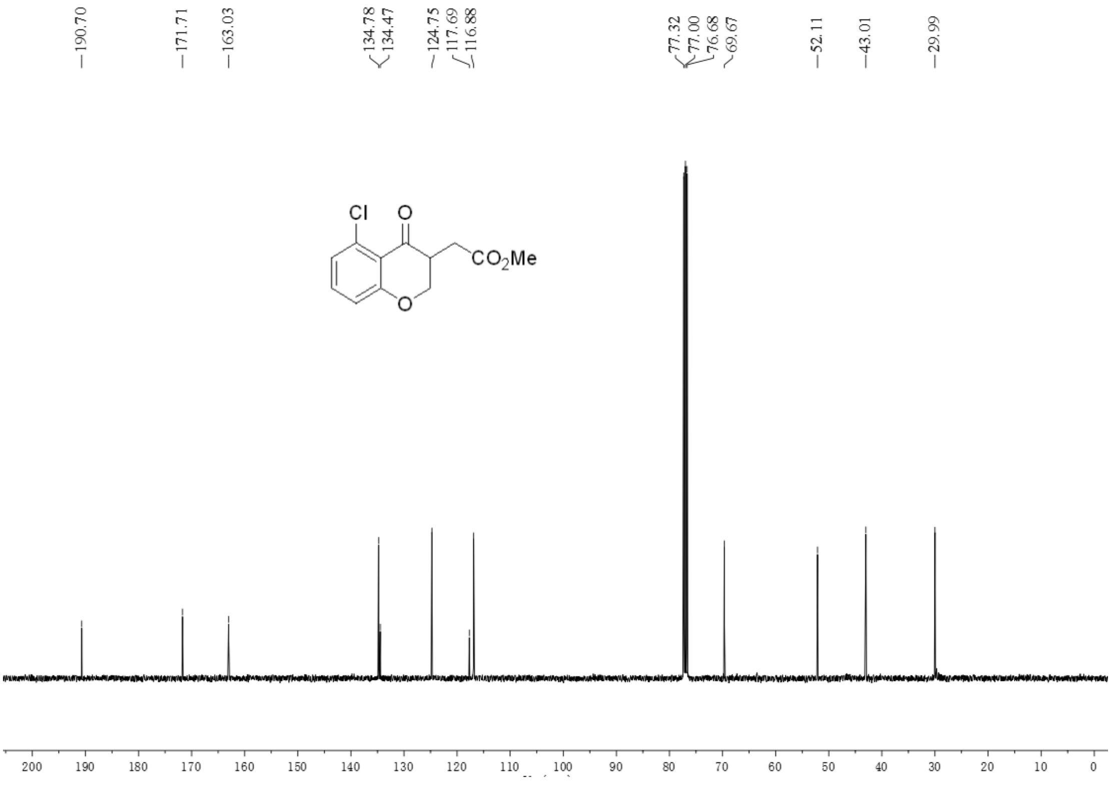

^{13}C spectra of **3ja**

methyl 2-(7-bromo-4-oxochroman-3-yl)acetate **(3ka)**

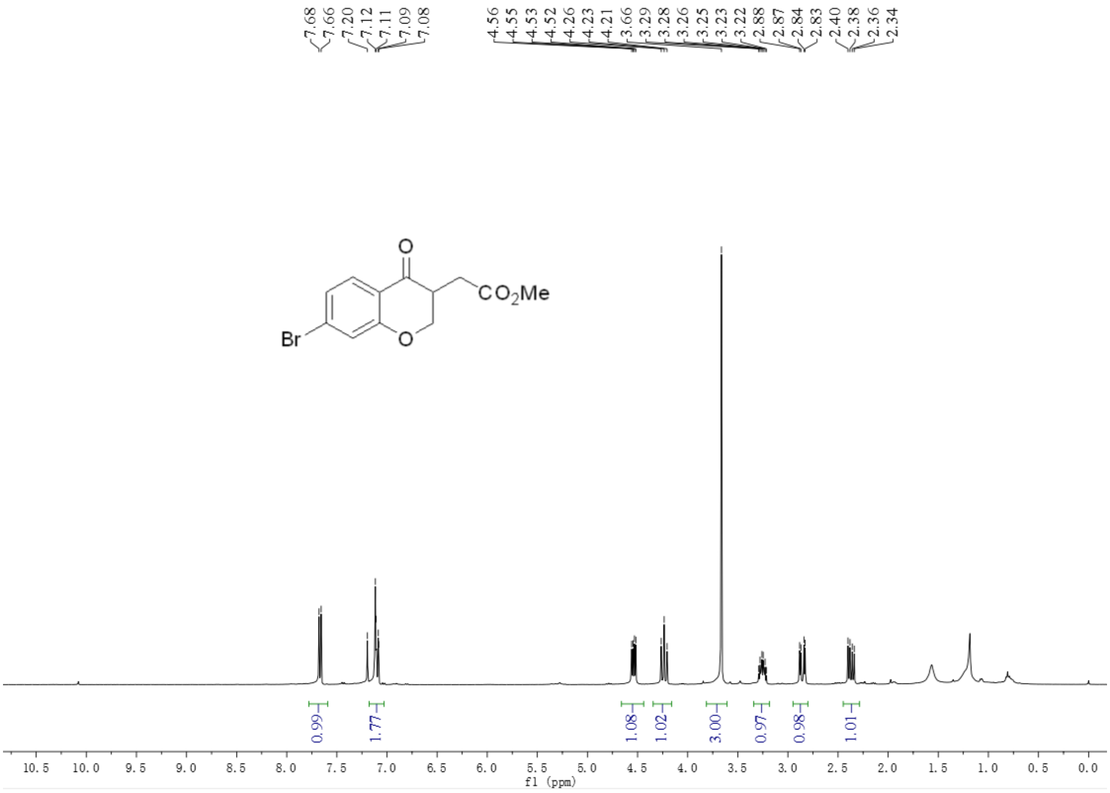

^{1}H spectra of **3ka**

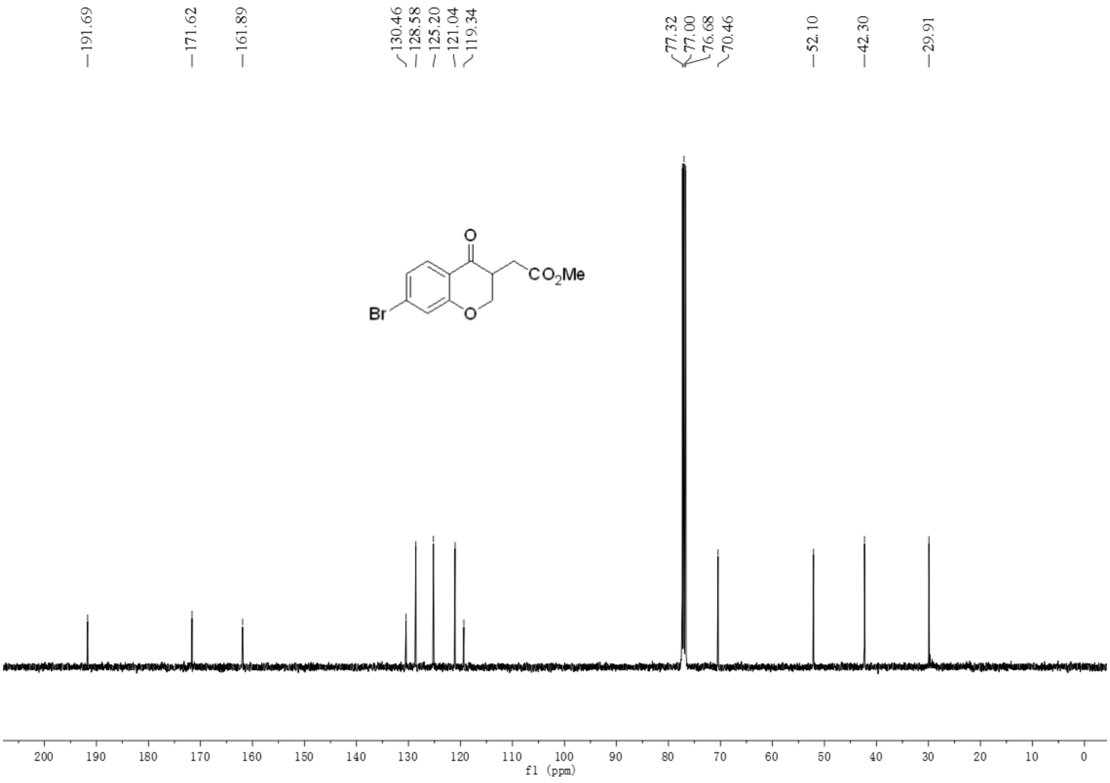

^{13}C spectra of **3ka**

methyl 2-(6-bromo-4-oxochroman-3-yl)acetate (**3la**)

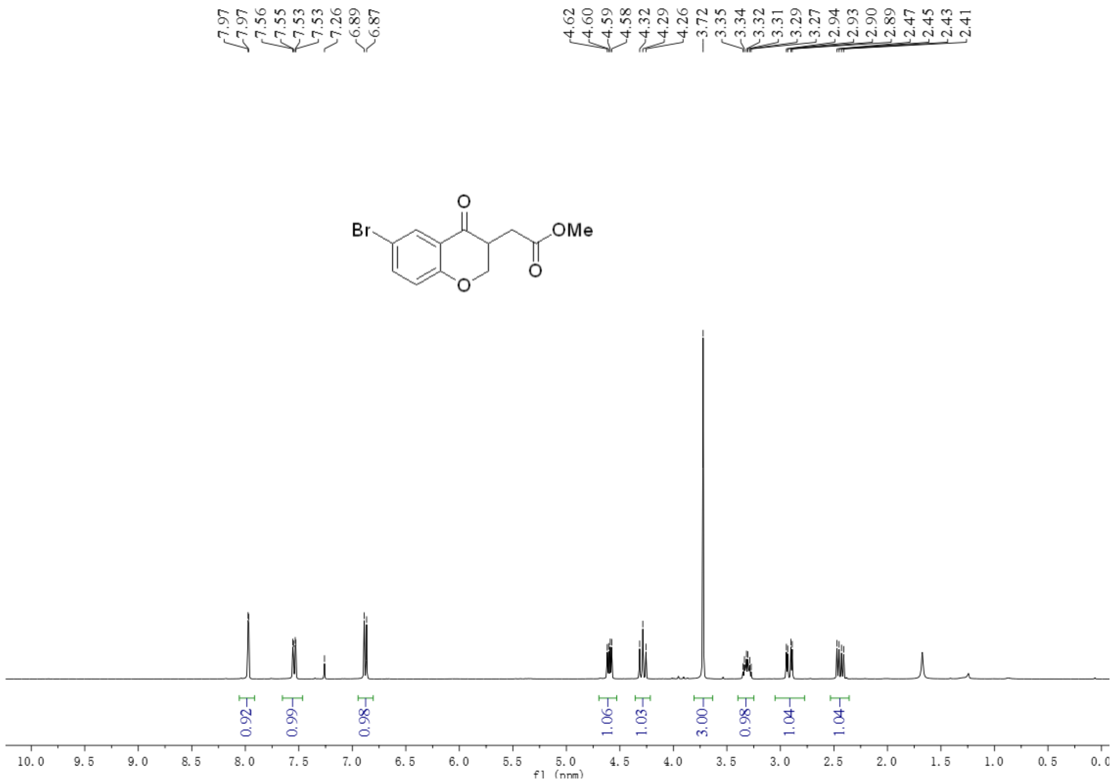

^{1}H spectra of **3la**

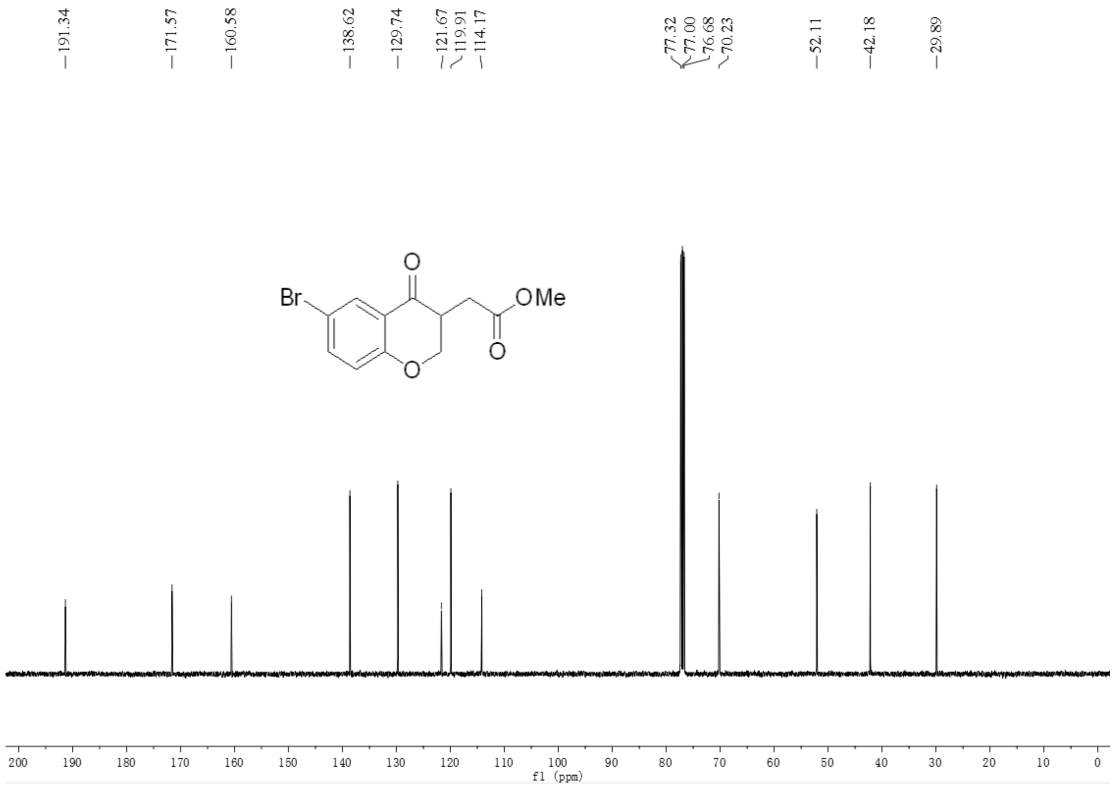

^{13}C spectra of **3la**

methyl 3-(2-methoxy-2-oxoethyl)-4-oxochromane-6-carboxylate **(3ma)**

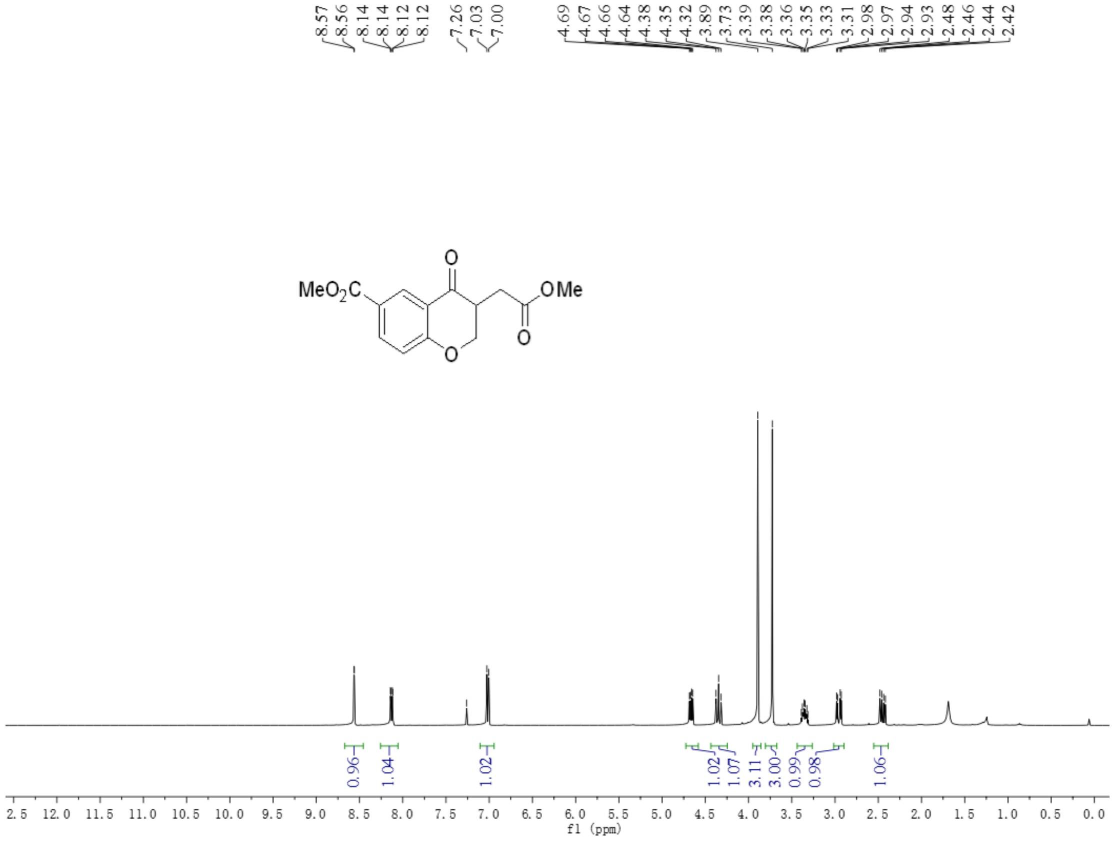

^{1}H spectra of **3ma**

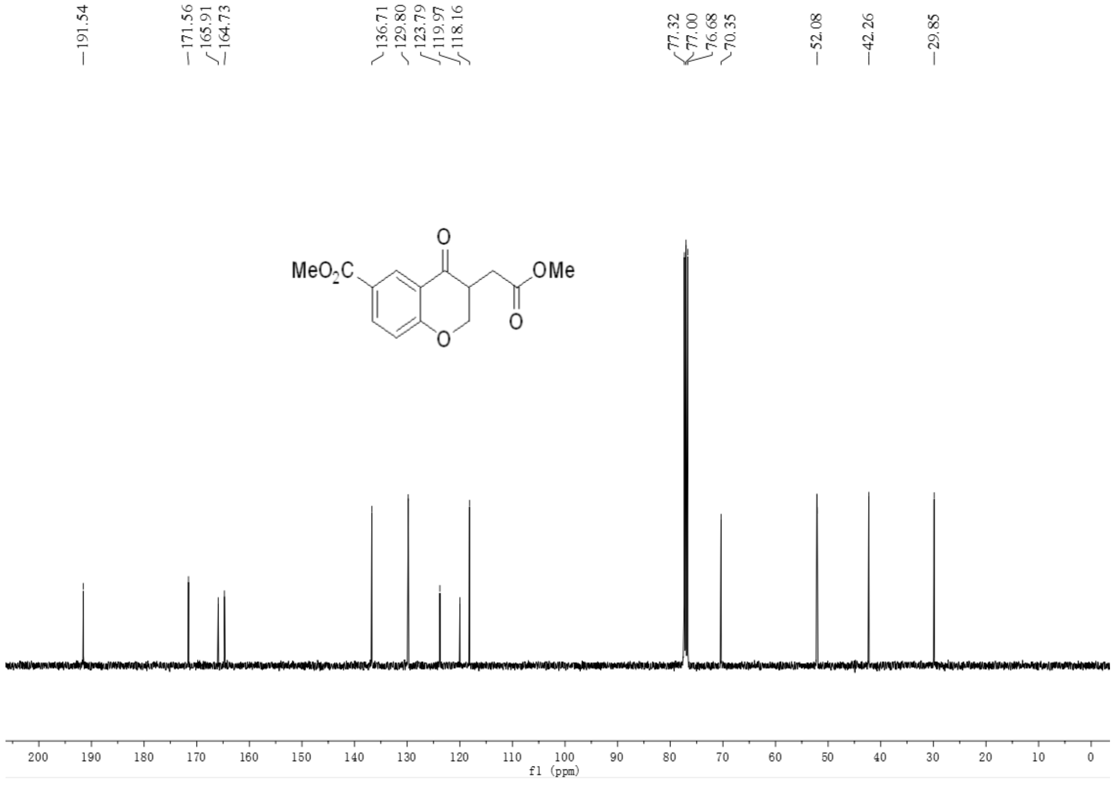

^{13}C spectra of **3ma**

***methyl 2-(6-nitro-4-oxochroman-3-yl)acetate* (3na)**

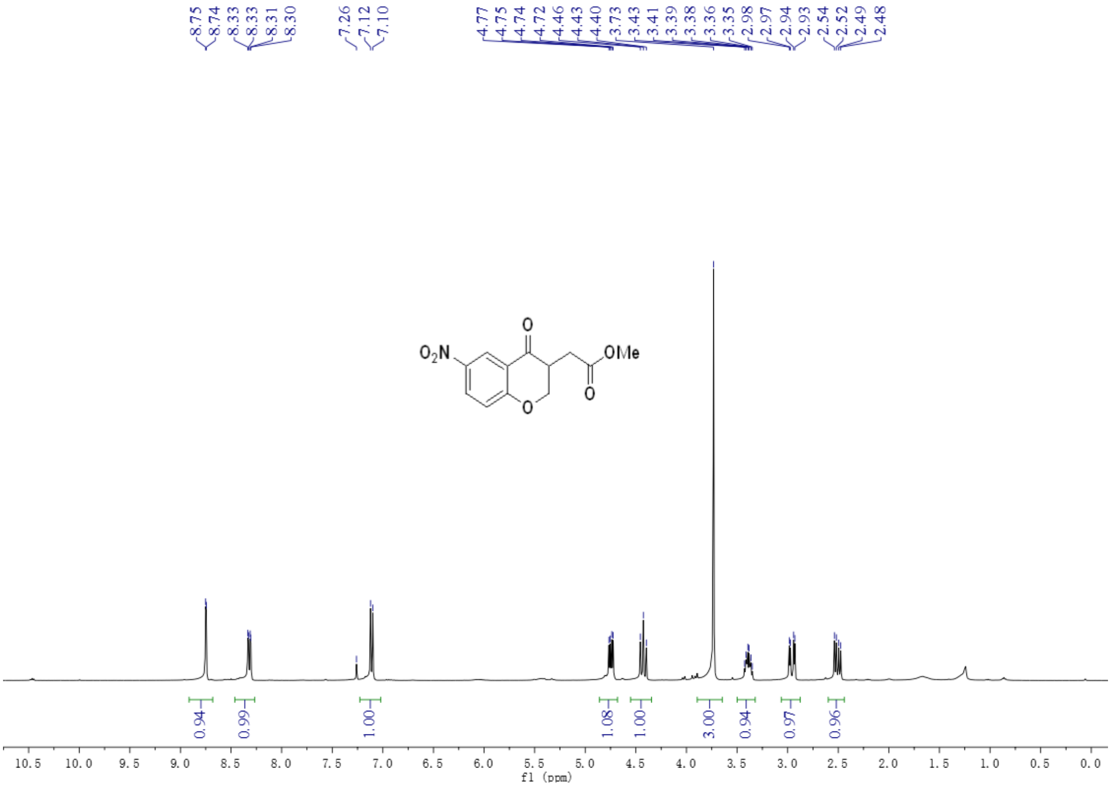

^{1}H spectra of **3na**

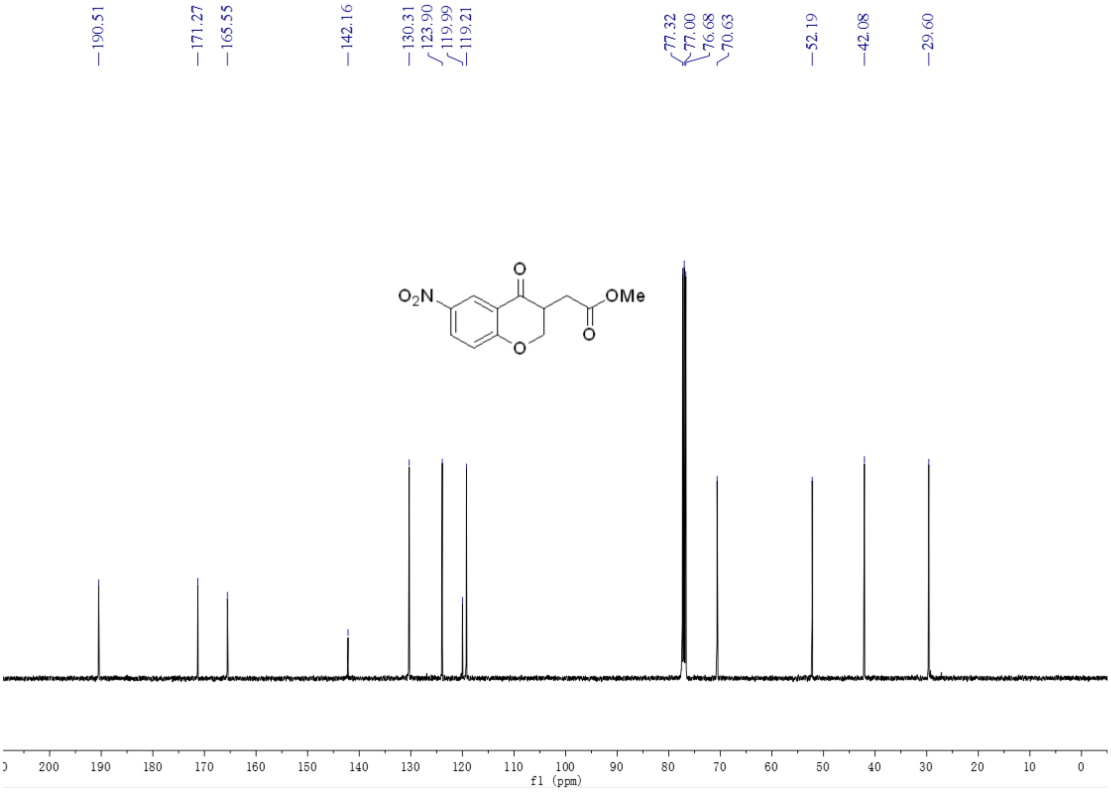

^{13}C spectra of **3na**

methyl 2-(7-(benzyloxy)-4-oxochroman-3-yl)acetate **(3oa)**

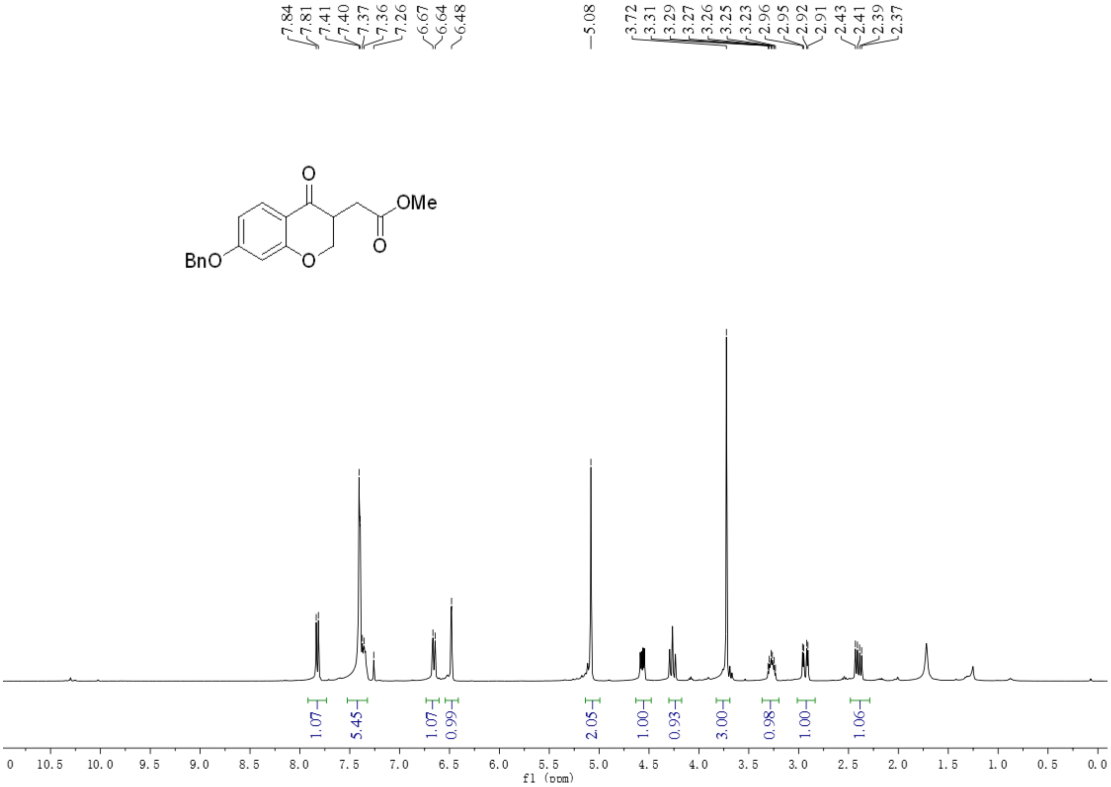

^{1}H spectra of **3oa**

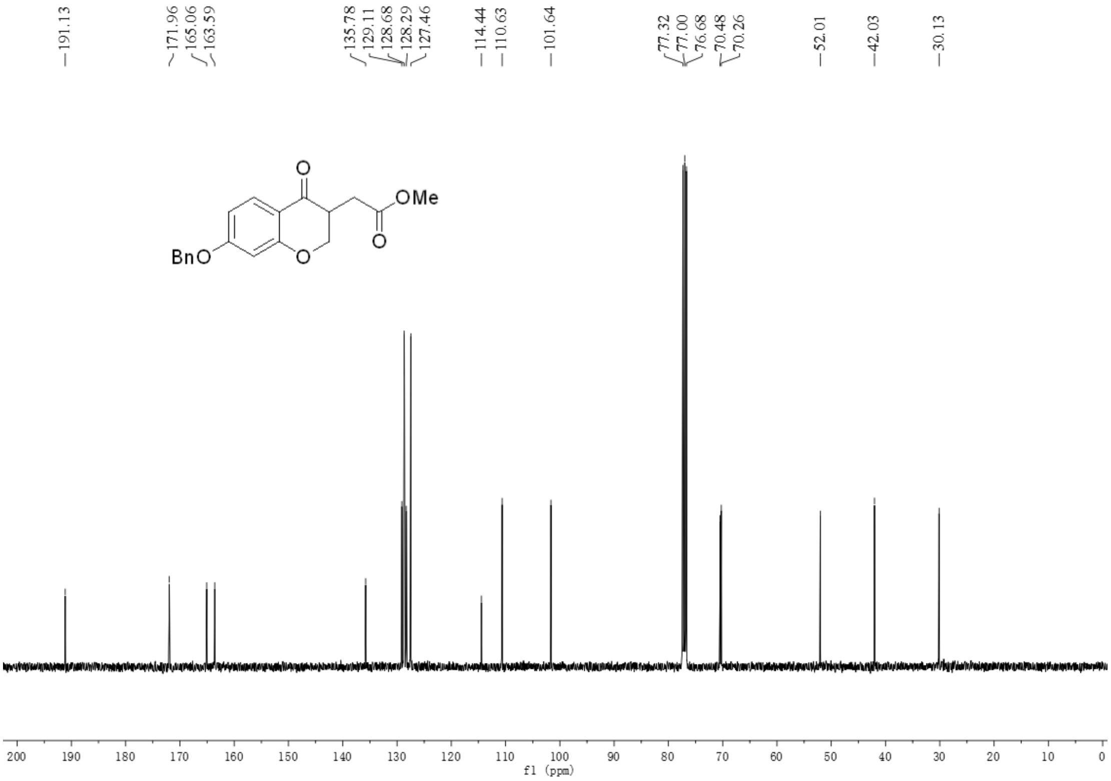

^{13}C spectra of **3oa**

methyl 2-(6,8-dichloro-4-oxochroman-3-yl)acetate (**3pa**)

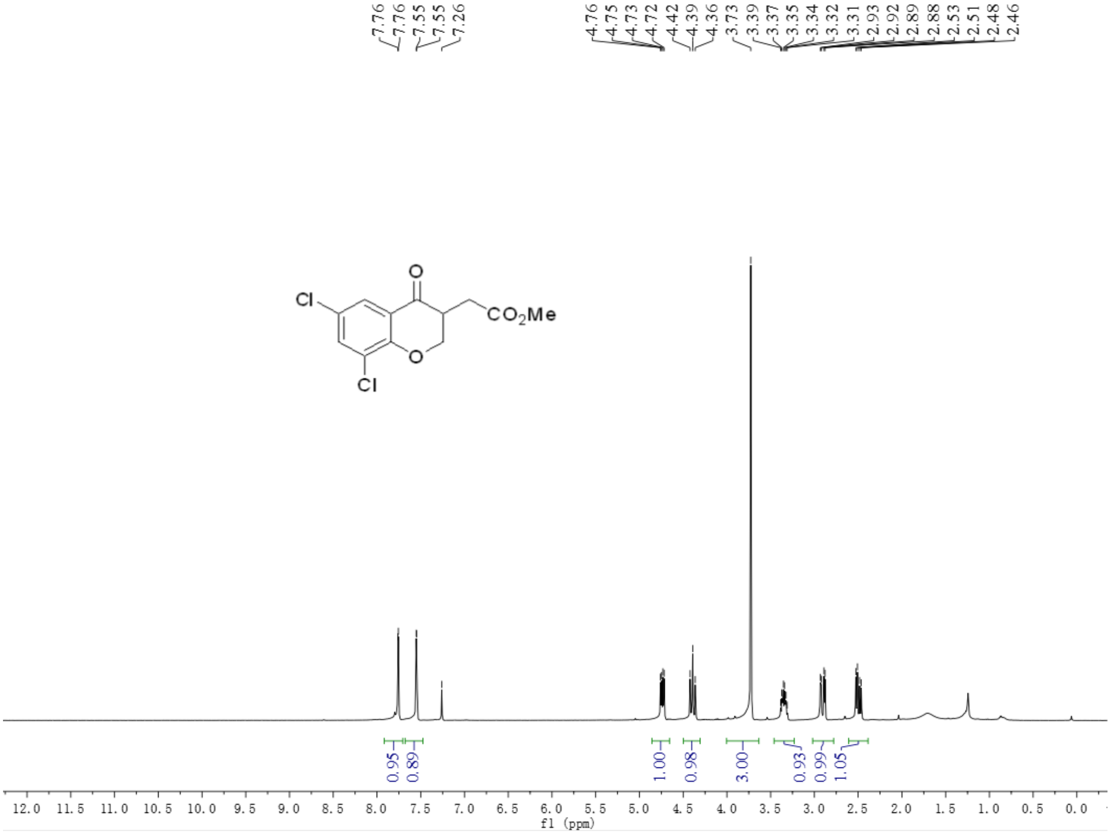

^{1}H spectra of **3pa**

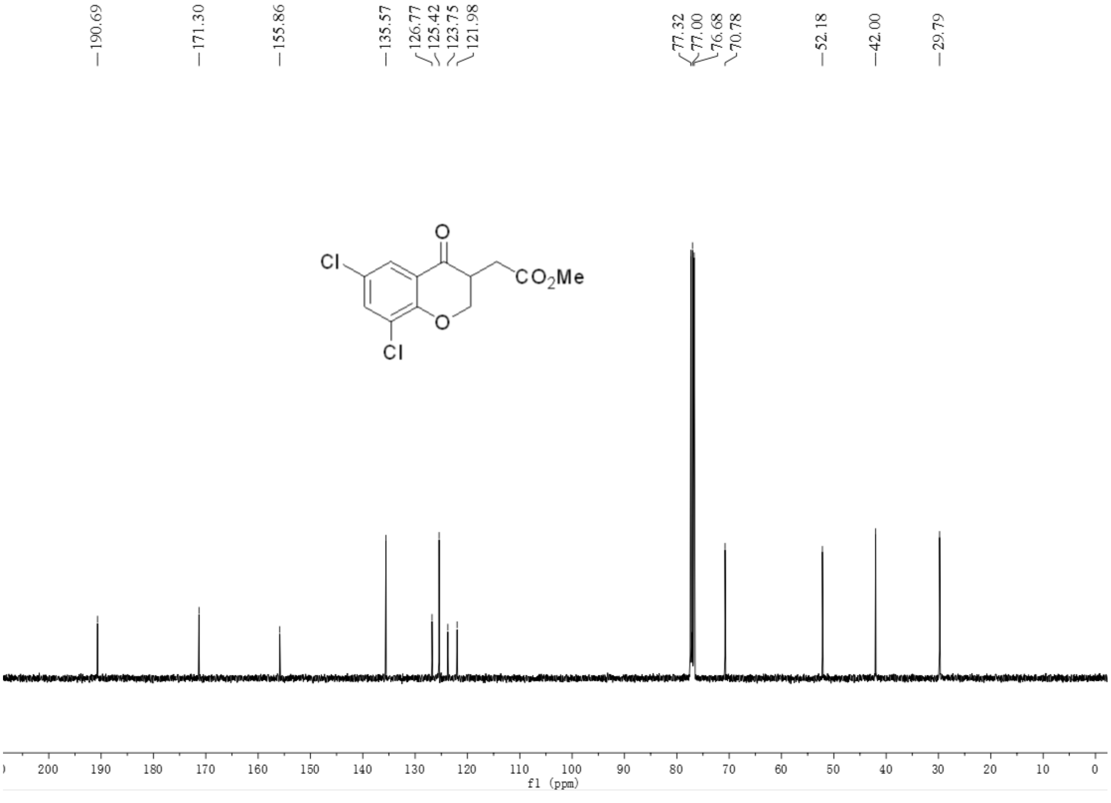

^{13}C spectra of **3pa**

methyl 2-(4-oxo-3,4-dihydro-2H-benzo[h]chromen-3-yl)acetate **(3qa)**

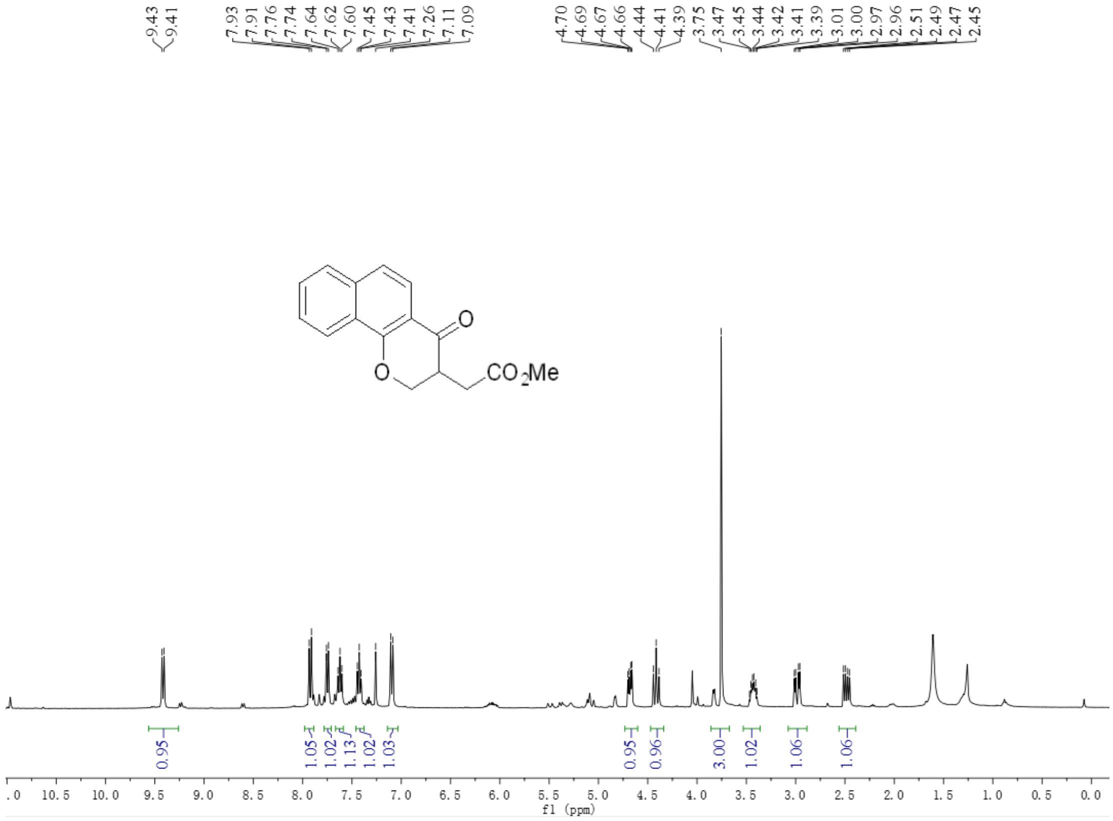

^{1}H spectra of **3qa**

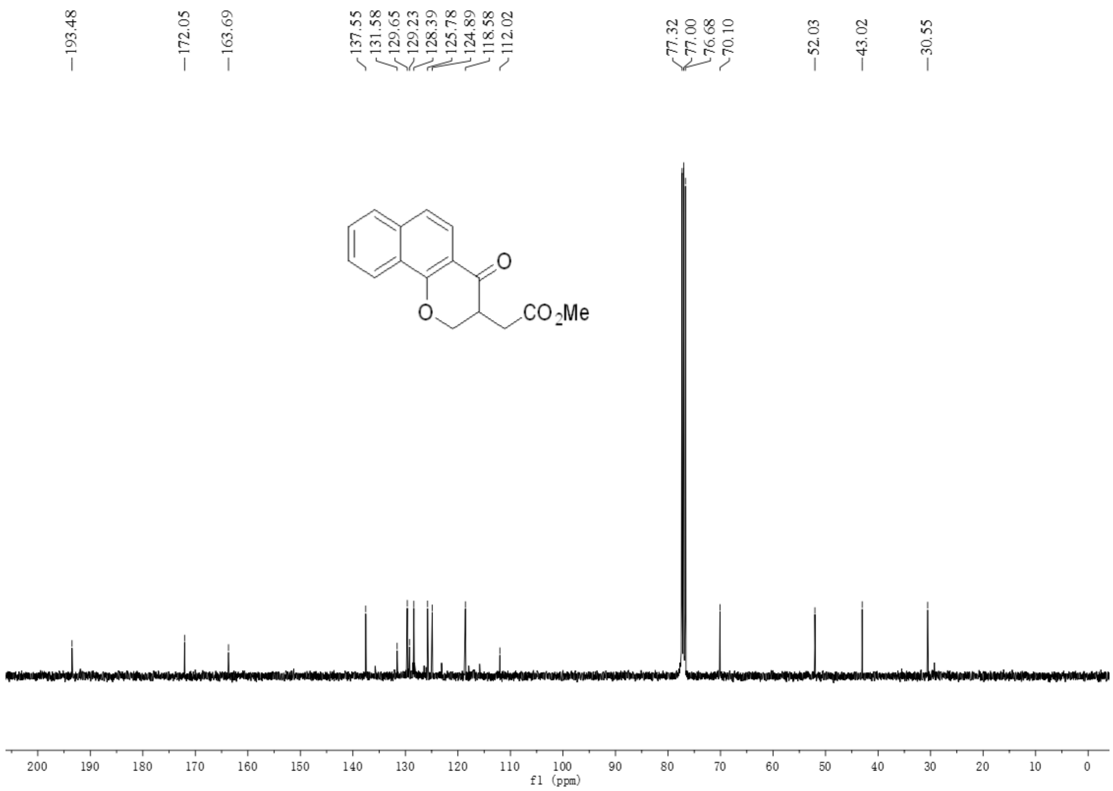

^{13}C spectra of **3qa**

methyl 2-(3-methyl-4-oxochroman-3-yl)acetate **(3ra)**

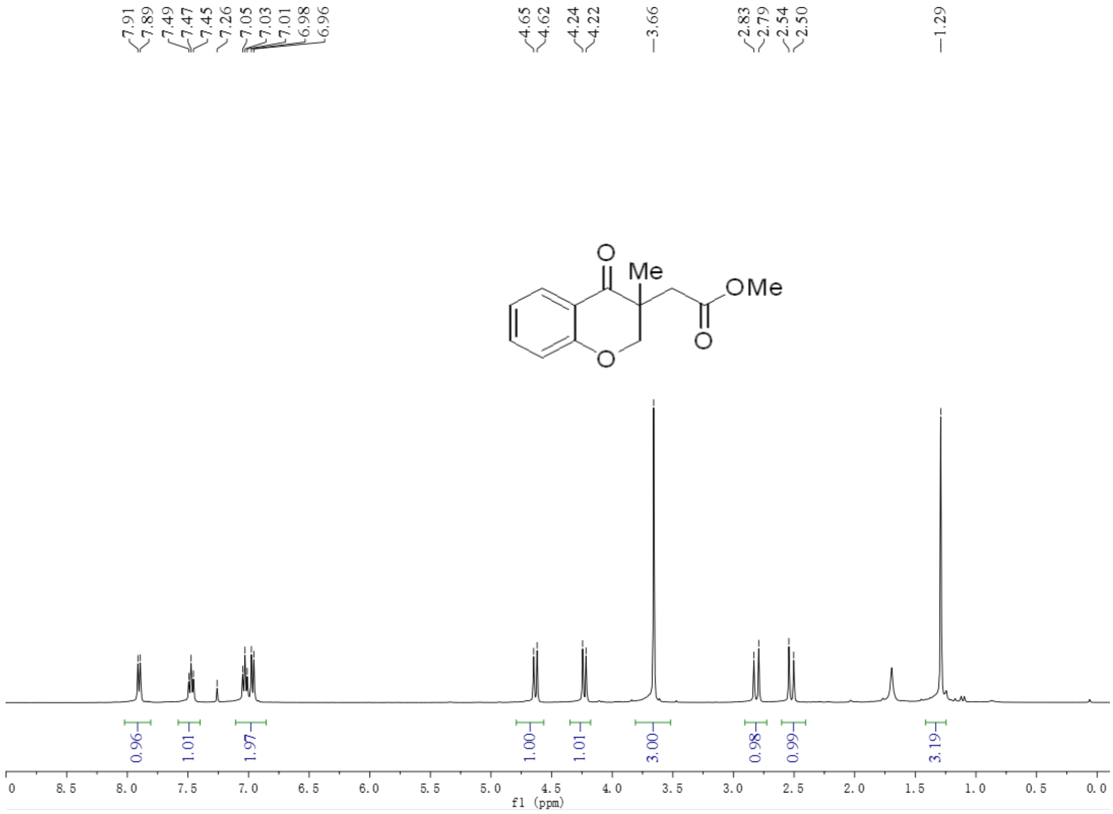

^{1}H spectra of **3ra**

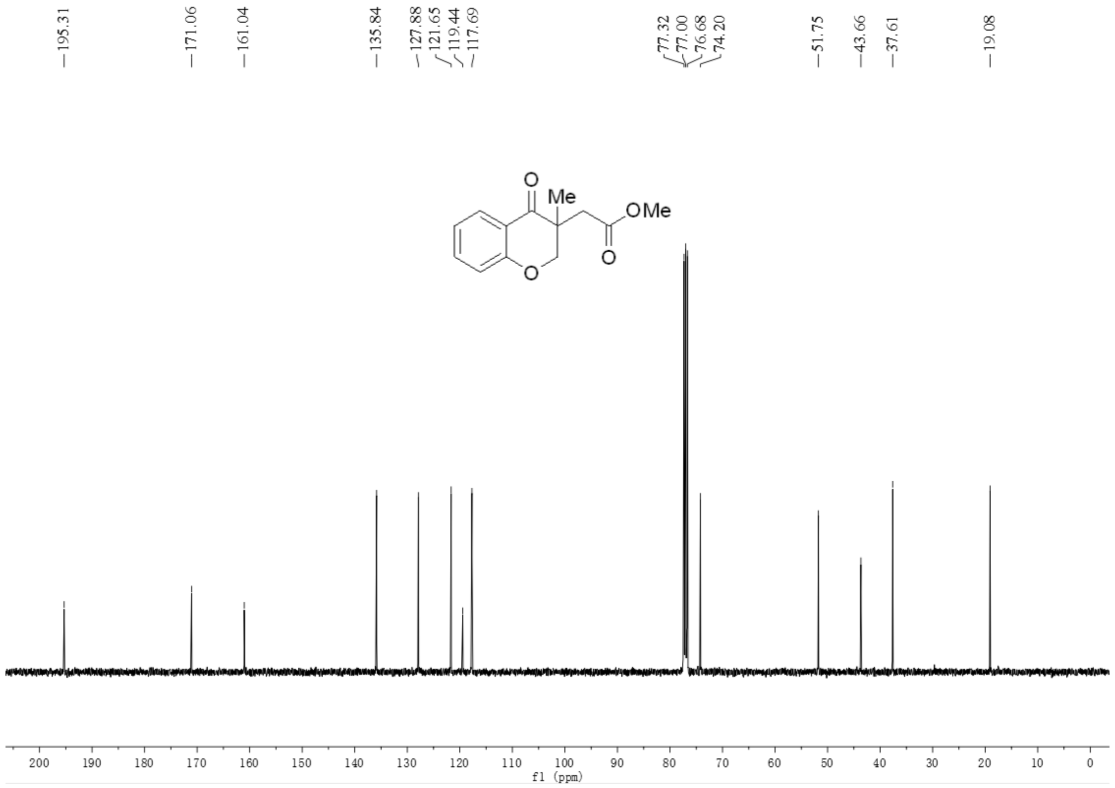

^{13}C spectra of **3ra**

methyl 2-(1-oxo-2,3-dihydro-1H-inden-2-yl)acetate (**3sa**)

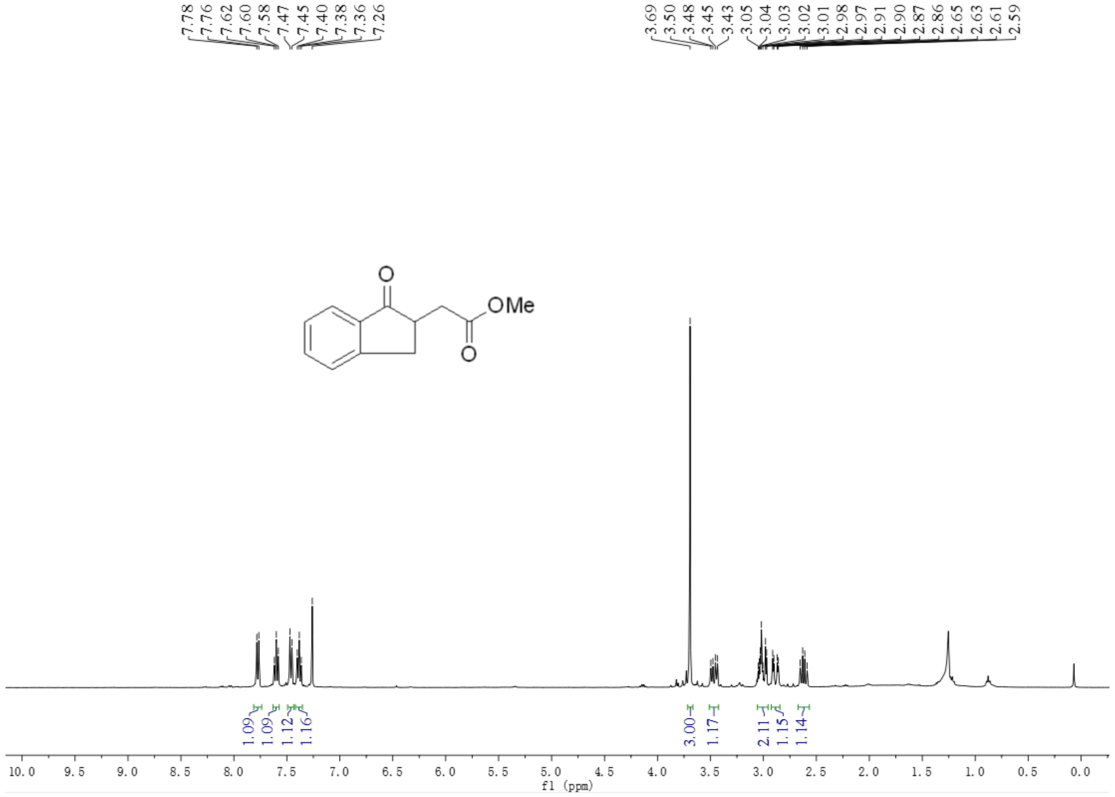

^{1}H spectra of **3sa**

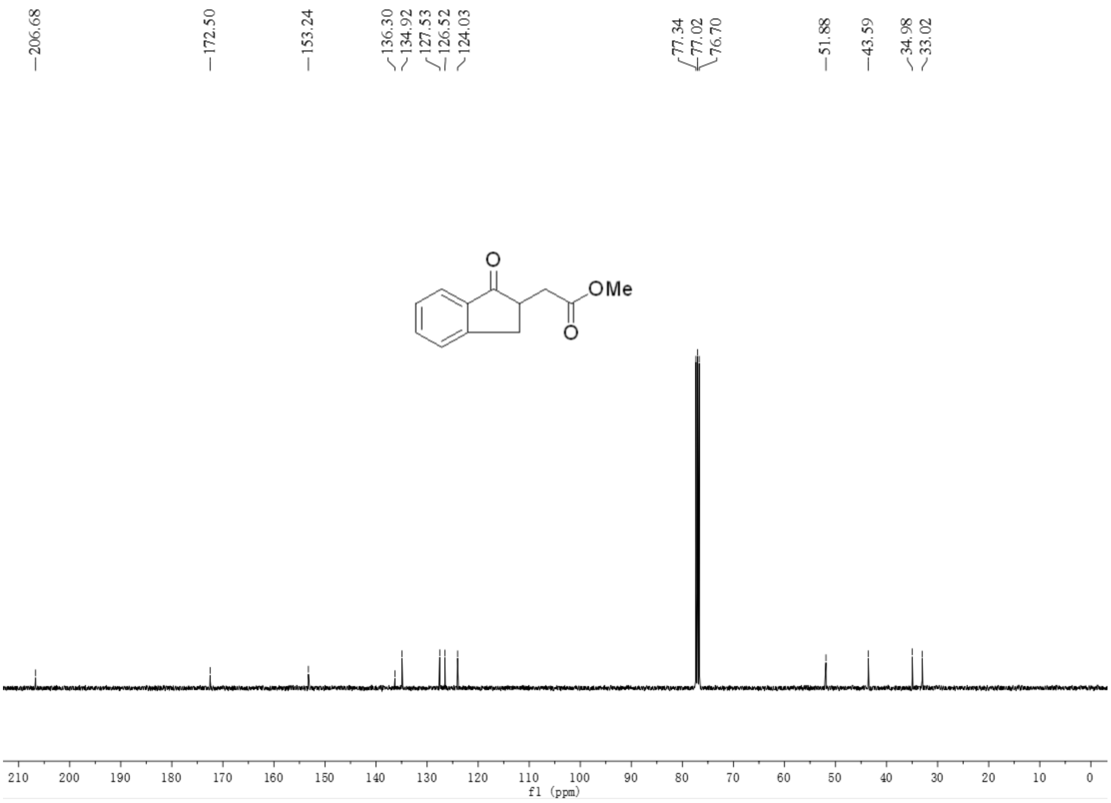

^{13}C spectra of **3sa**

ethyl 2-(4-oxochroman-3-yl)acetate **(3ab)**

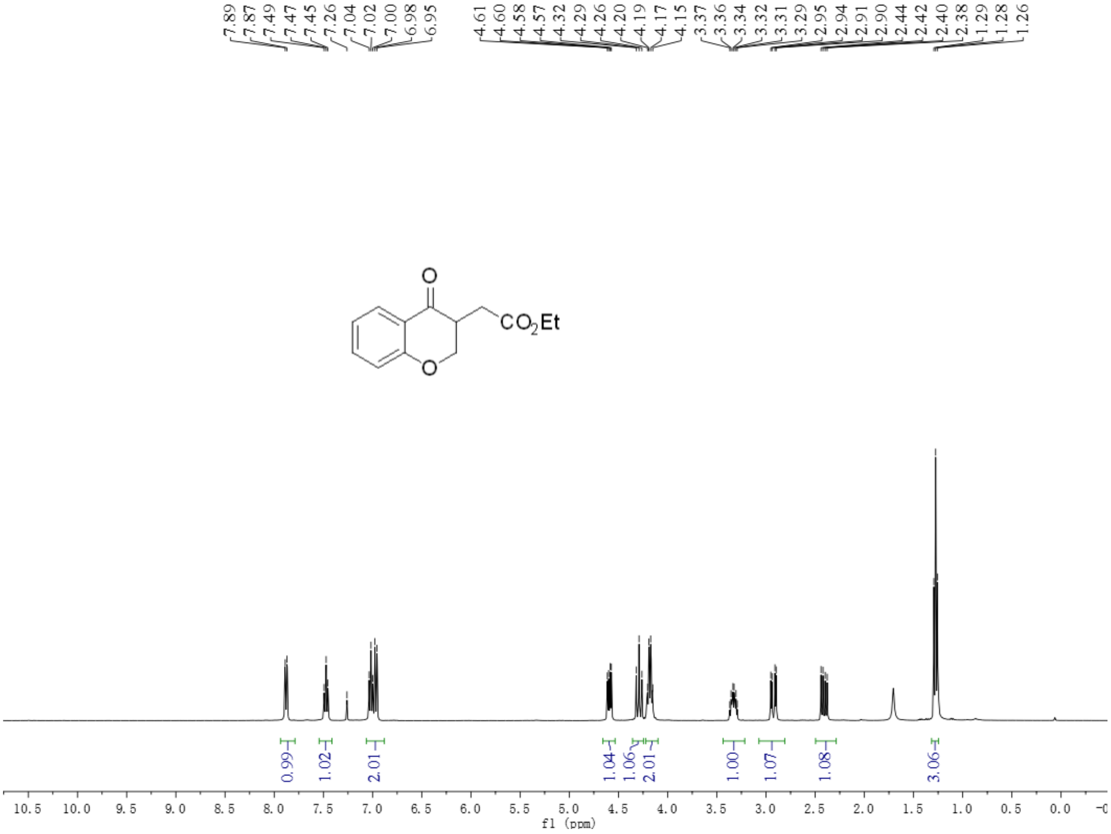

^{1}H spectra of **3ab**

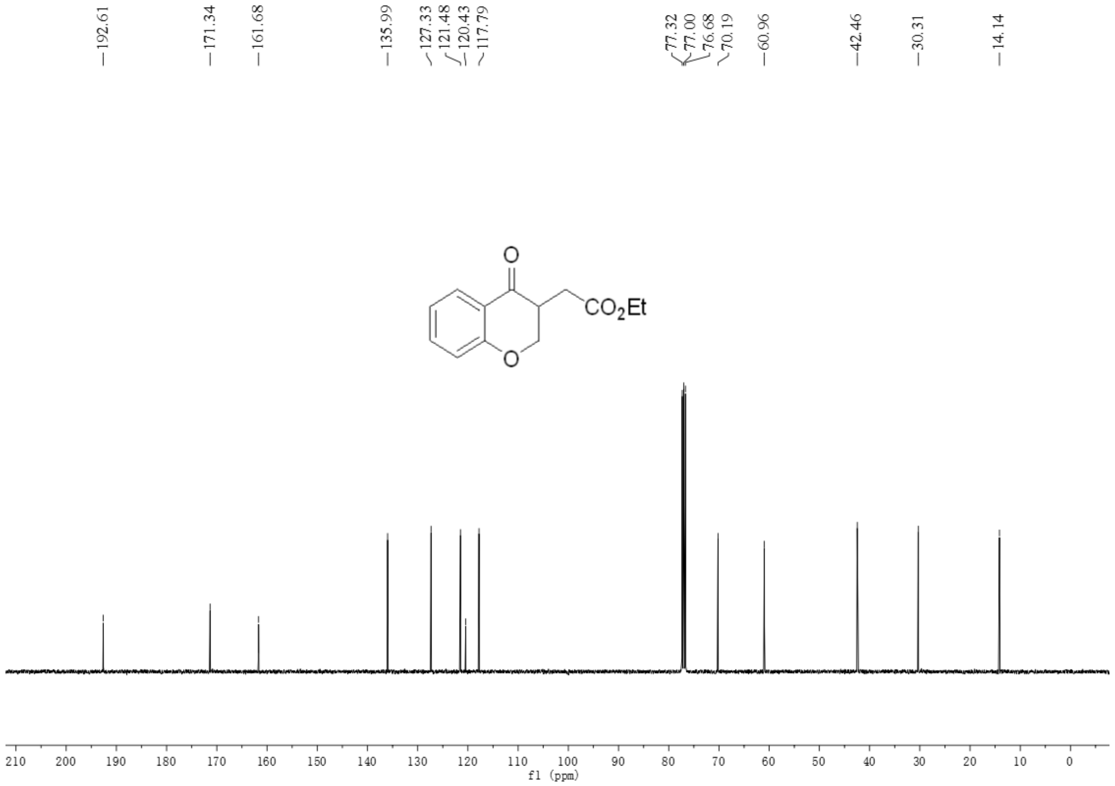

^{13}C spectra of **3ab**

butyl 2-(4-oxochroman-3-yl)acetate **(3ac)**

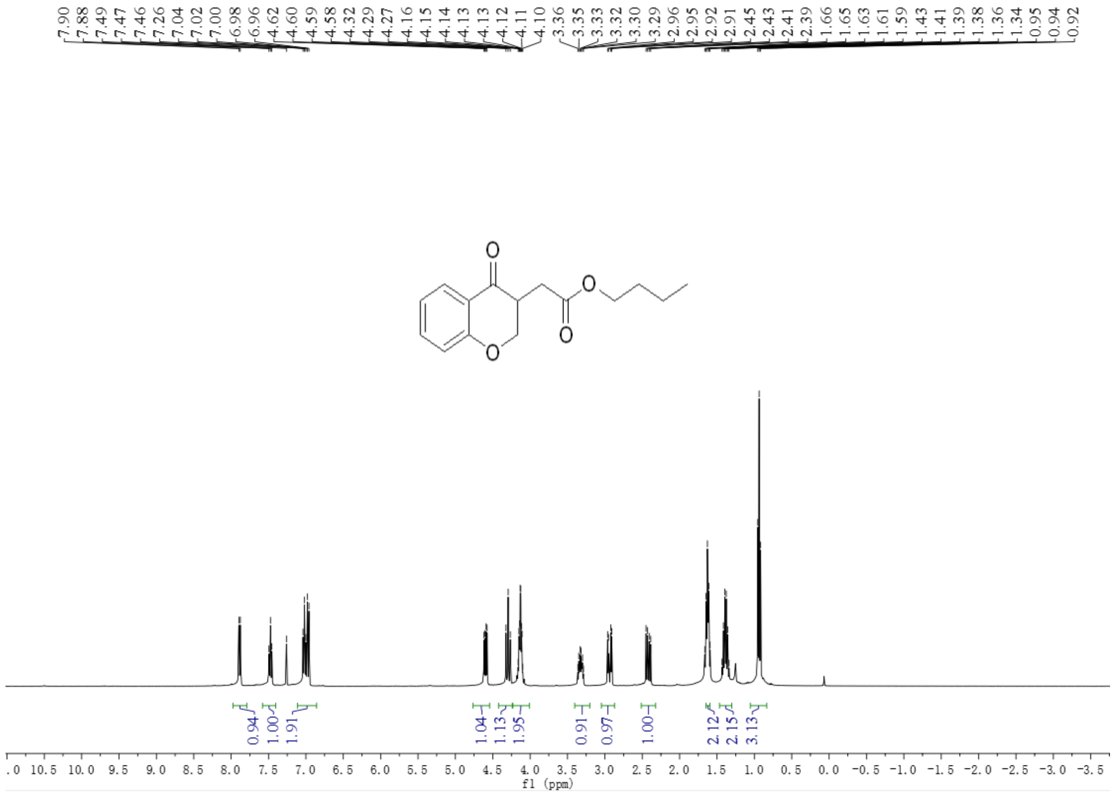

^{1}H spectra of **3ac**

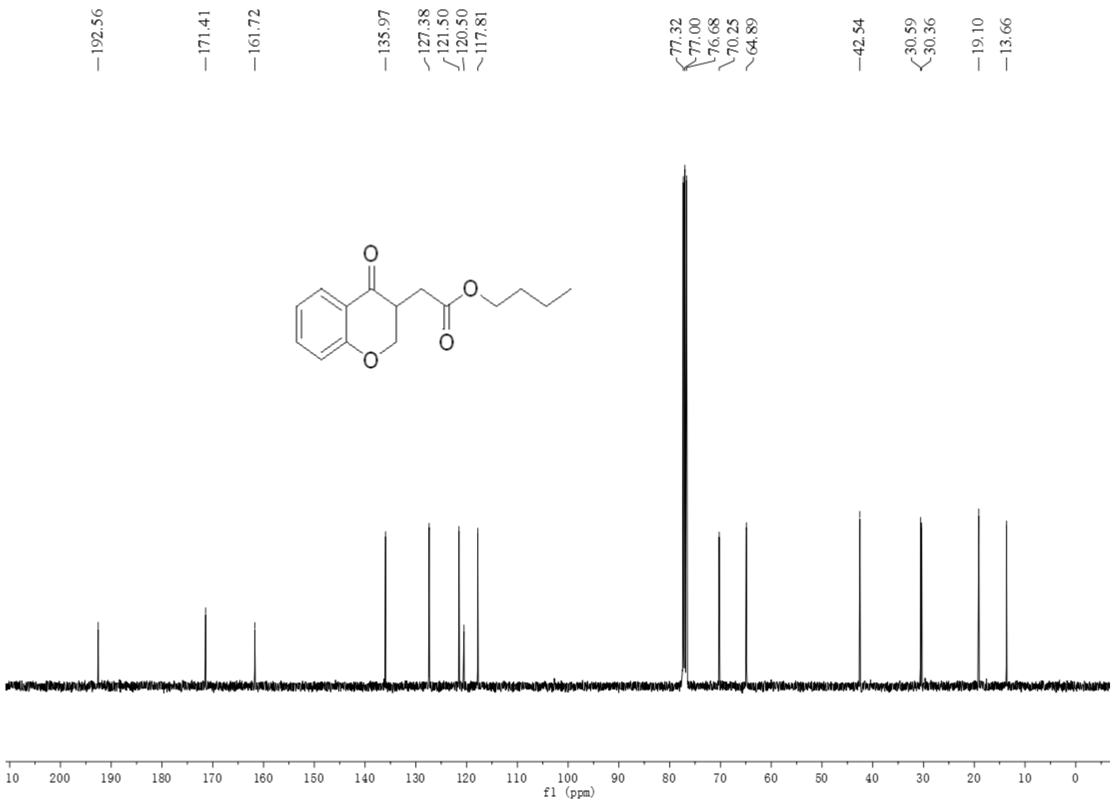

^{13}C spectra of **3ac**

***hexyl 2-(4-oxochroman-3-yl)acetate* (3ad)**

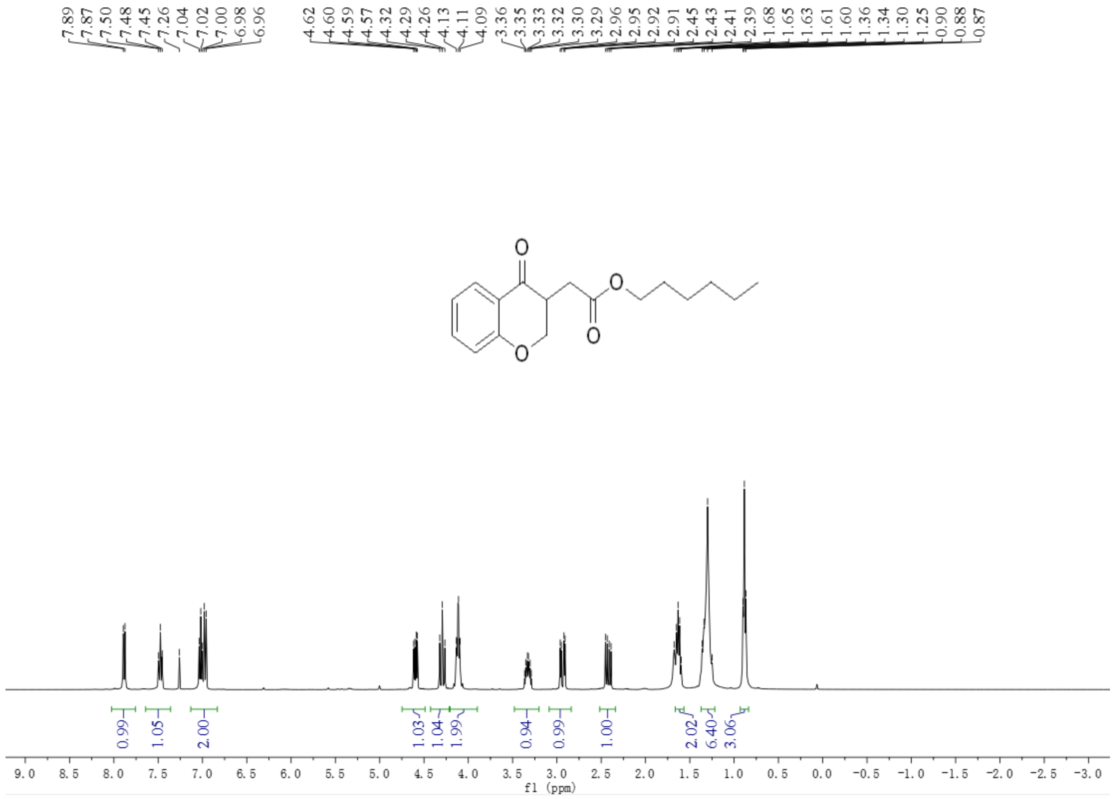

^{1}H spectra of **3ad**

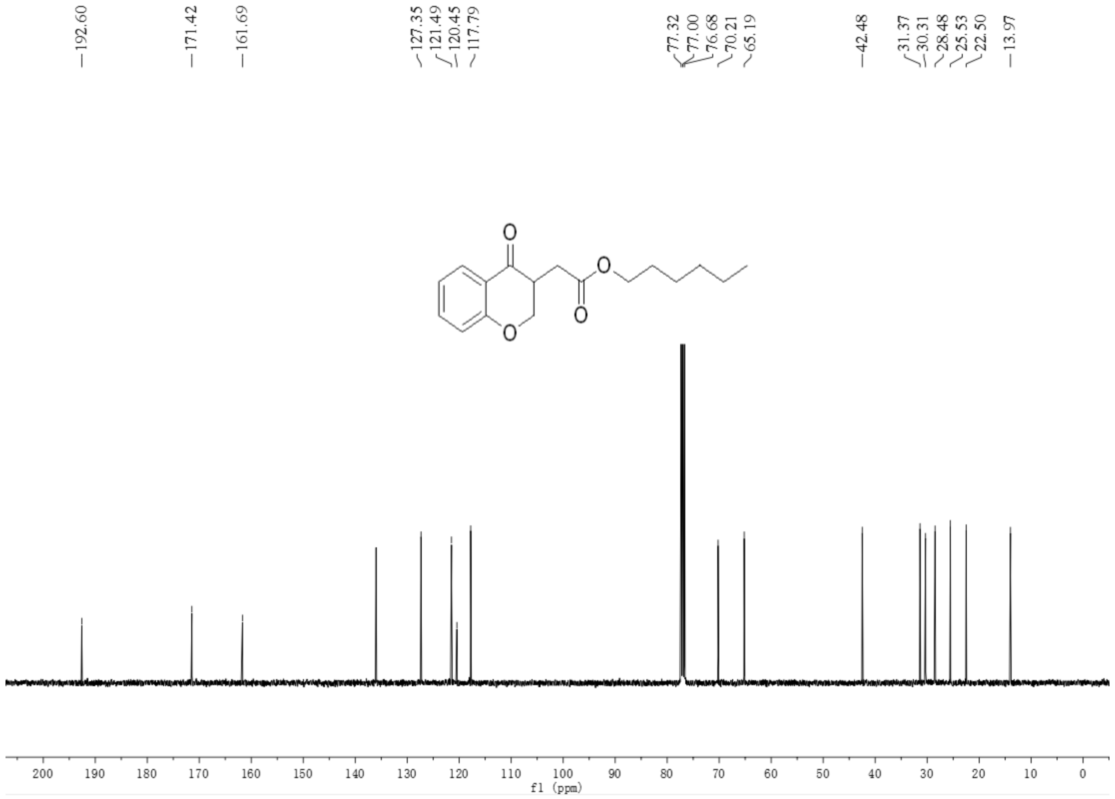

^{13}C spectra of **3ad**

***isobutyl 2-(4-oxochroman-3-yl)acetate* (3ae)**

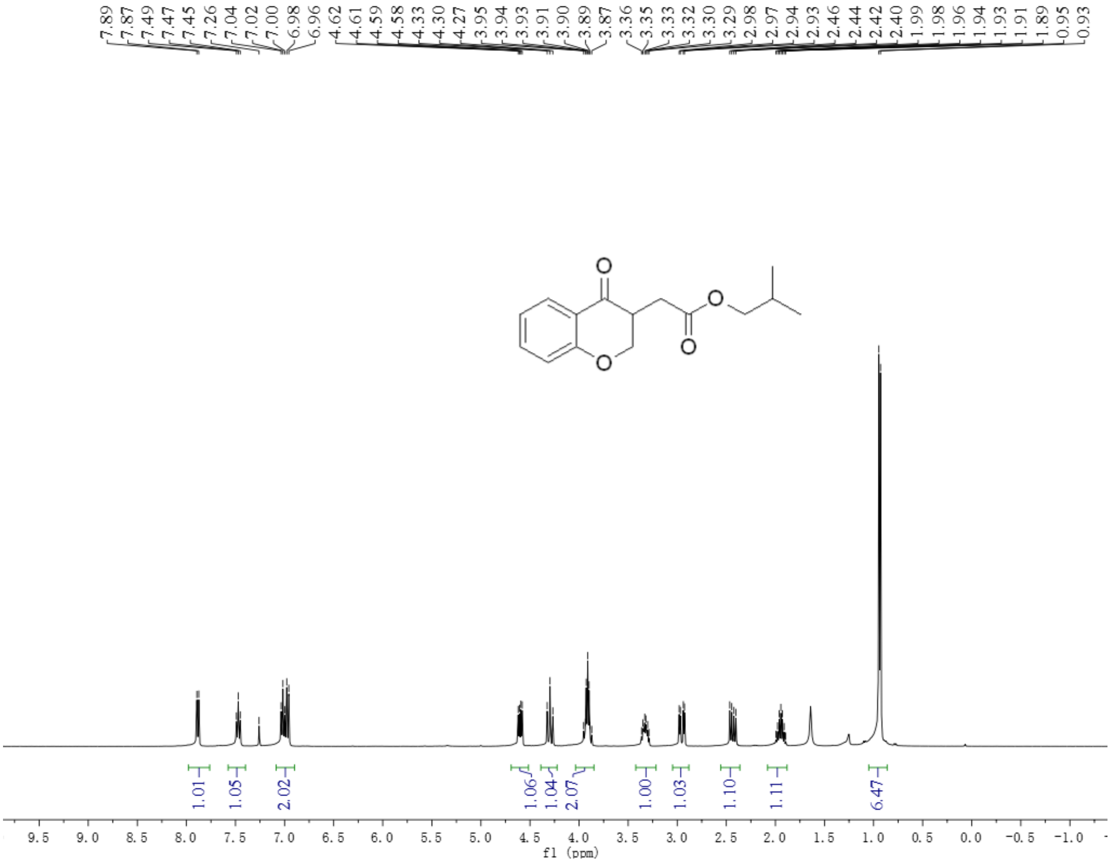

^{1}H spectra of **3ae**

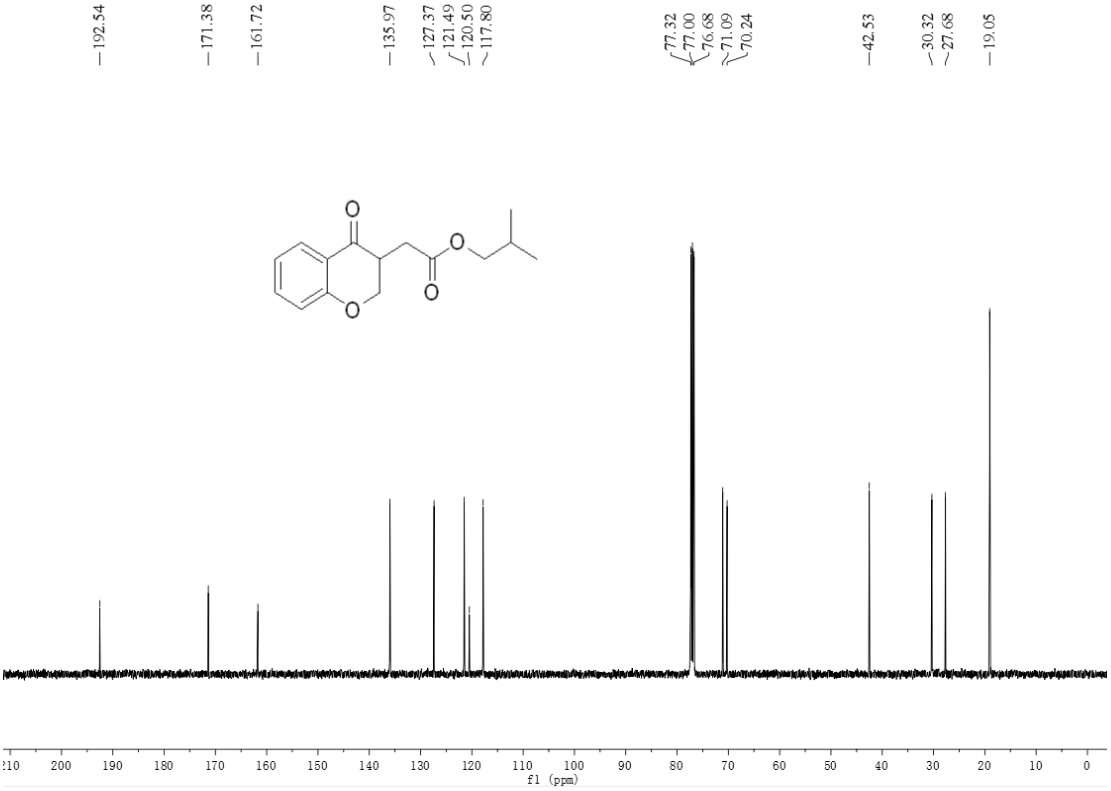

^{13}C spectra of **3ae**

3-methylpentyl 2-(4-oxochroman-3-yl)acetate (**3af**)

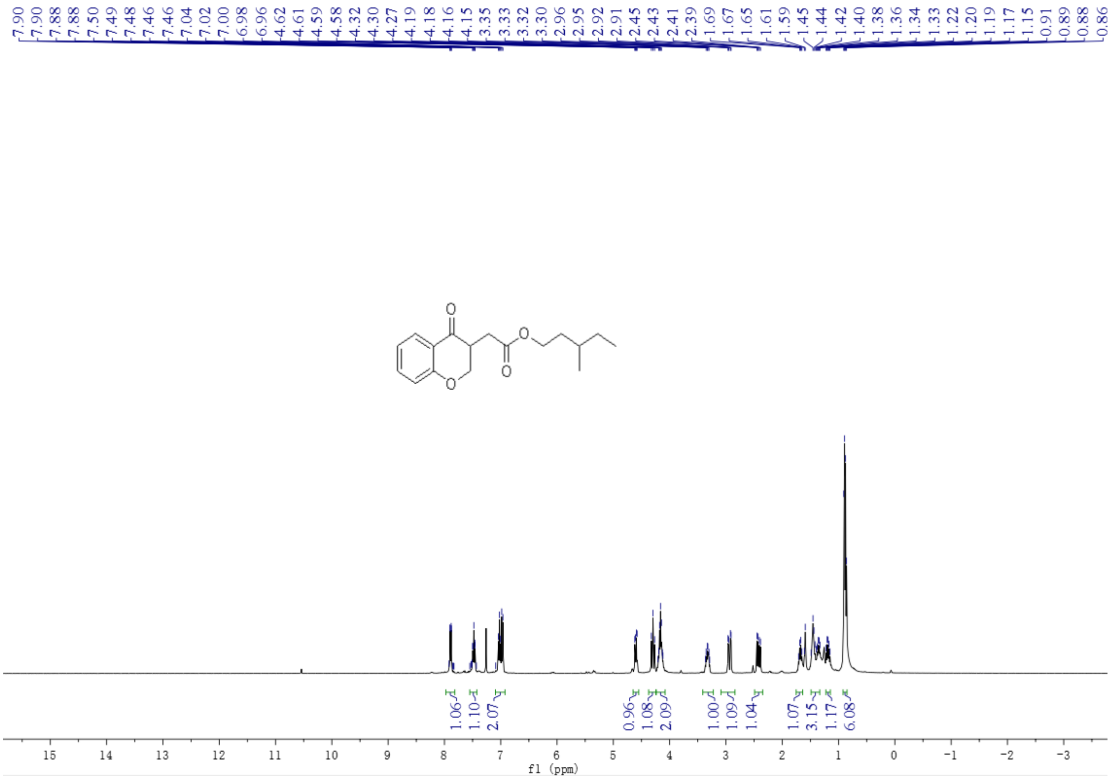

^{1}H spectra of **3af**

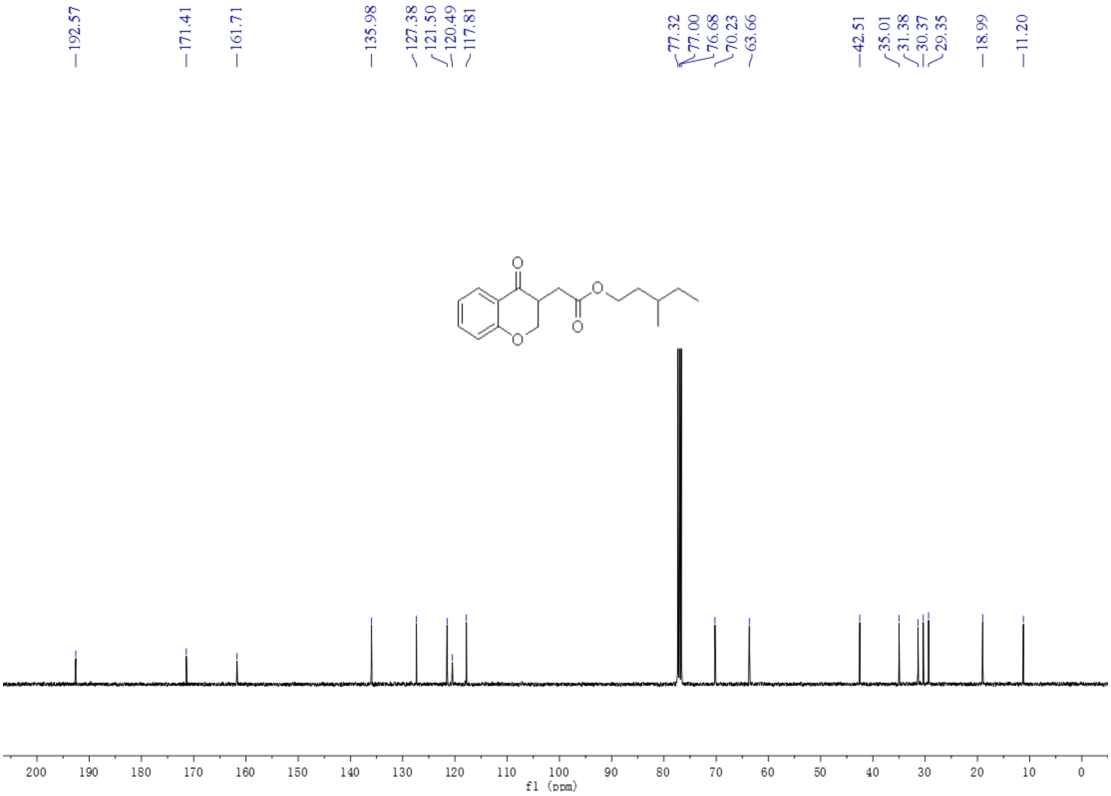

^{13}C spectra of **3af**

3-phenylpropyl 2-(4-oxochroman-3-yl)acetate **(3ag)**

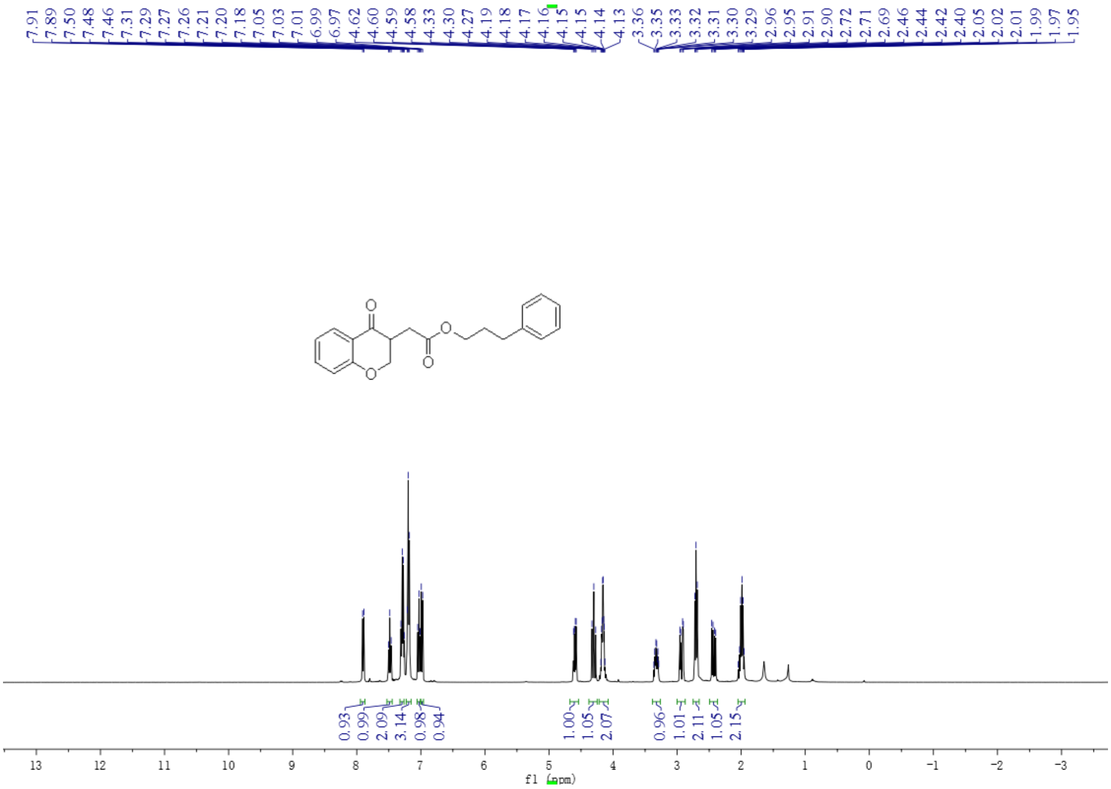

^{1}H spectra of **3ag**

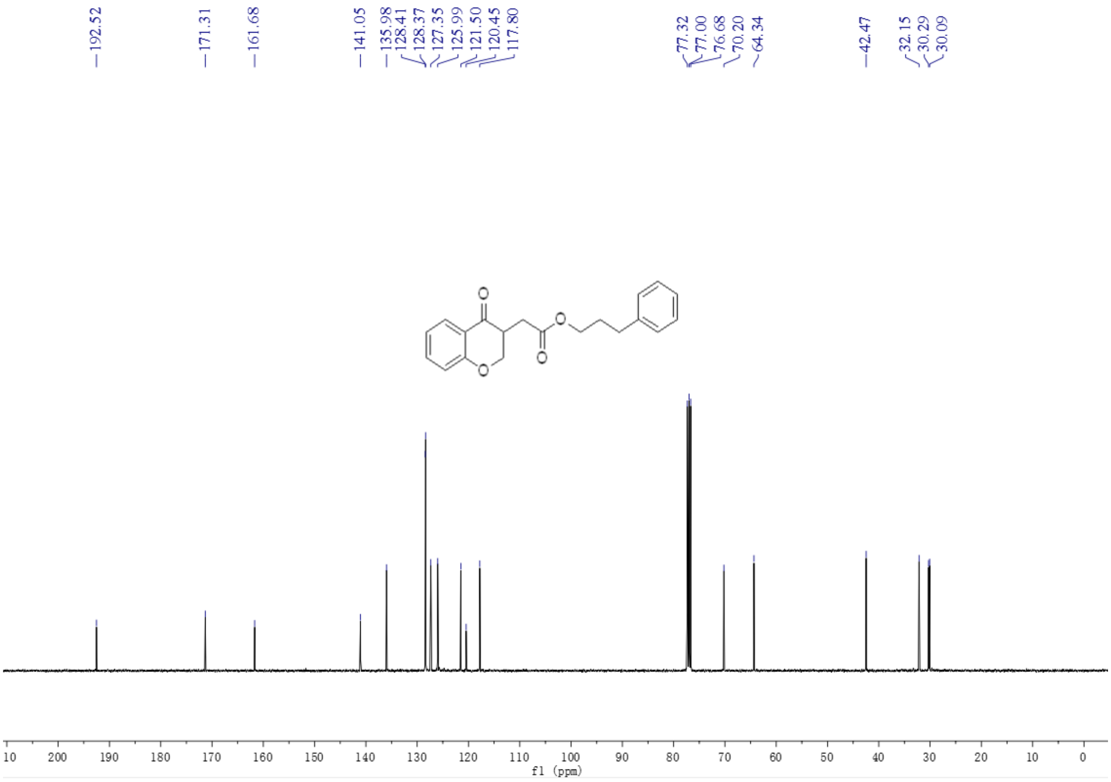

^{13}C spectra of **3ag**

***2-fluoroethyl 2-(4-oxochroman-3-yl)acetate* (3ah)**

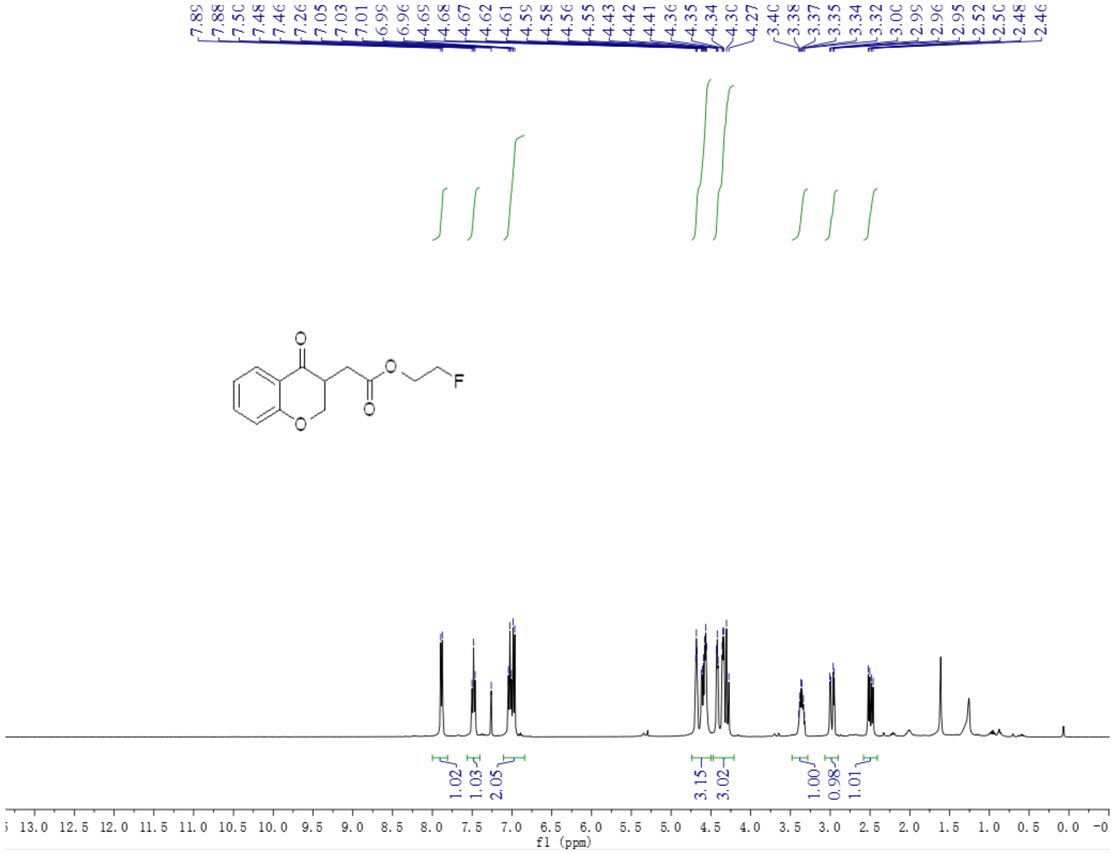

^{1}H spectra of **3ah**

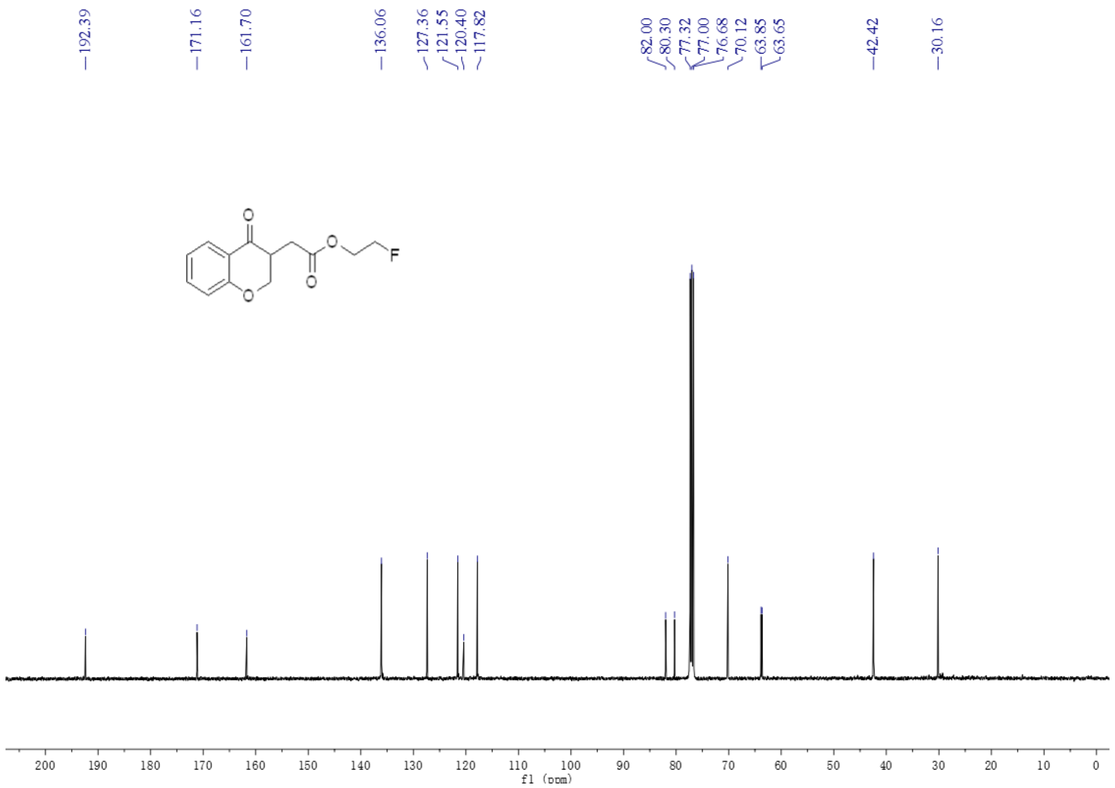

^{13}C spectra of **3ah**

***2-ethoxyethyl 2-(4-oxochroman-3-yl)acetate* (3ai)**

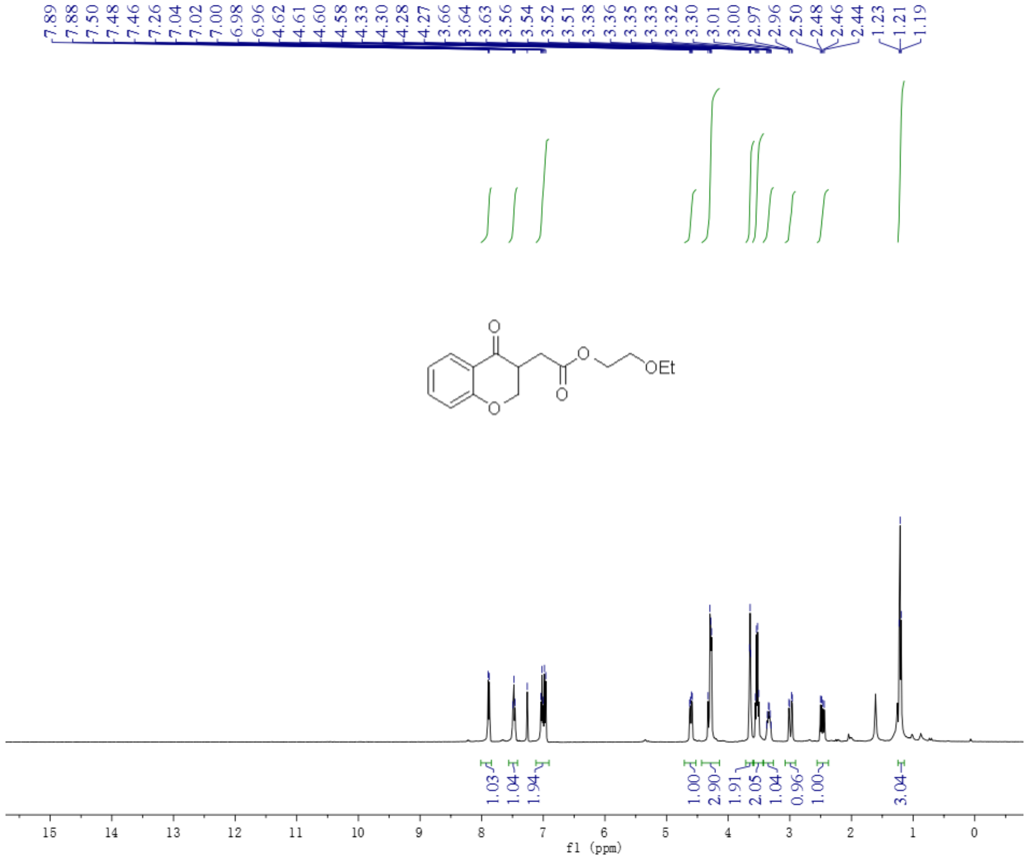

^{1}H spectra of **3ai**

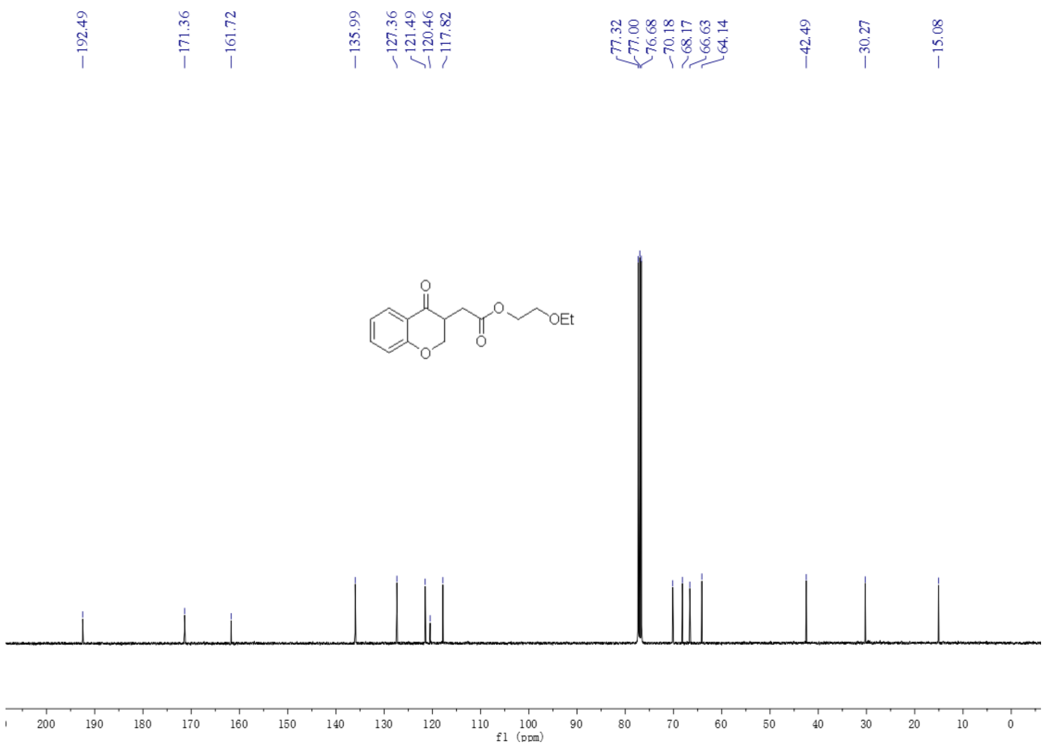

^{13}C spectra of **3ai**

***4,4,4-trifluorobutyl 2-(4-oxochroman-3-yl)acetate* (3aj)**

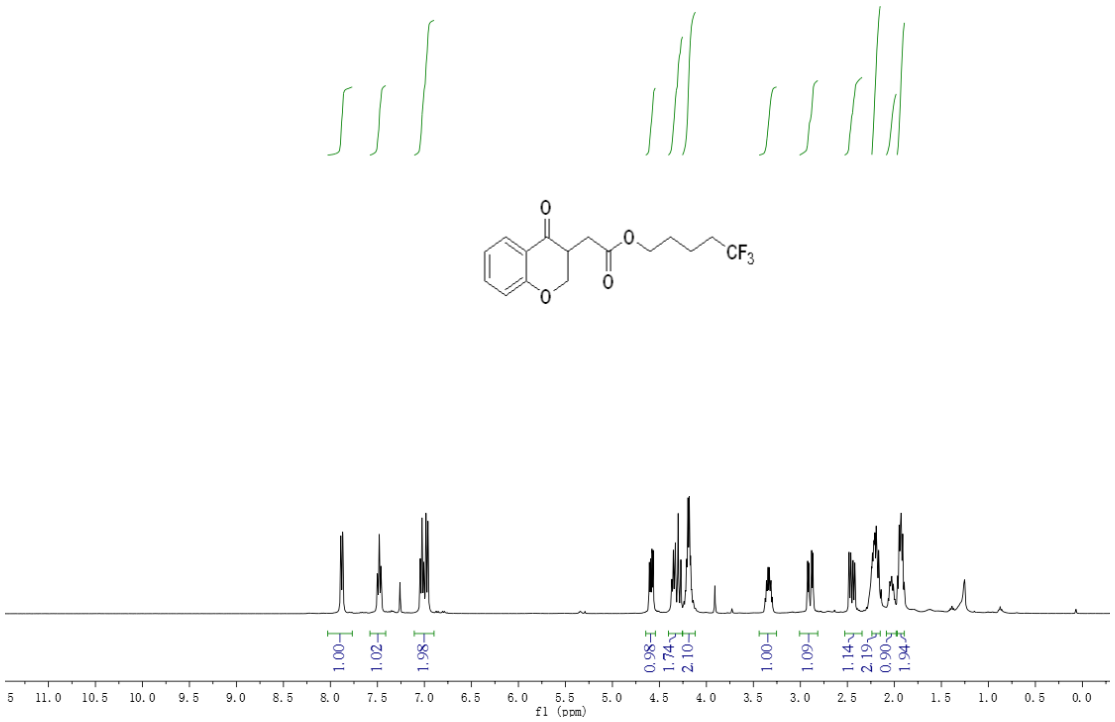

^{1}H spectra of **3aj**

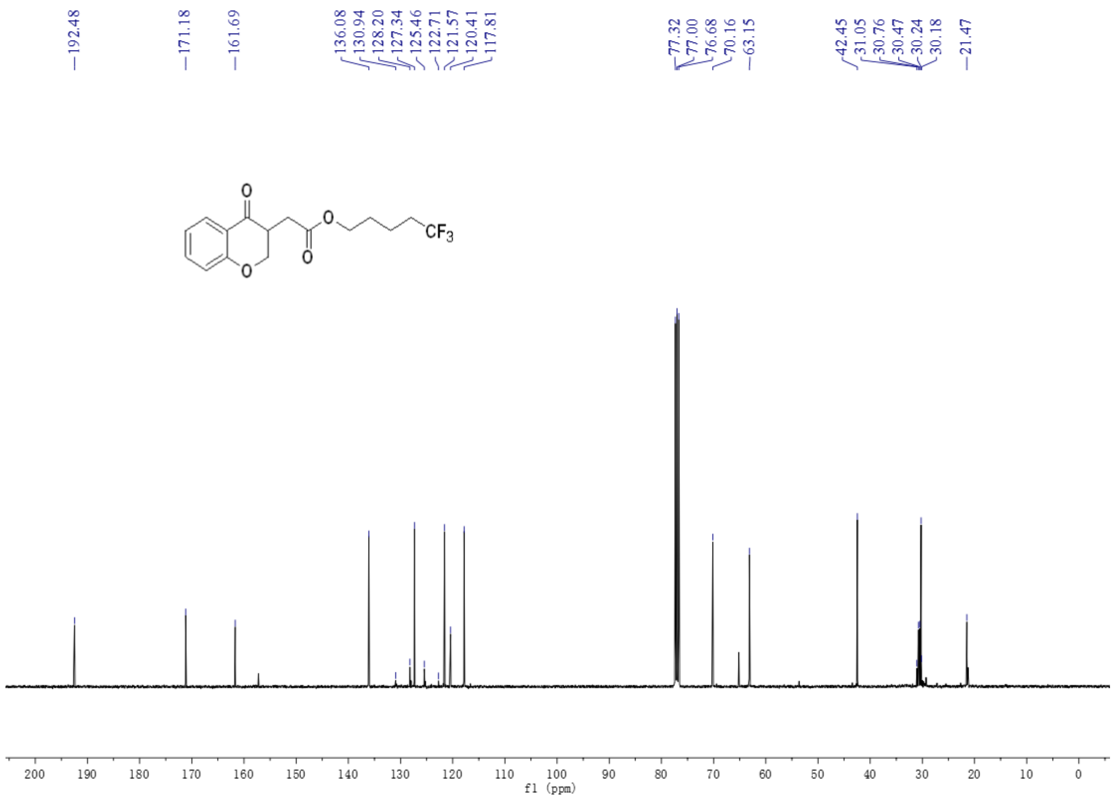

^{13}C spectra of **3aj**

***cyclobutyl 2-(4-oxochroman-3-yl)acetate* (3ak)**

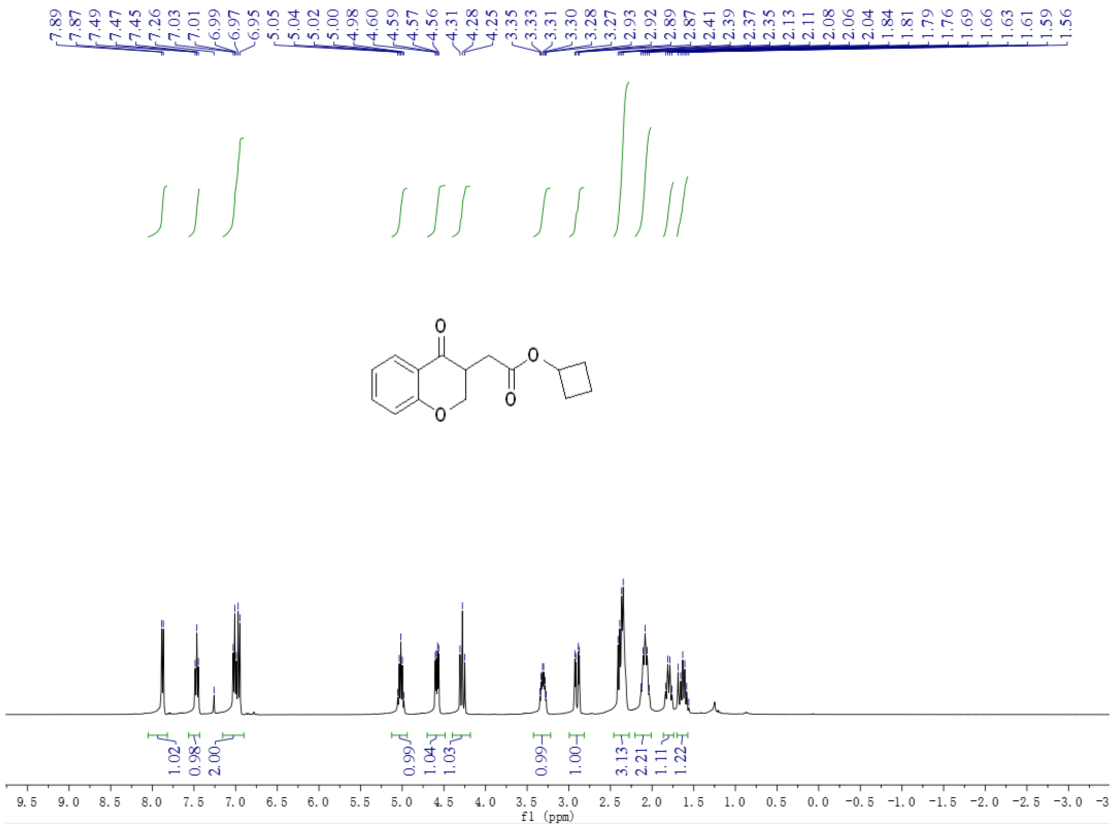

^{1}H spectra of **3ak**

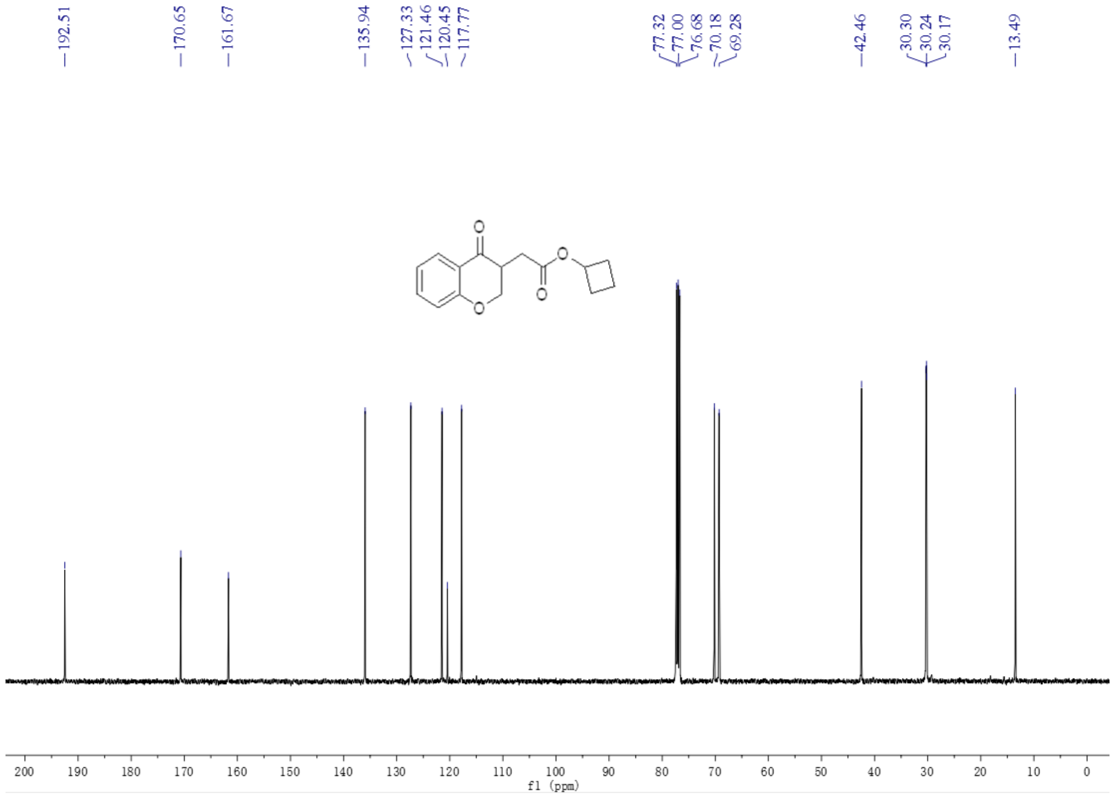

^{13}C spectra of **3ak**

***cyclohexyl 2-(4-oxochroman-3-yl)acetate* (3al)**

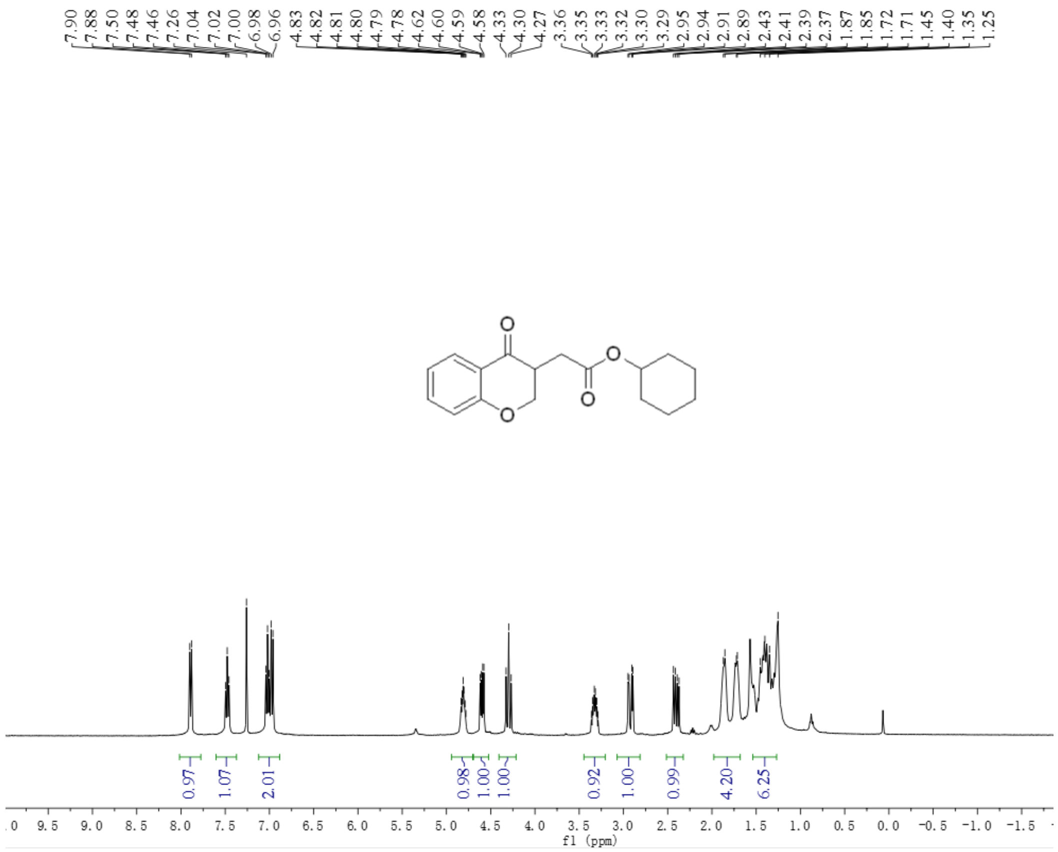

^{1}H spectra of **3al**

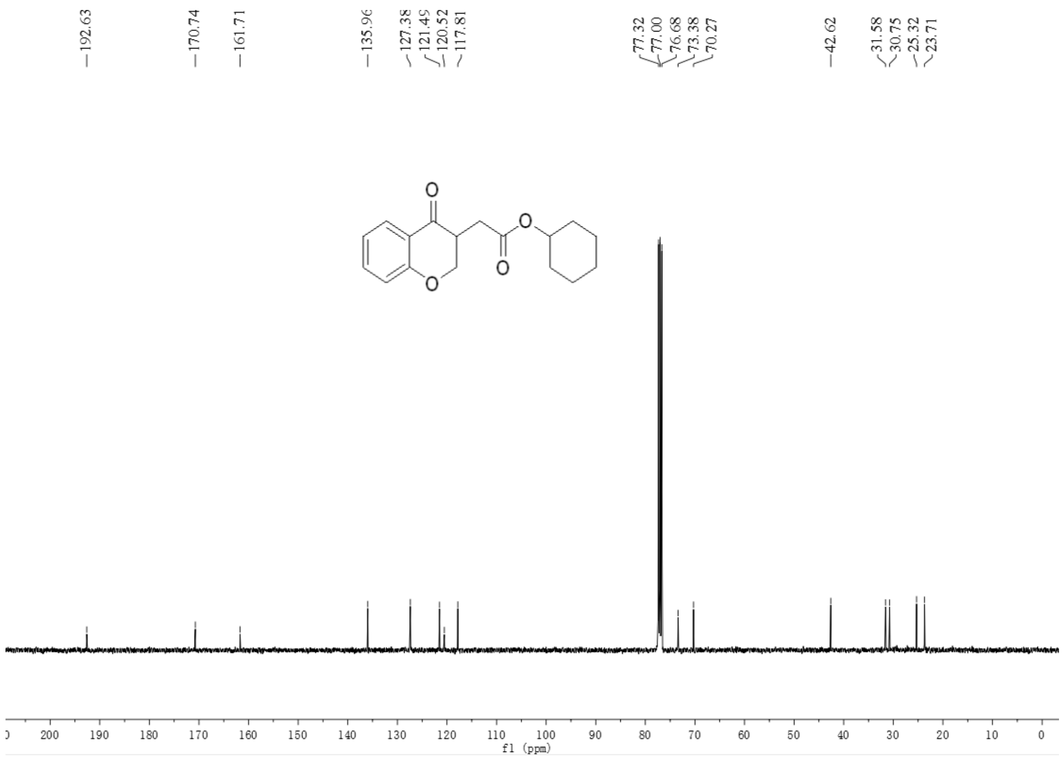

^{13}C spectra of **3al**

3-neopentylchroman-4-one (3am)

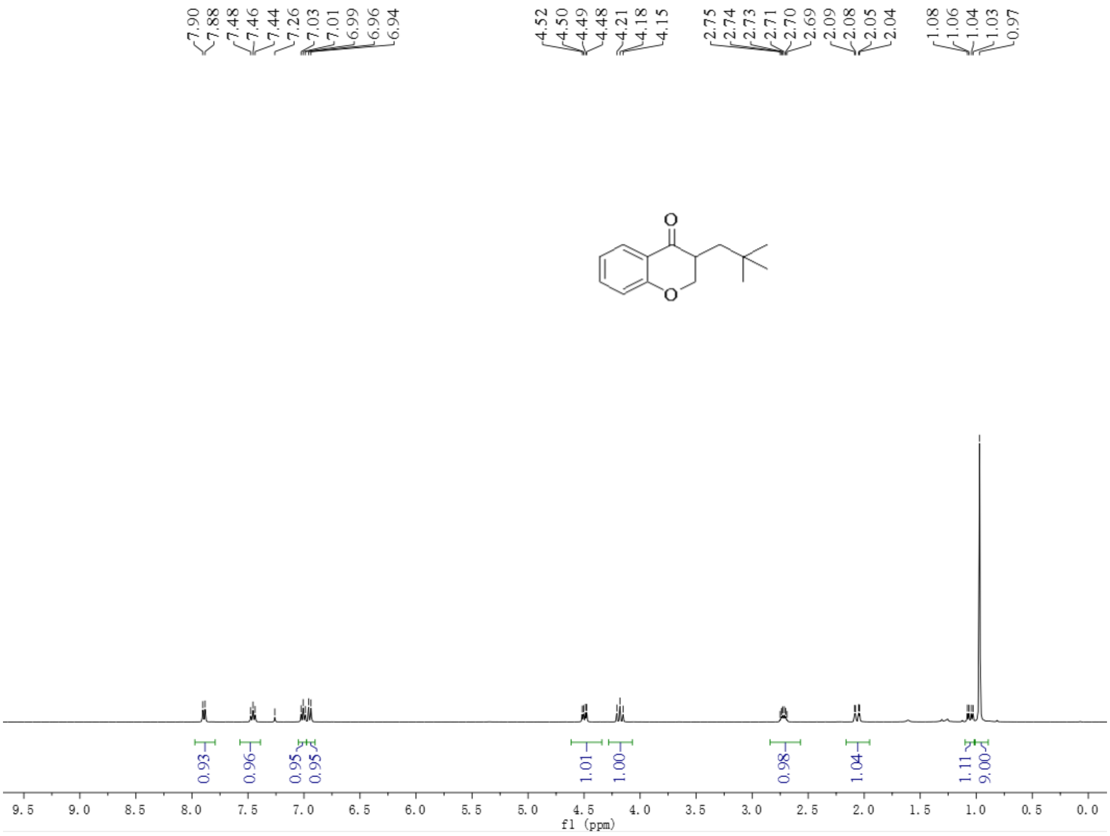

^{1}H spectra of **3am**

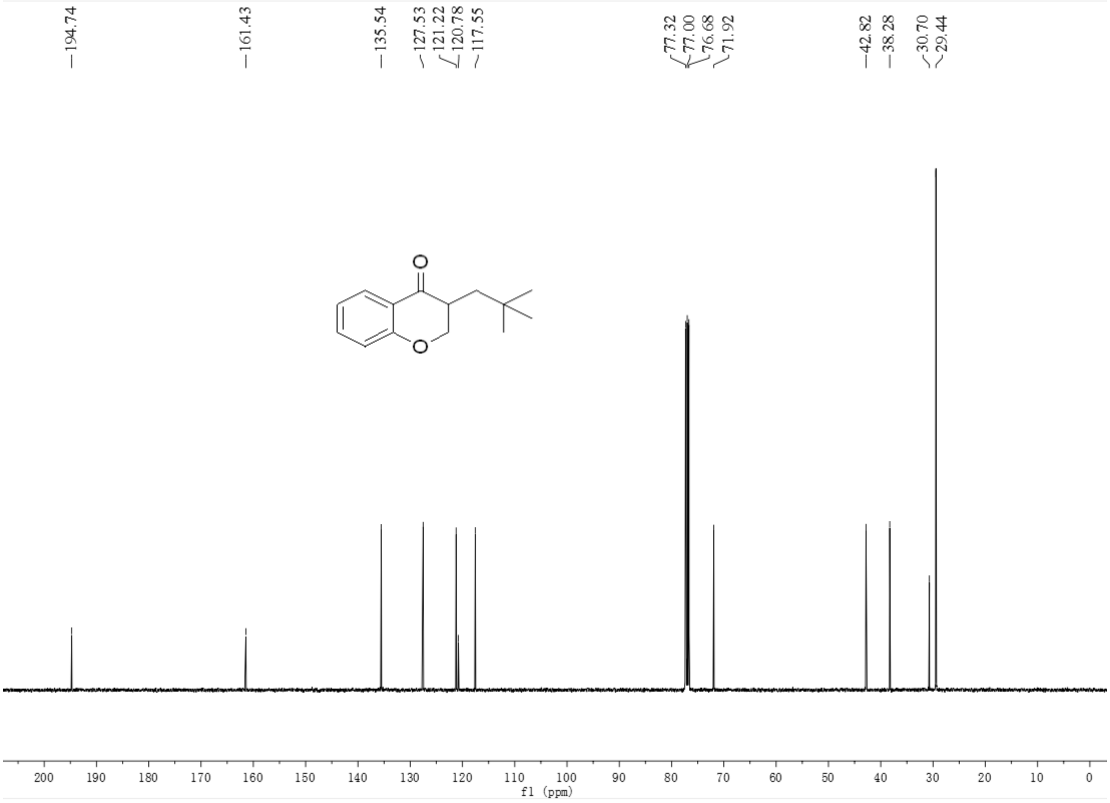

^{13}C spectra of **3am**

2-(4-oxochroman-3-yl)acetic acid (4a)

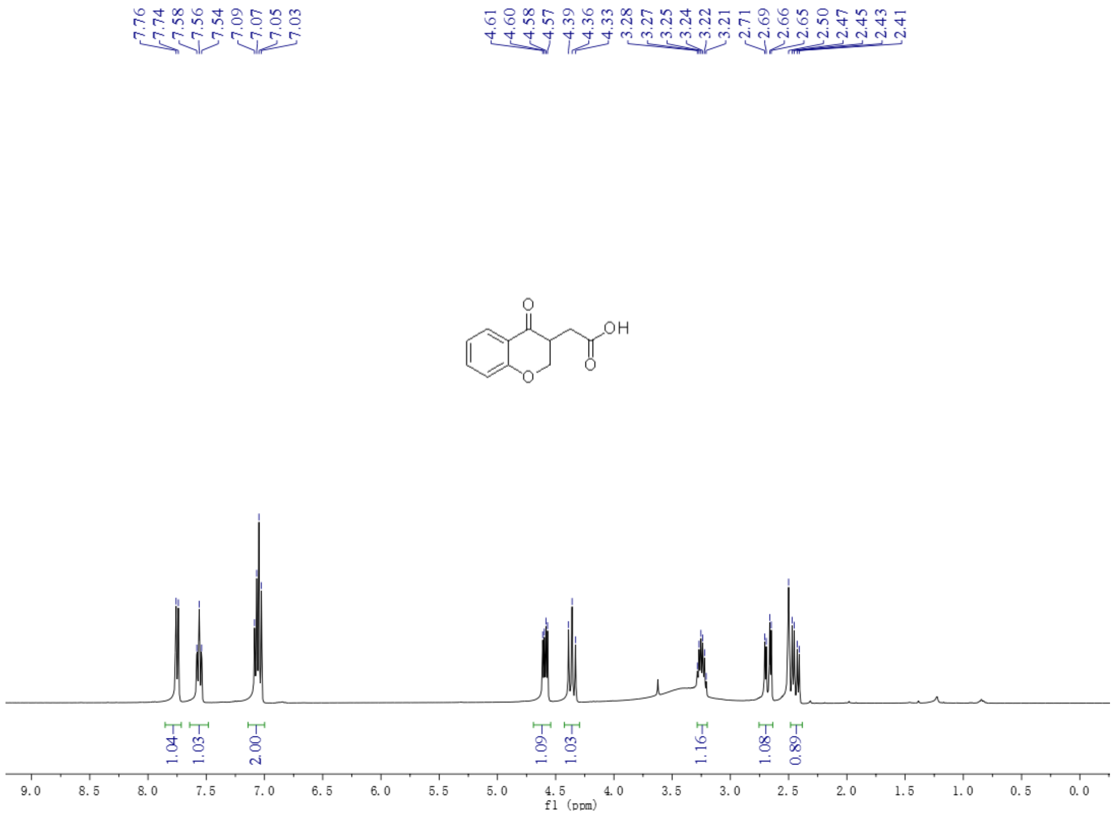

^{1}H spectra of **4a**

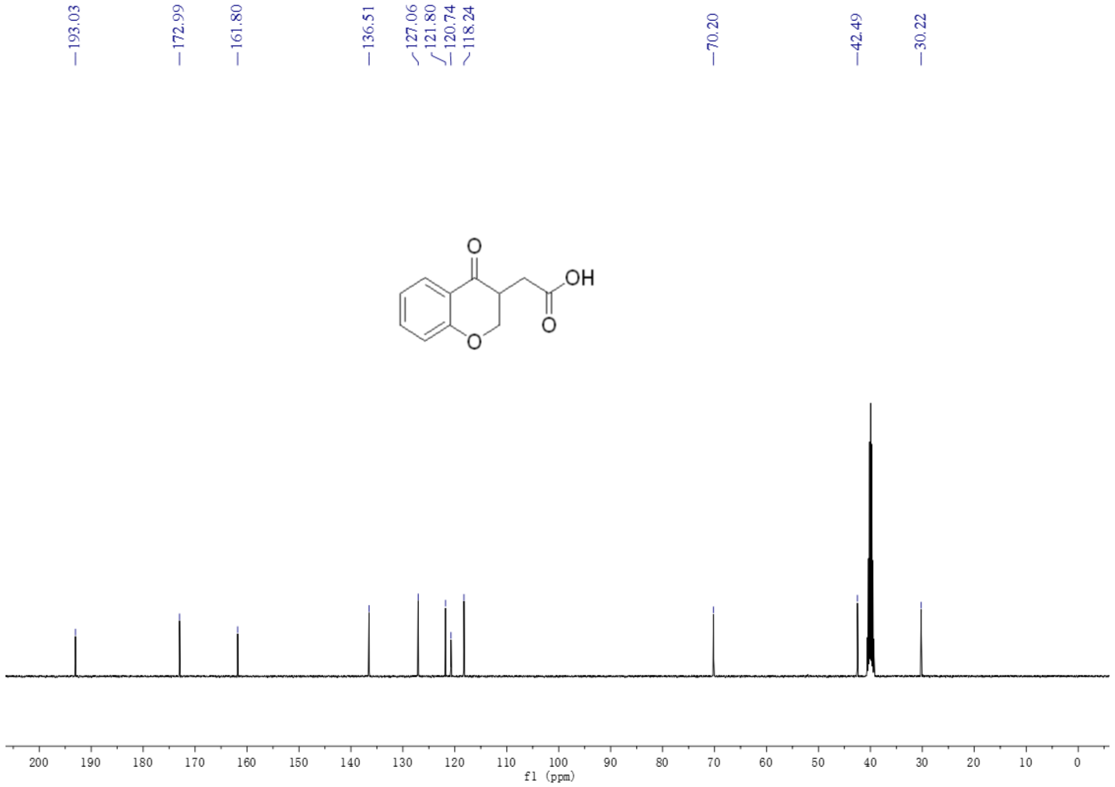

^{13}C spectra of **4a**

3a,9b-dihydro-4H-furo[3,2-c]chromen-2(3H)-one (5a)

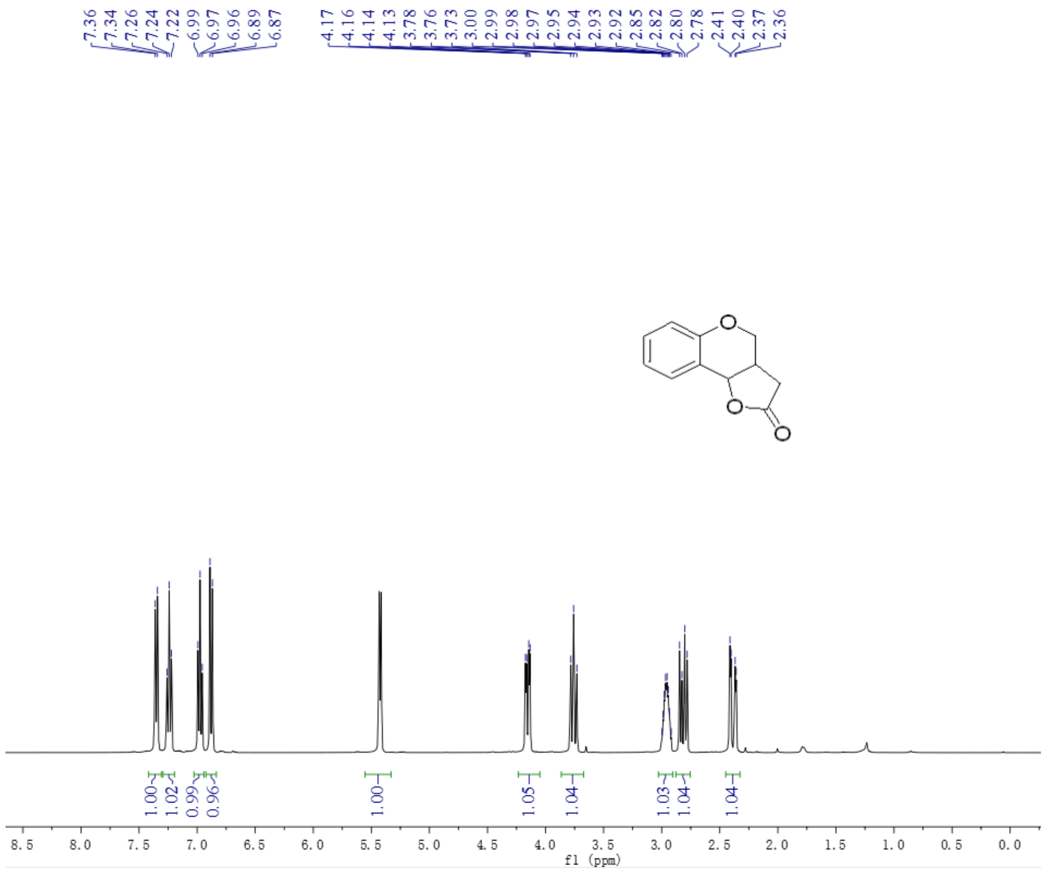

^{1}H spectra of **5a**

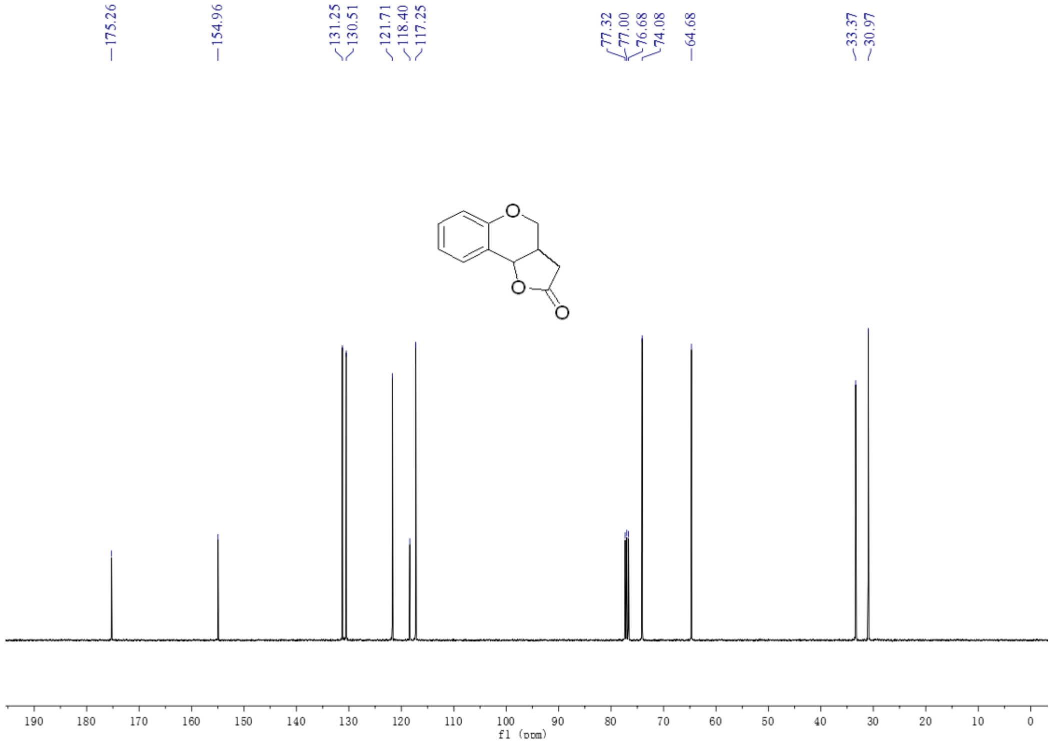

^{13}C spectra of **5a**
